# Stress response silencing by an E3 ligase mutated in neurodegeneration

**DOI:** 10.1038/s41586-023-06985-7

**Published:** 2024-01-31

**Authors:** Diane L. Haakonsen, Michael Heider, Andrew J. Ingersoll, Kayla Vodehnal, Samuel R. Witus, Takeshi Uenaka, Marius Wernig, Michael Rapé

**Affiliations:** 1 Department of Molecular and Cell Biology, University of California at Berkeley, Berkeley, CA USA; 2 Howard Hughes Medical Institute, University of California at Berkeley, Berkeley, CA USA; 3 Institute for Stem Cell Biology and Regenerative Medicine, Stanford University School of Medicine, Stanford, CA USA; 4 Department of Pathology, Stanford University School of Medicine, Stanford, CA USA; 5 California Institute for Quantitative Biosciences (QB3), University of California at Berkeley, Berkeley, CA USA

**Keywords:** Ubiquitylation, Ubiquitin ligases, Mitochondria

## Abstract

Stress response pathways detect and alleviate adverse conditions to safeguard cell and tissue homeostasis, yet their prolonged activation induces apoptosis and disrupts organismal health^1–3^. How stress responses are turned off at the right time and place remains poorly understood. Here we report a ubiquitin-dependent mechanism that silences the cellular response to mitochondrial protein import stress. Crucial to this process is the silencing factor of the integrated stress response (SIFI), a large E3 ligase complex mutated in ataxia and in early-onset dementia that degrades both unimported mitochondrial precursors and stress response components. By recognizing bifunctional substrate motifs that equally encode protein localization and stability, the SIFI complex turns off a general stress response after a specific stress event has been resolved. Pharmacological stress response silencing sustains cell survival even if stress resolution failed, which underscores the importance of signal termination and provides a roadmap for treating neurodegenerative diseases caused by mitochondrial import defects.

## Abstract (web summary)

The E3 ligase SIFI is identified as a dedicated silencing factor of the integrated stress response, a finding that has implications for the development of therapeutics for neurodegenerative diseases caused by mitochondrial protein import stress.

## Main

All cells in our bodies must navigate dynamic environments that expose them to toxins, temperature fluctuations or nutrient limitations. They survive these adverse conditions by relying on conserved signalling pathways known as stress responses^1–4^. These pathways often modulate basic processes, such as cell division, mRNA translation and metabolism, to provide cells with time and resources to repair the damage^1^.

Although transient stress response activation enables cells to cope with damage, persistent signalling indicates that a deleterious situation cannot be resolved. Prolonged stress response activation accordingly triggers apoptotic programmes that eliminate irreversibly damaged and potentially tumorigenic cells^5–8^. When cells face persistent stress during ageing or in disease^5,7^, continuous stress response signalling can induce unwanted cell death and compromise tissue integrity with devastating consequences for organismal health. Stress response pathways must therefore be silenced as soon as conditions improve, but how this occurs remains poorly understood.

Here we report that stress response silencing is an active and regulated process that is tightly linked to human disease. The response to mitochondrial protein import stress is terminated through a large E3 ligase that is mutated in ataxia and in early-onset dementia: SIFI. SIFI acts by inducing the proteasomal degradation of both unimported mitochondrial precursors and stress response components, which it recognizes through shared sequence motifs that equally encode protein localization and stability. Although inactivation of SIFI causes accumulation of aggregation-prone proteins, pharmacological restoration of stress response silencing was sufficient to restore the survival of SIFI mutant cells. Our work therefore provides a mechanistic basis for timely stress response silencing and points to new approaches for treating neurodegenerative diseases caused by mitochondrial import defects.

## SIFI functions upon mitochondrial stress

**Fig. 1:**
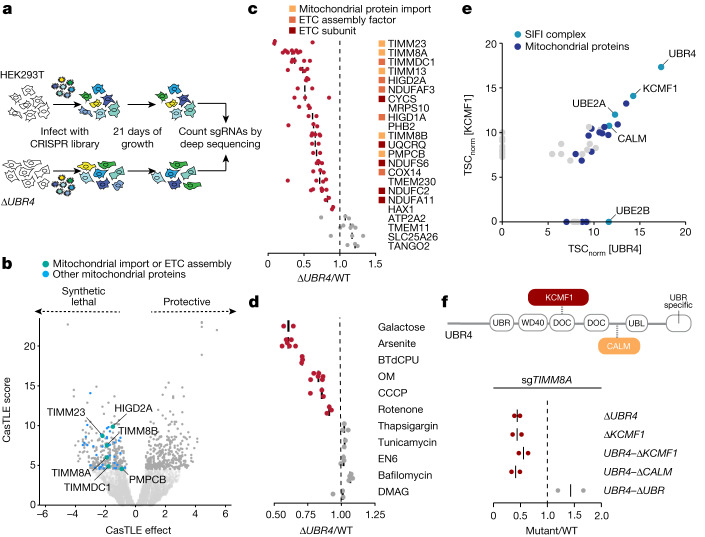
The E3 ligase SIFI protects cells during mitochondrial import stress. **a**, Outline of the synthetic lethality screen. sgRNA, singe guide RNA. **b**, Δ*UBR4* cells are sensitive to the inhibition of mitochondrial import or ETC function. Darker grey dots represent the top 5% CasTLE score genes. **c**, Screen validation by depleting hits in mixtures of GFP-labelled WT and mCherry-labelled Δ*UBR4* cells, reported as (Δ*UBR4*_sgRNA_/WT_sgRNA_)/(Δ*UBR4*_sgCNTRL_/WT_sgCNTRL_). sgCNTRL, control sgRNA. **d**, Chemical mitochondrial stress or growth in galactose-depleted conditions selectively depletes Δ*UBR4* cells, reported as (Δ*UBR4*_treatment_/WT_treatment_)/(Δ*UBR4*_control_/WT_control_). **e**, Endogenous Flag–UBR4 and KCMF1–Flag were affinity purified, and binding partners were determined by mass spectrometry. TSC_norm_, normalized total spectral counts. **f**, Cells lacking KCMF1 or the endogenous KCMF1-binding, calmodulin-binding (CALM) or UBR domains of UBR4 were depleted of TIMM8A and assessed by competition, reported as (UBR4(Δdomain)_sg*TIMM8A*_/WT_sg*TIMM8A*_)/(UBR4(Δdomain)_sgCNTRL_/WT_sgCNTRL_). UBR4 domain map visualizing location of endogenous domain deletions.

**Extended Data Fig. 1:**
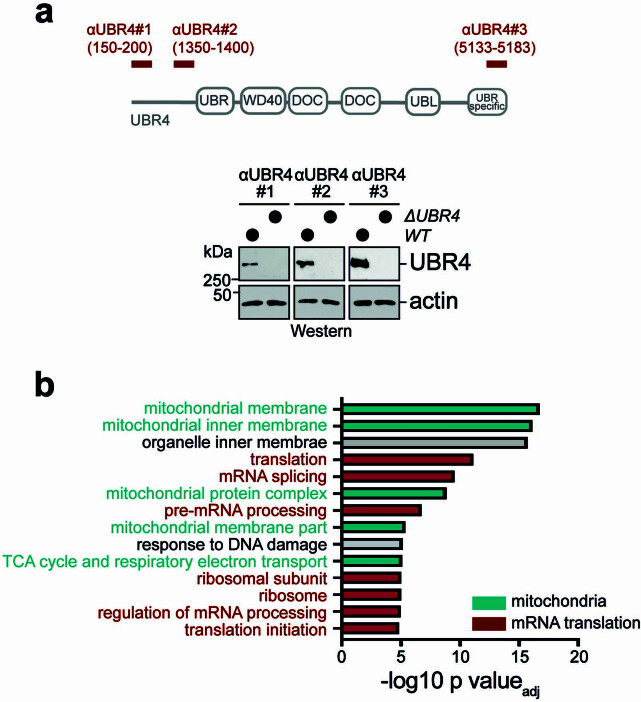
*UBR4* deletion sensitizes cells to mitochondrial protein import stress. **a**. Western blot analysis shows deletion of endogenous *UBR4* from HEK293T cells as a prerequisite for a subsequent whole genome synthetic lethality CRISPR screen. Three different antibodies directed against UBR4 were used to establish successful deletion. **b**. GO analysis of genetic interactors of UBR4. Screen hits in the top 5% CasTLE score and with negative CasTLE effects were run through ClueGO to identify enriched pathways that are synthetic lethal with UBR4 deletion. P values were generated by ClueGO (Fisher’s exact test and Bonferroni correction). For gel source data, see Supplementary Fig. 1.

We recently discovered that UBR4, an E3 ligase known for its role in the N-end rule pathway^9^, helps degrade aggregation-prone nascent polypeptides^10^. As mutations in *UBR4* cause ataxia and early-onset dementia^11–13^, we asked whether the quality control function of UBR4 safeguards specific pathways to ensure cellular homeostasis. We therefore generated Δ*UBR4* cells (Extended Data Fig. 1a) and used them in a whole genome CRISPR–Cas9 synthetic lethality screen to reveal genetic backgrounds that depend on this E3 ligase (Fig. 1a and Supplementary Table 1).

UBR4 was particularly important when mitochondrial function was compromised (Fig. 1b and Extended Data Fig. 1b). Most genetic interactors of *UBR4* controlled mitochondrial protein import (*TIMM8A*, *TIMM8B*, *TIMM23* and *PMPCB*) or the biogenesis and function of the electron transport chain (ETC) (*TIMMDC1*, *HIGD2A* and subunits of ETC complexes) (Fig. 1b), which is required for the transport of nuclear-encoded nascent polypeptides into mitochondria^14^. Notably, mutations in the genetic interactors *TIMM8A*, *PMPCB*, *NDUFAF3*, *NDUFA11*, *NDUFC2* or *NDUFS6* cause Mohr–Tranebjærg syndrome, childhood ataxia or Leigh syndrome. These neurodegenerative diseases manifest similar symptoms to those seen in patients with *UBR4* mutations^15–17^.

Validating our screen results, loss of mitochondrial import factors or ETC components depleted mCherry-labelled Δ*UBR4* cells from mixtures with GFP-labelled wild-type (WT) cells (Fig. 1c). Δ*UBR4* cells were also sensitive to chemicals that induce mitochondrial stress, such as CCCP, oligomycin, BTdCPU and arsenite, and they were depleted when grown on galactose to enforce mitochondrial ATP production (Fig. 1d). Compounds that compromise the integrity of the endoplasmic reticulum or lysosome had no specific effects on Δ*UBR4* cells (Fig. 1d).

**Extended Data Fig. 2:**
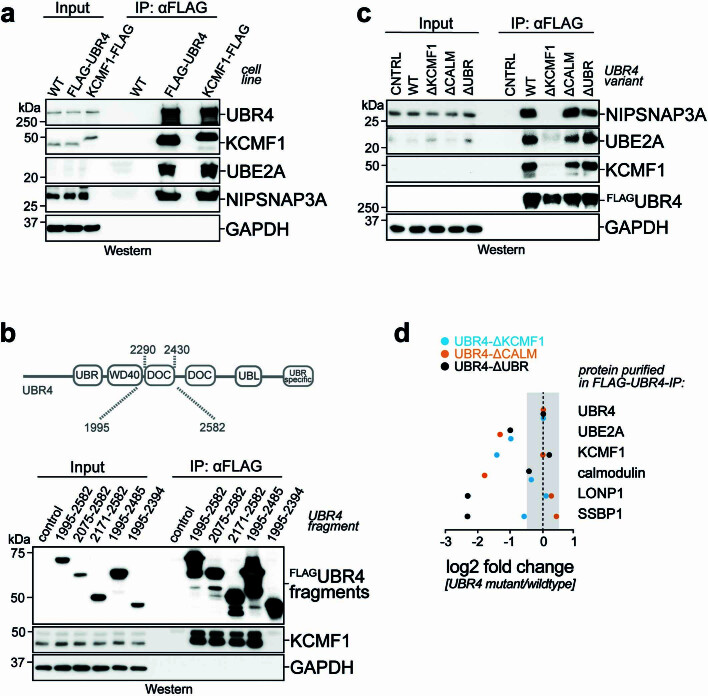
UBR4 stably interacts with KCMF1. **a**. Endogenously FLAG-tagged UBR4 and KCMF1 were affinity-purified from 293T cells, and bound proteins were detected by Western blotting using specific antibodies. Similar results in n = 3 independent experiments. **b**. KCMF1 binds to a DOC domain in UBR4. FLAG-tagged fragments of UBR4 were immunoprecipitated from *ΔUBR4* cells and co-precipitating endogenous KCMF1 was detected by Western blotting. Similar results in n = 2 independent experiments. **c**. Validation of the KCMF1 domain in endogenous UBR4. The DOC domain of UBR4 was excised from the endogenous *UBR4* locus (UBR4 was already fused to a FLAG epitope) by CRISPR-Cas9 genome engineering. Similar approaches were used to eliminate the endogenous UBR- and calmodulin-binding regions in UBR4. Endogenous wildtype or mutant UBR4 was affinity-purified, and co-precipitating proteins were detected by Western blotting. Similar results in n = 3 independent experiments. **d**. Validation of mutant UBR4 by mass spectrometry. The KCMF1- or calmodulin-binding domains, or the UBR domain, were deleted in the endogenous locus of ^FLAG^*UBR4*. Endogenous UBR4 complexes were affinity-purified and bound proteins were detected by mass spectrometry. Changes in interactions for mutant cell lines compared to wildtype UBR4 are depicted for select proteins. Spectral counts were normalized to bait (UBR4) spectral counts in each cell line. For gel source data, see Supplementary Fig. 1.

Affinity purification of endogenous UBR4 showed abundant interactions with the E3 ligase KCMF1 (Fig. 1e, Extended Data Fig. 2a and Supplementary Table 2), as previously described^18,19^. UBR4 also bound calmodulin, the E2 enzyme UBE2A and—despite its cytosolic localization—several proteins that function in mitochondria. Reciprocal immunoprecipitation of endogenous KCMF1 confirmed its binding to UBR4, calmodulin, UBE2A and mitochondrial proteins (Fig. 1e and Extended Data Fig. 2a). Deletion of *KCMF1*, endogenous excision of a DOC domain in *UBR4* required for KCMF1 recruitment (Extended Data Fig. 2b,c) or deletion of the calmodulin-binding region in *UBR4* resulted in the same synthetic lethality as loss of *UBR4*, whereas the namesake UBR domain was not required (Fig. 1f and Extended Data Fig. 2d). We concluded that an E3 ligase complex that contains UBR4, KCMF1 and calmodulin sustains the survival of cells undergoing mitochondrial import stress. As explained below, we refer to this E3 ligase as SIFI.

## SIFI targets DELE1 and HRI

**Fig. 2:**
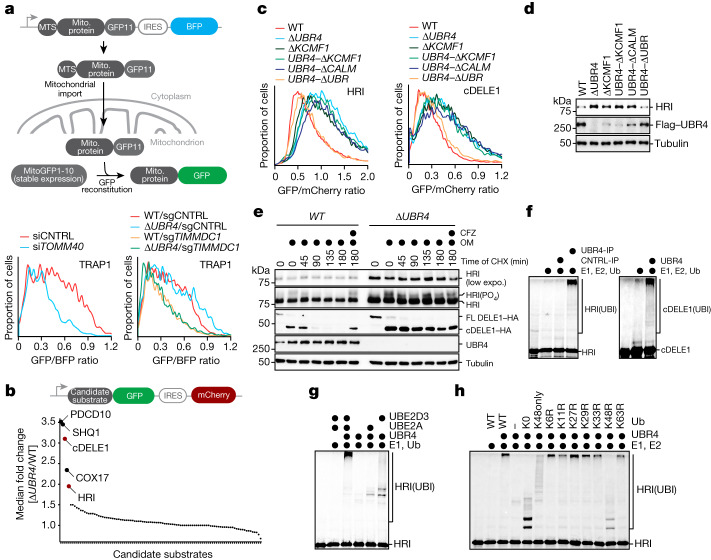
SIFI targets DELE1 and HRI. **a**, Top, outline of the import assay. Bottom, import assay using the model protein TRAP1 in WT or Δ*UBR4* cells lacking TOMM40 (left) or the UBR4 genetic interactor TIMMDC1 (right). Similar results in *n* = 3 independent experiments. Mito., mitochondrial; si, small interfering RNA **b**, Stability reporter-based screen for UBR4 substrates identifies cleaved DELE1 and HRI. Upper schematic: map of reporter construct with GFP-tagged candidate substrate co-expressed with mCherry under control of an internal ribosome entry site (IRES). **c**, cDELE1 or HRI stability were monitored by flow cytometry (*UBR4–*Δ*KCMF1*: KCMF1-binding domain deleted in endogenous *UBR4*; same for the other domains). Similar results in *n* = 2 independent experiments. **d**, Endogenous HRI increases in cells lacking SIFI, as measured by western blotting. Similar results in *n* = 3 independent experiments. **e**, WT or Δ*UBR4* cells that expressed endogenously tagged DELE1–HA were exposed to oligomycin (OM, 1 μM) before cycloheximide (CHX) and analysed by western blotting. Where indicated, carfilzomib (CFZ) was added. Similar results in *n* = 2 independent experiments. expo., exposure; FL, full length. **f**, ^35^S-labelled cDELE1(142–515)–SUMO or HRI(1–138)–SUMO were ubiquitylated by SIFI, E1, UBE2A, UBE2D3 and ubiquitin (Ub). Similar results in *n* = 3 independent experiments. IP, immunoprecipitation. **g**, SIFI-dependent ubiquitylation requires UBE2A and UBE2D3. Similar results in *n* = 2 independent experiments. **h**, SIFI assembles predominantly K48-linked ubiquitin chains. (K0, all Lys mutated; K48only, only Lys48 present; K48R, only Lys48 mutated). Similar results in *n* = 2 independent experiments. For gel source data, see Supplementary Fig. 1.

**Extended Data Fig. 3:**
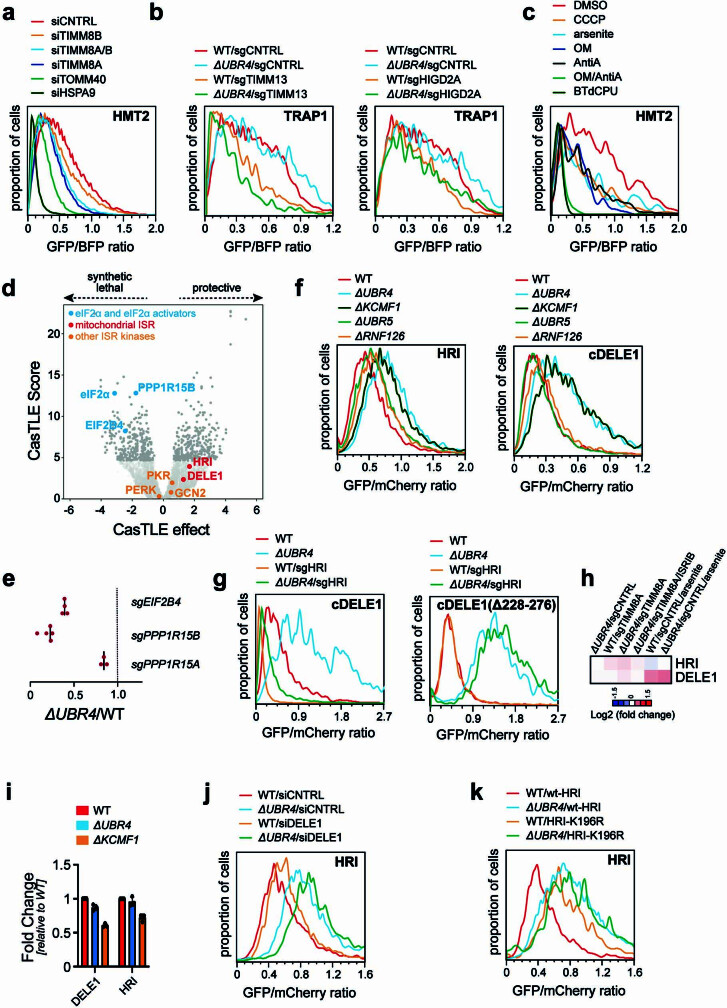
The SIFI complex targets cleaved DELE1 and HRI. **a**. Genetic interactors of UBR4 control mitochondrial protein import. Mitochondrial import of GFP11-tagged HMT2 was monitored in WT cells stably expressing mitochondrially targeted GFP(1–10). Genetic interactors of UBR4 or known protein import regulators were depleted with specific siRNAs. Similar results in n = 2 independent experiments. **b**. UBR4 does not regulate mitochondrial protein import. Import of GFP11-tagged TRAP1 was analyzed as above. When indicated, *ΔUBR4* cells were used or the genetic interactors of UBR4, TIMM13 and HIGD2A, were depleted using sgRNAs. Similar results in n = 3 independent experiments. **c**. Chemical stressors that deplete *ΔUBR4* cells in competition assays, compromised mitochondrial protein import. Import of GFP11-tagged HMT2 was analyzed in the presence of indicated drugs CCCP(5 μM), arsenite (10 μM), OM (2.5 μM), Antimycin A (10 μM), BTdCPU (10 μM) for 16 h by flow cytometry, as described above. Similar results in n = 2 independent experiments. **d**. Depletion of eIF2α, the eIF2B subunit EIF2B4, or the eIF2α phosphatase PPP1R15B causes synthetic lethality with loss of *UBR4*, as seen in our synthetic lethality screen described earlier. **e**. Validation of synthetic lethality between EIF2B4 and PPP1R15B (CReP) by cell competition assays. The second eIF2α phosphatase PPP1R15A (GADD34) also shows weak synthetic lethality with *UBR4* deletion. **f**. HRI and cleaved DELE1 are degraded through UBR4 and KCMF1, while the quality control E3 ligases UBR5 or RNF126 are not required. E3 ligases were deleted from 293 T cells by CRISPR/Cas9-mediated genome engineering and the stability of HRI or cDELE1 was monitored as GFP-tagged proteins by flow cytometry. Experiment performed once, similar results obtained with siRNA depletions. **g**. Degradation of orphan cDELE1, which is not bound to HRI, requires a central domain in cDELE1. A stability reporter expressing either cDELE1 or an internal deletion resistant to HRI depletion (cDELE1^Δ228–276^) were monitored by flow cytometry in either wildtype or *ΔUBR4* cells. When indicated, HRI was depleted by specific sgRNAs. Similar results in n = 2 independent experiments. **h**. Expression of HRI or DELE1 is not induced by mitochondrial stress, as seen by RNAseq in cells depleted of TIMM8A or treated with arsenite. Data was taken from RNAseq experiments described in Fig. 3d. **i**. Expression of HRI or DELE1 is not induced by deletion of *UBR4* or *KCMF1*, as seen by qRT-PCR. Graph shows mean ± SD of 3 independent experiments. **j**. Degradation of an overexpressed wildtype HRI reporter does not require DELE1. Stability of the HRI reporter was monitored in cells treated with control siRNAs or siRNAs targeting DELE1, by flow cytometry. Experiment performed once. **k**. Mutation of K196 in HRI, which is required for autophosphorylation and activation, prevents UBR4-dependent degradation, as seen by flow cytometry. Similar results in n = 3 independent experiments.

To determine whether SIFI regulates mitochondrial import, we used flow cytometry to monitor reconstitution of GFP after protein delivery to the mitochondrial matrix^20^. Similar to depletion of the channel subunit TOMM40 or the mitochondrial chaperone HSPA9, loss of the UBR4 genetic interactors TIMM8A, TIMM8B, TIMM13, TIMMDC1 or HIGD2A inhibited mitochondrial import (Fig. 2a and Extended Data Fig. 3a,b), as did chemical stressors that depleted ΔUBR4 cells in competition experiments (Extended Data Fig. 3c). However, *UBR4* deletion did not affect this process (Fig. 2a and Extended Data Fig. 3b), which suggested that SIFI does not target factors that mediate protein transport into mitochondria.

We therefore used protein stability reporters, as previously described^21,22^, to initiate an unbiased search for SIFI substrates. cDELE1, a sensor of mitochondrial import stress, and HRI, a kinase involved in the integrated stress response (ISR), were targeted by SIFI (Fig. 2b). When cells experience mitochondrial import stress, delayed translocation of DELE1 leads to cleavage by the protease OMA1 and release of a DELE1 fragment (cDELE1) into the cytoplasm. There, cDELE1 activates HRI to phosphorylate eIF2α and inhibit the translation initiation factor eIF2 (refs. ^23–25^), which can be reversed by the phosphatases PPP1R15A (also known as GADD34) or PPP1R15B (also known as CReP)^26,27^. Linking this pathway to SIFI function, loss of eIF2α, the eIF2 guanine nucleotide exchange factor subunit EIF2B4 or CReP showed synthetic lethality with *UBR4* deletion in our screen (Extended Data Fig. 3d). These results were confirmed in cell competition assays (Extended Data Fig. 3e). Notably, mutations in subunits of EIF2B cause leukoencephalopathy with vanishing white matter, another disease that shows symptoms reminiscent to those of patients with *UBR4* mutations^28^.

cDELE1 and HRI were also stabilized by loss of *KCMF1* or deletion of the KCMF1-binding and calmodulin-binding domains in UBR4, whereas the UBR domain in UBR4 or related quality control E3 ligases were not required (Fig. 2c and Extended Data Fig. 3f). We noted that cDELE1 was lost after depletion of its binding partner HRI. However, deletion of a central domain in DELE1 stabilized the orphan population and thus highlighted the strong contribution of UBR4 to cDELE1 turnover (Extended Data Fig. 3g). Western blotting showed that the levels of endogenous HRI increased after loss of UBR4, KCMF1 or the KCMF1-binding and calmodulin-binding domains in UBR4 (Fig. 2d). By contrast, mRNA levels of *HRI* or *DELE1* were not strongly affected (Extended Data Fig. 3h,i).

As overexpression activates HRI independently of DELE1 (ref. ^29^), the HRI reporter was degraded even if DELE1 had been depleted (Extended Data Fig. 3j). However, a HRI(K196R) variant that cannot be activated through autophosphorylation^30^ was protected against SIFI-dependent degradation (Extended Data Fig. 3k), and *UBR4* deletion selectively delayed the turnover of phosphorylated and active endogenous HRI (Fig. 2e). Similarly, *UBR4* deletion strongly stabilized the cDELE1 population that is produced during stress^23–25^ (Fig. 2e). These findings suggest that SIFI preferentially targets active HRI and cDELE1.

**Extended Data Fig. 4:**
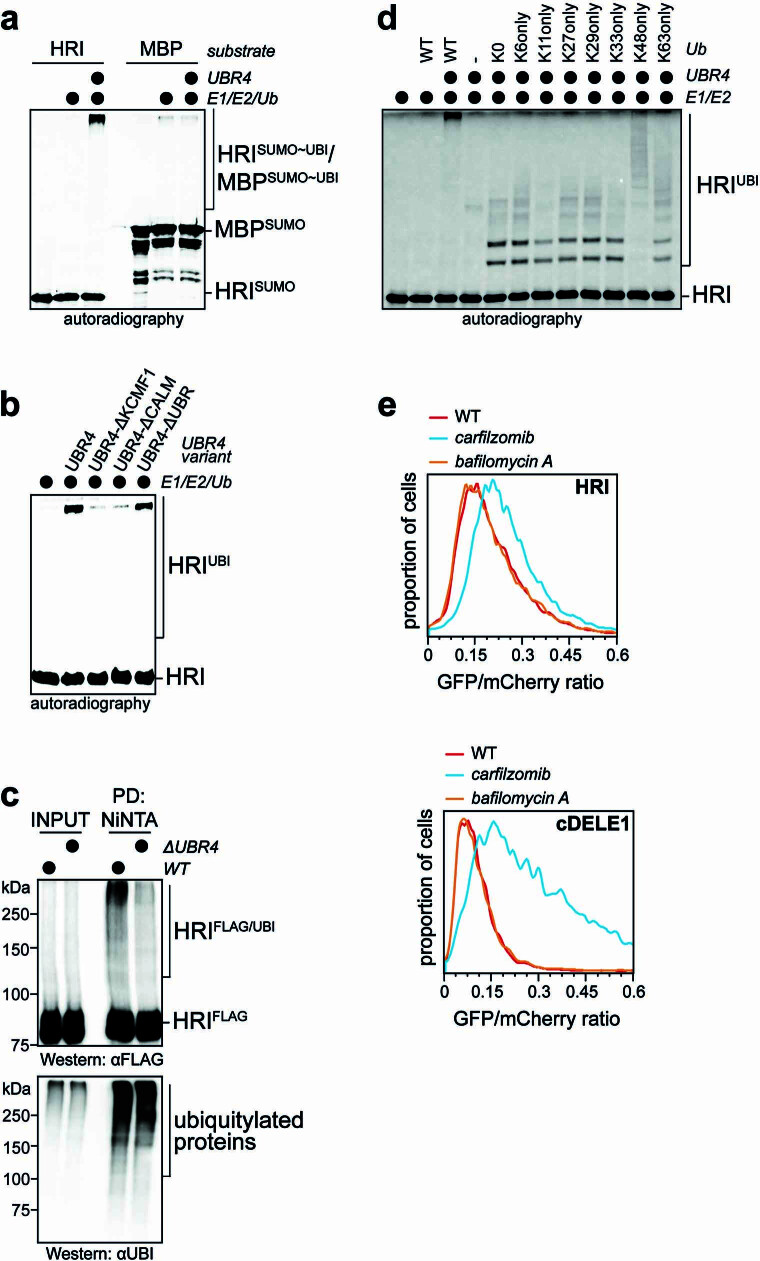
The SIFI complex targets HRI and cDELE1 for proteasomal degradation. **a**. HRI is ubiquitylated by the SIFI complex. The SIFI complex was purified from 293 T cells expressing endogenously FLAG-tagged UBR4. It was incubated with ^35^S-labeled HRI^1–138^-SUMO or MBP-SUMO as a control, respectively, E1, UBE2D3 and UBE2A as E2 enzymes, and ubiquitin. Reaction products were visualized by autoradiography. Experiment performed once. **b**. All SIFI subunits are required for HRI ubiquitylation. SIFI complexes were purified from cells expressing endogenously FLAG-tagged UBR4. When indicated, cells with internal deletions of the KCMF1-binding domain, the calmodulin-binding domain, or the UBR domain in endogenous *UBR4* were used. ^35^S-labeled HRI^1–138^-SUMO, E1, UBE2D3, UBE2A and ubiquitin were added, and reaction products were visualized by autoradiography. Similar results in n = 2 independent experiments. **c**. The SIFI complex mediates HRI ubiquitylation in cells. Ubiquitin conjugates were purified under denaturing conditions from cells expressing HRI^FLAG^ and ^HIS^ubiquitin, and modified HRI was detected by αFLAG Western blotting. Cells were treated with proteasome inhibitor (CFZ, 2 μM) for 6 h prior to harvesting. Similar results in n = 2 independent experiments. **d**. The SIFI complex modifies HRI with ubiquitin chains predominantly linked to K48 of ubiquitin. Ubiquitylation of ^35^S-labeled HRI^1–138^-SUMO was analyzed as described above, but in the presence of indicated ubiquitin mutants (K0: all Lys residues mutated to Arg; K6only: all Lys residues except for K6 mutated to Arg). Experiment performed once. **e**. HRI and cDELE1 are degraded through the proteasome. Cells were analyzed for levels of stability reporters encoding HRI-GFP or cDELE1-GFP by flow cytometry. The proteasome inhibitor carfilzomib (2 μM) or the lysosome inhibitor bafilomycin A (700 nM) were added for 6 h as indicated. Similar results in n = 2 independent experiments. For gel source data, see Supplementary Fig. 1.

Both cDELE1 and HRI were ubiquitylated by SIFI in vitro (Fig. 2f and Extended Data Fig. 4a), which required the E2 enzymes UBE2A and UBE2D3 and the KCMF1-binding and calmodulin-binding domains of UBR4 (Fig. 2g and Extended Data Fig. 4b). In addition, *UBR4* deletion impaired the ubiquitylation of HRI in cells (Extended Data Fig. 4c). SIFI modified HRI with predominantly K48-linked ubiquitin chains that are recognized by the proteasome (Fig. 2h and Extended Data Fig. 4d). Moreover, cDELE1 and HRI were stabilized by inhibitors of the proteasome, but not the lysosome (Extended Data Fig. 4e). We conclude that SIFI promotes the ubiquitylation and degradation of cDELE1 and HRI, proteins that actively mediate the cellular response to mitochondrial import stress.

## Stress response silencing by SIFI

**Fig. 3:**
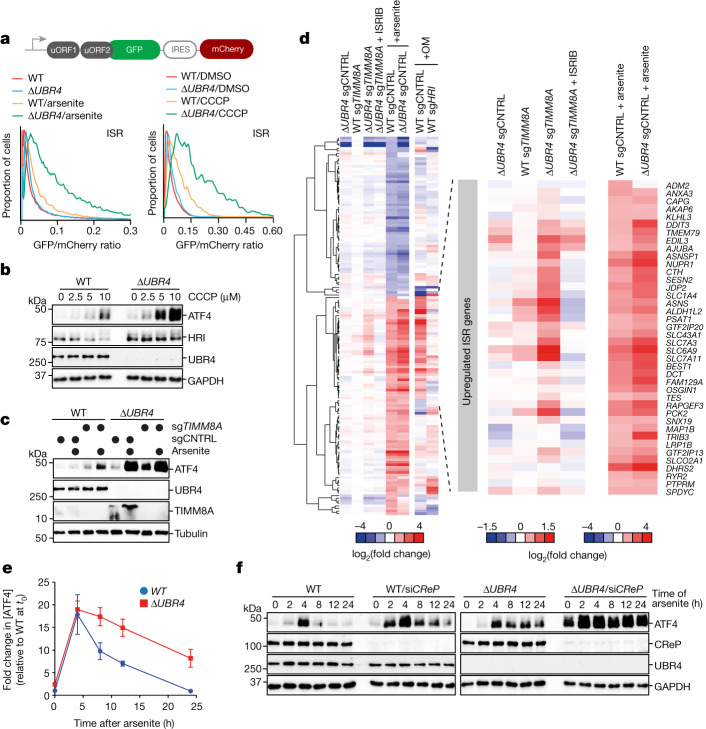
SIFI silences the mitochondrial stress response. **a**, *UBR4* deletion amplifies ISR signalling after arsenite (5 μM, 16 h) or CCCP (10 μM, 8 h) treatment, as detected by flow cytometry of uORF-ATF4-gated GFP translation. Upper schematic: map of ISR activation reporter, which measures uORF-gated translation of GFP controlled by IRES-mCherry. Similar results in *n* ≥ 2 independent experiments. **b**, WT and Δ*UBR4* cells were treated with CCCP (16 h) and ATF4 was detected by western blotting. Similar results in *n* = 2 independent experiments. **c**, Western blot of WT and Δ*UBR4* cells depleted of TIMM8A treated with arsenite (5 μM, 16 h). Similar results in *n* = 2 independent experiments. **d**, RNA-seq analysis of WT, Δ*UBR4*, WT sg*TIMM8A,* Δ*UBR4* sg*TIMM8A* and arsenite-treated WT and Δ*UBR4* cells. **e**, WT and Δ*UBR4* cells were treated with arsenite (5 μM), and ATF4 was monitored by western blotting. Quantification of *n* = 4 independent experiments. Data shown as the mean ± s.e.m. **f**, WT and Δ*UBR4* cells were depleted of CReP, treated with arsenite (5 μM) and analysed by western blotting. Similar results in *n* = 4 independent experiments. For gel source data, see Supplementary Fig. 1.

**Extended Data Fig. 5:**
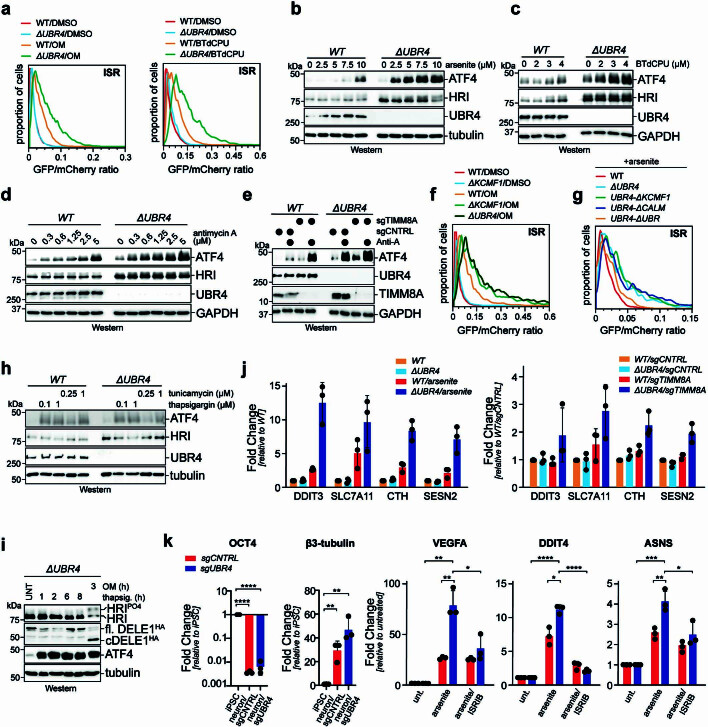
The SIFI complex silences the integrated stress response. **a**. Deletion of *UBR4* increases ISR signaling in response to cells being treated with oligomycin (0.2 μM) for 16 h or BTdCPU (7.5 μM) for 8 h. ISR activation was monitored by flow cytometry using the uORF-ATF4 reporter described above. Similar results in n = 2 independent experiments. **b**. *UBR4* deletion increases ISR signaling. Wildtype or *ΔUBR4* cells were treated for 16 h with increasing concentrations of arsenite and analyzed for ATF4 levels by Western blotting. Similar results in n = 2 independent experiments. **c**. Deletion of *UBR4* increases ISR signaling in cells treated for 16 h with increasing concentrations of BTdCPU, as monitored by Western blots detecting ATF4. Similar results in n = 2 independent experiments. **d**. *UBR4* deletion increases ISR signaling in cells treated for 16 h with increasing concentrations of antimycin A, as detected by ATF4 expression. Similar results in n = 2 independent experiments. **e**. Deletion of *TIMM8A* induces ATF4 accumulation more strongly in *ΔUBR4* cells. *WT* or *ΔUBR4* cells depleted of TIMM8A were treated with antimycin A (0.6 μM) for 16 h. Similar results in n = 2 independent experiments. **f**. Deletion of *KCMF1* increases ISR signaling to a similar extent as *UBR4* deletion, as detected using the uORF-dependent ISR reporter in flow cytometry. Cells were treated with OM (0.2 μM) for 8 h. Similar results in n = 2 independent experiments **g**. Deletion of KCMF1- and calmodulin-binding domains in the endogenous *UBR4* locus increases ISR signaling in response to 5 μM sodium arsenite for 16 h, as determined by flow cytometry using the uORF-dependent ISR reporter. Similar results in n = 2 independent experiments **h**. UBR4 does not restrict ISR signaling in response to endoplasmic reticulum stress. Wildtype or *ΔUBR4* cells were treated with thapsigargin or tunicamycin for 8 h and analyzed for ATF4 levels by Western blotting. Experiment performed once. **i**. ER stress activation by thapsigargin does not induce DELE1 cleavage. Cells were treated with thapsigargin (1 μM) or oligomycin (1 μM) for the indicated times. Experiment performed once. **j**. *UBR4* deletion increases ISR signaling, as read out by ATF4 activation. Wildtype or *ΔUBR4* cells were either treated with 5 μM sodium arsenite (*left panel*) or depleted of TIMM8A (*right panel*) and expression of established ATF4 target genes was determined by qPCR. Graph shows mean ± SD of 3 independent experiments. **k**. *UBR4* depletion increases ISR signaling in neurons derived from induced pluripotent stem cells by NGN2 activation. Differentiation was ensured by qRT-PCR against OCT4 and β3-tubulin and ISR target gene expression was measured by qRT-PCR. As indicated, either 5 μM sodium arsenite or ISRIB were added. Graph shows mean ± SD of 3 independent experiments. Statistical significance was determined using a two-tailed Student’s t-test. *p < 0.05, **p < 0.01, ***p < 0.001 and ****p < 0.0001. Exact p-Values: OCT4: sgCNTRL p < 0.0001; sgUBR4 p < 0.0001. β3-tubulin: sgCNTRL p = 0.0046; sgUBR4 p = 0.0014. VEGFA: sgUBR4 arsenite vs. unt. p = 0.0011; sgUBR4 arsenite vs. sgCNTRL arsenite p = 0.0052; sgUBR4 arsenite/ISRIB vs. sgUBR4 arsenite p = 0.0242. DDIT4: sgUBR4 arsenite vs. unt. p < 0.0001; sgUBR4 arsenite vs. sgCNTRL arsenite p = 0.0143; sgUBR4 arsenite/ISRIB vs. sgUBR4 arsenite p < 0.0001. ASNS: sgUBR4 arsenite vs. unt. p = 0.0004; sgUBR4 arsenite vs. sgCNTRL arsenite p = 0.0089; sgUBR4 arsenite/ISRIB vs. sgUBR4 arsenite p = 0.0236. For gel source data, see Supplementary Fig. 1.

By monitoring the translation and abundance of the transcription factor ATF4, which is induced by HRI^1^, we found that SIFI does not prevent spurious ISR activation (Fig. 3a–c and Extended Data Fig. 5a–e). However, *UBR4* deletion strongly increased ATF4 induction in cells exposed to mitochondrial stressors or had the import factor TIMM8A deleted (Fig. 3a–c and Extended Data Fig. 5a–e). Similar observations were made in cells that lacked the KCMF1-binding or calmodulin-binding domains of UBR4 or were devoid of *KCMF1* entirely (Extended Data Fig. 5f,g). By contrast, *UBR4* deletion did not affect ISR signalling caused by endoplasmic reticulum stress (Extended Data Fig. 5h,i). Thus, SIFI only restricts stress response signalling after it had been induced by mitochondrial import defects.

**Extended Data Fig. 6:**
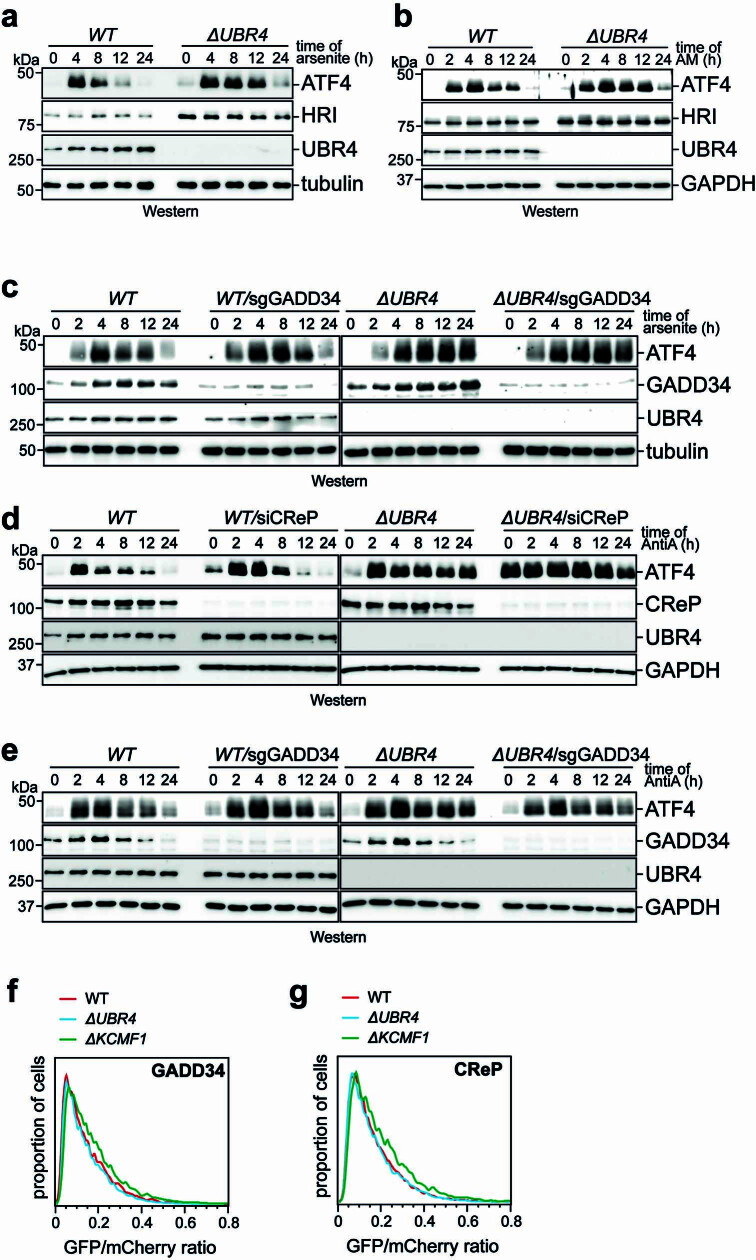
The SIFI complex silences the cellular response to mitochondrial import stress. **a**. The SIFI complex limits signal duration, not amplitude. Wildtype or *ΔUBR4* cells were treated with 5 μM sodium arsenite and cell lysates were analyzed for ATF4 levels by Western blotting over time. Quantification of 4 independent experiments shown in Fig. 3e. **b**. The SIFI complex also limits signal duration after ISR activation with 5 μM antimycin A (AM). Cell lysates were analyzed as described above. Similar results in n = 3 independent experiments. **c**. The SIFI complex, not the GADD34 phosphatase, mediates stress response silencing in response to arsenite. WT or *ΔUBR4* cells were depleted of GADD34, as indicated, and treated with 5 μM arsenite. At different times, samples were analyzed for ATF4 expression by Western blotting. Similar results in n = 2 independent experiments. **d**. The SIFI complex, not the CReP phosphatase, mediates stress response silencing after antimycin A treatment. WT or *ΔUBR4* cells were depleted of CReP, as indicated, and treated with 0.6 μM antimycin A. At different times, samples were analyzed for ATF4 expression by Western blotting. Similar results in n = 2 independent experiments. **e**. The SIFI complex, not the GADD34 phosphatase, mediates stress response silencing in response to antimycin A. WT or *ΔUBR4* cells were depleted of GADD34, as indicated, and treated with 0.6 μM antimycin A. At different times, samples were analyzed for ATF4 expression by Western blotting. Similar results in n = 2 independent experiments. **f**. The SIFI complex does not mediate degradation of GADD34, as shown by a GADD34 stability reporter in flow cytometry. Similar results in n = 2 independent experiments. **g**. The SIFI complex does not mediate degradation of CReP, as shown by a CReP stability reporter in flow cytometry. Similar results in n = 2 independent experiments. For gel source data, see Supplementary Fig. 1.

RNA sequencing (RNA-seq) and quantitative PCR with reverse transcription (RT–qPCR) analyses confirmed that SIFI limits ISR signalling after *TIMM8A* deletion or exposure of cells to mitochondrial stressors (Fig. 3d, Extended Data Fig. 5j and Supplementary Table 3). These results were also observed in neurons that mimicked the cell type affected in neurodegenerative disease (Extended Data Fig. 5k). To determine whether SIFI restricts the amplitude or duration of ISR signalling, we measured the time course of ATF4 induction in cells exposed to mitochondrial stress. In WT cells, ATF4 levels increased in response to stress but then declined to levels of untreated cells (Fig. 3e and Extended Data Fig. 6a,b). When Δ*UBR4* cells were treated with the same stressors, ATF4 peaked at similar levels but decreased much more slowly. These findings indicate that SIFI specifically acts to turn off stress response signalling.

The phosphatases CReP and GADD34 complement SIFI by reversing phosphorylation of the HRI target eIF2α^1,26,27^. Cells lacking GADD34 showed a mild delay in stress response silencing (Extended Data Fig. 6c). Conversely, CReP limited the extent of stress signalling, and its depletion frequently led to ISR activation by stresses encountered during growth in culture (Fig. 3f and Extended Data Fig. 6d). Cells lacking both CReP and SIFI could neither prevent spurious ISR activation nor turn off the stress response, which resulted in substantial ATF4 accumulation (Fig. 3f and Extended Data Fig. 6c–e). SIFI did not affect the stability of eIF2α phosphatases (Extended Data Fig. 6f,g), which led us to conclude that SIFI specifically restricts signal duration and thus acts as a silencing factor of the ISR.

## SIFI targets mitochondrial presequences

**Fig. 4:**
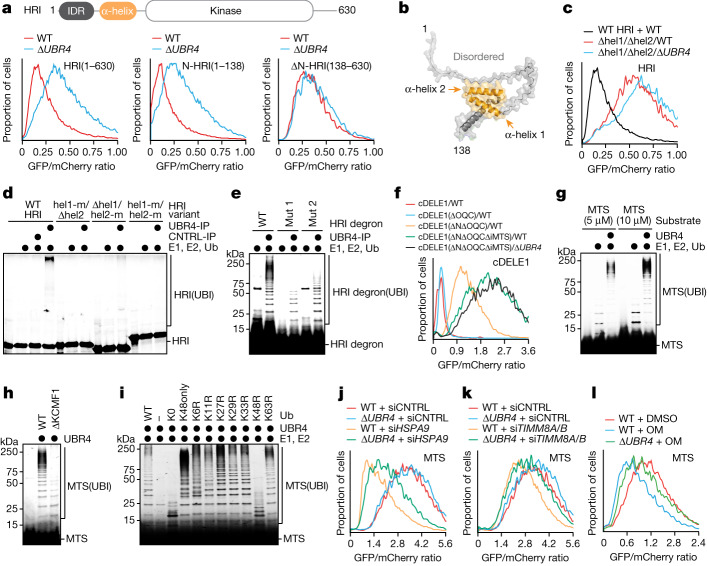
SIFI targets mitochondrial precursors. **a**, Stability of HRI variants in WT and Δ*UBR4* cells analysed by flow cytometry. Similar results in *n* ≥ 3 independent experiments. **b**, AlphaFold2 model of the amino-terminal HRI domain. **c**, Deletion of two helices protects HRI against UBR4-dependent degradation. Similar results in *n* = 3 independent experiments. **d**, Autoradiography image of HRI variants analysed for SIFI-dependent ubiquitylation. Similar results in *n* = 2 independent experiments. **e**, Fluorescence image of TAMRA-labelled HRI helix 2 peptides analysed for SIFI-dependent ubiquitylation. Similar results in *n* = 2 independent experiments. **f**, cDELE1 stability reporters were analysed in WT and Δ*UBR4* cells by flow cytometry. ΔN, amino-terminal deletion (152-end); ΔOQC, deletion of putative orphan QC motif; ΔiMTS, deletion of the region overlapping with the presequence-like helix. Similar results in *n* = 2 independent experiments. **g**, Ubiquitylation of a TAMRA-labelled presequence (MTS) peptide by SIFI, E1, UBE2A and UBE2D3 was monitored by fluorescence imaging. Similar results in *n* = 3 independent experiments. **h**, Fluorescence image of a TAMRA labelled presequence ubiquitylated by SIFI purified from WT and ΔKCMF1 cells. Experiment was performed once. **i**, Fluorescence image of modification of a TAMRA-labelled presequence with ubiquitin mutants. Similar results in *n* = 2 independent experiments. **j**–**l**, Flow cytometry results for MTS. **j**, Depletion of HSPA9 destabilizes a presequence reporter that is partially dependent on SIFI. Similar results in *n* = 2 independent experiments. **k**, Depletion of both TIMM8A and TIMM8B (si*TIMM8A/8B*) destabilizes a presequence–GFP fusion in a SIFI-dependent manner. Similar results in *n* = 2 independent experiments. **l**, Cells were treated with oligomycin (1 μM, 16 h), and the stability of a presequence–GFP fusion was determined by flow cytometry. Similar results in *n* = 2 independent experiments. For gel source data, see Supplementary Fig. 1.

**Extended Data Fig. 7:**
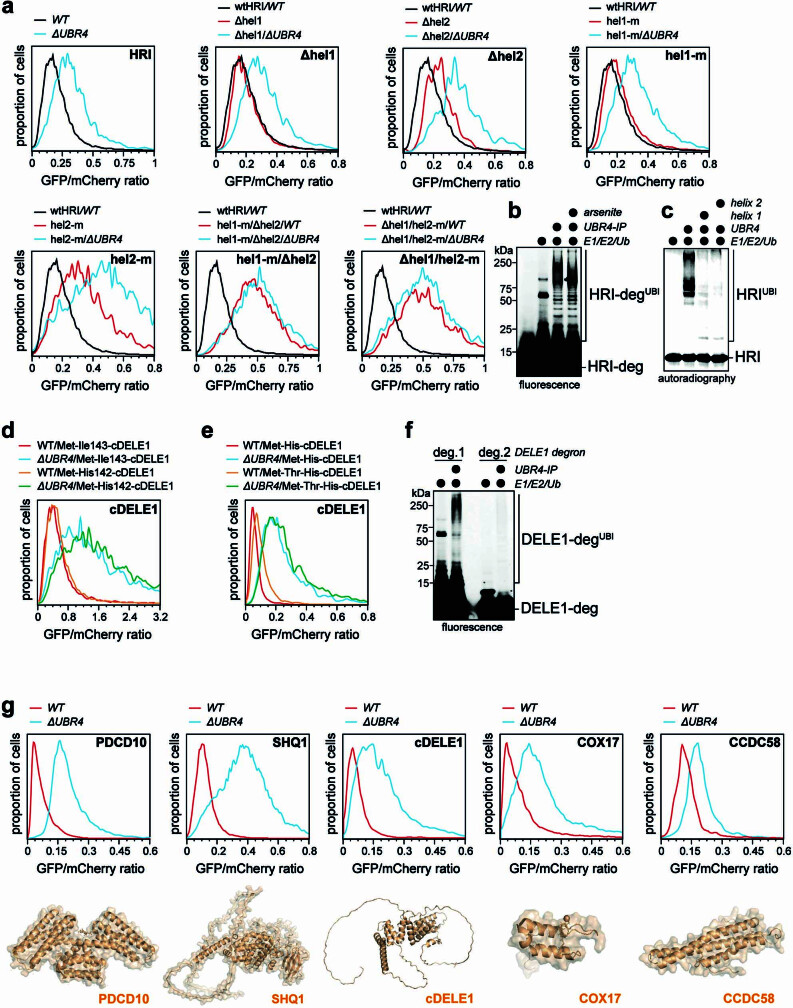
The SIFI complex detects helical degrons in HRI and DELE1. **a**. Deletion or mutation of two helices in HRI at the same time, but not manipulation of a single helix, protects HRI from UBR4-dependent degradation. The stability of indicated mutants was analyzed in wildtype or *ΔUBR4* cells by flow cytometry using the GFP/mCherry-based degradation reporter. Similar results in n ≥ 2 independent experiments. **b**. The SIFI complex ubiquitylates a single HRI peptide irrespectively of whether the SIFI complex was purified from control cells or cells treated with arsenite (40 μM for 4 h). Experiment performed once. **c**. Peptides encompassing a single HRI helix compete for ubiquitylation of the entire amino-terminal HRI domain (residues 1–138). ^35^S-labeled HRI^1–138^-SUMO was incubated with affinity-purified SIFI complexes, E1, UBE2A and UBE2D3, and ubiquitin. 200 μM of purified peptides encompassing the helices comprising degron 1 or degron 2, respectively, were added, and reaction products were analyzed by autoradiography. Similar results in n = 2 independent experiments **d**. Changing the amino-terminus of cleaved DELE1 does not affect its stability, as seen by flow cytometry. Similar results in n = 2 independent experiments **e**. Capping of the amino-terminus of cleaved DELE1 with threonine, an amino acid not recognized by the N-end rule, does not change its stability, as seen by flow cytometry. Similar results in n = 2 independent experiments. **f**. A helical DELE1 degron similar to HRI helices is ubiquitylated by the SIFI complex as a TAMRA-labeled peptide, while a distinct DELE1 peptide was not modified. Similar results in n = 2 independent experiments. **g**. Other top SIFI substrates are mostly composed of α-helices. The stability of top SIFI substrates identified in our screen was analyzed in wildtype or *ΔUBR4* cells by flow cytometry, using our degradation reporters. Similar results in n ≥ 2 independent experiments. AlphaFold2 models of each substrate are shown below. For gel source data, see Supplementary Fig. 1.

Deletion analyses showed that the amino-terminal domain of HRI was required and sufficient for SIFI-dependent degradation (Fig. 4a). AlphaFold2 modelling indicated that this domain contains two conserved α-helices (Fig. 4b), the deletion or mutation of which eliminated SIFI-mediated HRI ubiquitylation and degradation (Fig. 4c,d and Extended Data Fig. 7a). Conversely, a TAMRA-labelled HRI peptide could be ubiquitylated by SIFI in vitro (Fig. 4e and Extended Data Fig. 7b), and peptides encompassing each HRI helix were sufficient to prevent ubiquitylation of the entire amino-terminal HRI domain (Extended Data Fig. 7c). HRI therefore possesses two helices that each can mediate recognition by the E3 ligase SIFI.

Regarding cDELE1, deleting residues at the new amino terminus of the cleaved protein together with its orphan quality control motif impeded degradation (Fig. 4f and Extended Data Fig. 7d,e). An additional deletion that overlapped with a helix similar to the HRI degrons that was sufficient for SIFI-dependent ubiquitylation (Extended Data Fig. 7f) further stabilized cDELE1, and the triple mutant was now protected against degradation (Fig. 4f). Thus, SIFI recognizes multiple motifs in cDELE1, including an amino-terminal motif that is exposed after cleavage and a helix with similarity to HRI degrons. All other top SIFI substrates were rich in α-helices and might therefore be recognized through related mechanisms (Extended Data Fig. 7g).

**Extended Data Fig. 8:**
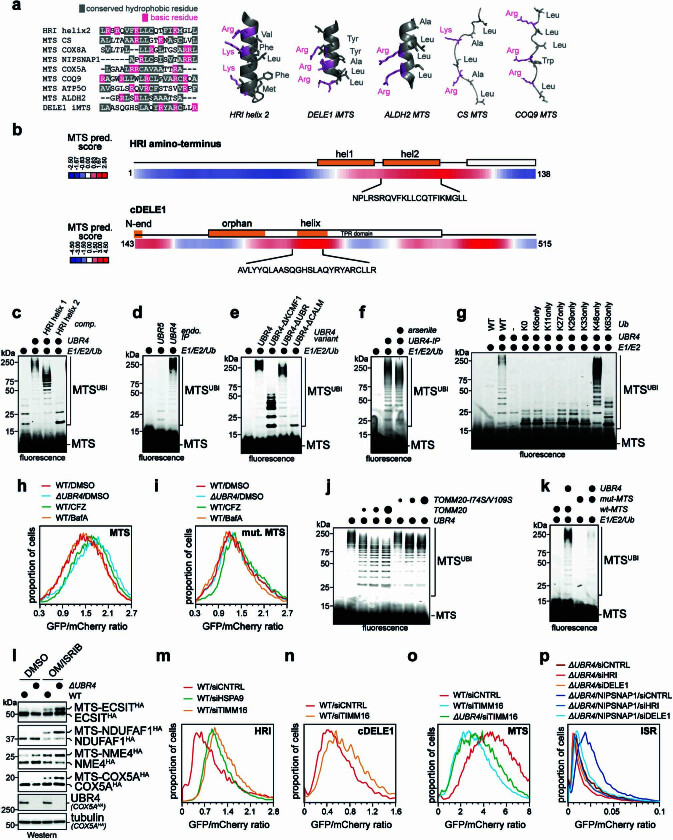
The SIFI complex recognizes mitochondrial presequences. **a**. Helical degrons in HRI and cDELE1 resemble mitochondrial presequences in amino acid composition (*left panel*) and structure (*right panels*). Presequences were aligned with COBALT (https://www.ncbi.nlm.nih.gov/tools/cobalt/re_cobalt.cgi). The structures of the HRI degron and the presequences of citrate synthase (CS) or COQ9 are AlphaFold2 models. The DELE1 helix is from its cryo-EM structure^56^ and the ALDH2 presequence is its actual structure when bound to TOMM20^57^. **b**. A prediction algorithm for mitochondrial presequences identifies the helical HRI and cDELE1 degrons. Internal MTS sequences were predicted using iMLP: iMTS-L predictor service (https://csb-imlp.bio.rptu.de/). A score above 0 is predictive of an internal MTS. Orange shaded boxes correspond to identified degrons in HRI and DELE1. **c**. The second HRI degron (helix 2) efficiently competes with mitochondrial presequences for access to the SIFI complex. A TAMRA-labeled COX8A presequence peptide (10 μM) was incubated with affinity-purified SIFI complex, E1, UBE2A and UBE2D3, and ubiquitin. As indicated, 100 μM of purified peptides encompassing the helices comprising degron 1 or degron 2 were added, and reaction products were analyzed after gel electrophoresis by fluorescence. Similar results in n = 2 independent experiments **d**. The SIFI complex, but not the quality control E3 ligase UBR5, ubiquitylates a presequence peptide. Ubiquitylation was analyzed as described above. Experiment performed once. **e**. The entire SIFI complex is required for presequence ubiquitylation. A TAMRA-labeled COX8A presequence peptide was incubated with affinity-purified SIFI complex purified from WT cells or cells carrying deletions of the endogenous KCMF1 binding-, calmodulin-, or UBR-domains in *UBR4*. E1, UBE2A and UBE2D3, and ubiquitin were added and reaction products were analyzed after gel electrophoresis by fluorescence. Similar results in n = 2 independent experiments. **f**. The SIFI complex ubiquitylates a TAMRA-labeled presequence peptide irrespectively of whether the E3 ligase had been purified from control cells or cells treated with arsenite (40 μM for 4 h). **g**. The SIFI complex modifies presequences with ubiquitin chains predominantly composed of K48-linkages. A TAMRA labeled COX8A presequence peptide was incubated with SIFI complex, E1, UBE2A and UBE2D3 and the indicated ubiquitin mutants (ubi-K0: all Lys residues mutated to Arg; ubi-K6only: all Lys residue except for K6 mutated to Arg), and reaction products were analyzed as above. Experiment performed once. **h**. The COX8A presequence is a SIFI-dependent degradation signal. The presequence was cloned as a fusion to GFP into the degradation reporter and assessed for its effects on protein stability by flow cytometry. As indicated, the proteasome inhibitor carfilzomib (CFZ) or the lysosome inhibitor bafilomycin A (BafA) were added. Note that only the cytoplasmic fraction of this fusion protein can be targeted via SIFI and the proteasome. Similar results in n = 2 independent experiments. **i**. A fusion between a COX8A presequence peptide carrying mutations in four Leu residues and GFP is not degraded through UBR4, the proteasome or the lysosome, as determined by flow cytometry. Similar results in n = 2 independent experiments. **j**. The mitochondrial import receptor TOMM20 competes with the SIFI complex for recognition of mitochondrial presequences. A TAMRA-labeled COX8A presequence was incubated with increasing concentrations of the cytoplasmic domain of TOMM20 or TOMM20^I74SV109S^, which is incapable of binding presequences. The SIFI complex, E1, E2s, and ubiquitin were added, and ubiquitylation was monitored by gel electrophoresis and fluorescence imaging. Experiment performed once. **k**. Mutation of presequence residues required for TOMM20 binding also ablates ubiquitylation by the SIFI complex. Similar results in n = 2 independent experiments. **l**. Import inhibition leads to accumulation of mitochondrial precursor proteins that still contain their presequence. *UBR4* deletion further increases precursor abundance, as seen by Western blotting after expressing of HA-tagged mitochondrial proteins in either WT or *ΔUBR4* cells treated with mitochondrial import blocker oligomycin (1 μM, 16 h) and ISRIB. Similar results in n = 2 independent experiments. **m**. Inhibition of mitochondrial protein import upon depletion of TIMM16 stabilizes HRI. Similar results in n = 2 independent experiments. **n**. Inhibition of mitochondrial protein import upon depletion of TIMM16 stabilizes cDELE1, as determined by flow cytometry. Similar results in n = 2 independent experiments. **o**. Deletion of *UBR4* partially stabilizes a presequence reporter if import was prevented by TIMM16 depletion, as seen by flow cytometry. Similar results in n = 2 independent experiments. **p**. Activation of ISR signaling in *ΔUBR4* cells upon overexpression of the mitochondrial protein NIPSNAP1 is dependent on HRI and DELE1. HRI and DELE1 were depleted by specific siRNAs and ISR activation was monitored using the uORF-ATF4 reporter using flow cytometry. When indicated, NIPSNAP1 was overexpressed. Similar results in n = 2 independent experiments. For gel source data, see Supplementary Fig. 1.

Notably, the helical HRI and cDELE1 degrons closely resembled mitochondrial presequences that mediate protein transport into the organelle (Extended Data Fig. 8a,b). These motifs accumulate in the cytoplasm when import is compromised, which raised the possibility that SIFI also recognizes unimported mitochondrial proteins that are known to be aggregation-prone^31,32^. Indeed, SIFI ubiquitylated a TAMRA-labelled presequence (Fig. 4g), which it engaged through the same site as the HRI degron (Extended Data Fig. 8c). By contrast, the E3 ligase UBR5, which recognizes distinct aggregation-prone proteins^10,33^, did not ubiquitylate presequences (Extended Data Fig. 8d).

The ubiquitylation of presequences depended on the calmodulin and KCMF1 subunits of SIFI (Fig. 4h and Extended Data Fig. 8e). Low-molecular-weight conjugates formed by KCMF1-deficient SIFI indicated that UBR4 initiates chain formation, whereas KCMF1 elongated or caused branching of conjugates (Extended Data Fig. 8e). Presequences were ubiquitylated with similar efficiency after chemical stress response activation (Extended Data Fig. 8f), and they were modified with predominantly K48-linked conjugates (Fig. 4i and Extended Data Fig. 8g). Accordingly, a presequence-containing protein was degraded by UBR4 and the proteasome (Extended Data Fig. 8h).

Competition, ubiquitylation and degradation experiments showed that SIFI recognizes presequences in a similar manner to that of the import machinery (Extended Data Fig. 8h–k). To test whether degradation of a presequence-containing protein was coupled to localization, we blocked import by depleting TIMM8A, TIMM8B or HSPA9. Both conditions strongly destabilized the presequence-containing protein, as it accumulated in the cytoplasm, and UBR4 was partially responsible for its clearance (Fig. 4j,k). Similar UBR4-dependent destabilization of a presequence-containing protein was observed when treating cells with chemical mitochondrial stressors (Fig. 4l). In cells with compromised import, deletion of *UBR4* also increased the abundance of presequence-containing mitochondrial precursors (Extended Data Fig. 8l). SIFI therefore not only degrades cDELE1 and HRI but it also targets unimported mitochondrial proteins that accumulate in the cytoplasm during import stress.

## Converging degrons time ISR silencing

**Fig. 5:**
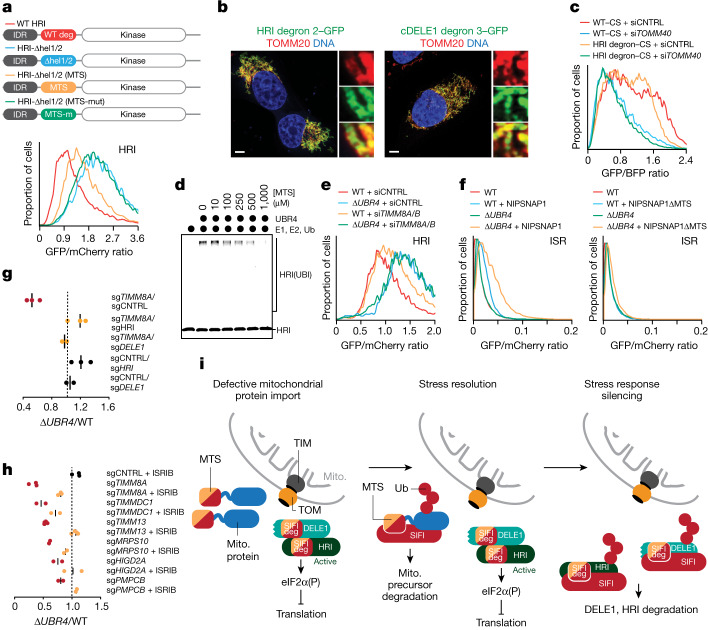
Converging degrons couple stress resolution to stress response silencing and cell survival. **a**, Degradation of WT-swap or degron/MTS-swap HRI reporters was monitored by flow cytometry. Similar results in *n* = 2 independent experiments **b**, The HRI helix 2 and the helical cDELE1 degron were fused in front of GFP, and localization was monitored by microscopy. Scale bar, 5 μm. Similar results in *n* = 3 independent experiments. **c**, The HRI helix 2 degron mediates import of citrate synthase (CS), as determined by flow cytometry. Experiment performed once and validated by microscopy. **d**, Autoradiography image of SIFI-dependent ubiquitylation of HRI(1–138)–SUMO in the presence of increasing concentration of presequence. Similar results in *n* = 2 independent experiments. **e**, Flow cytometry analysis of HRI stability in WT and Δ*UBR4* cells depleted of TIMM8A and TIMM8B. Similar results in *n* = 2 independent experiments. **f**, Overexpression of mitochondrial precursors induces ISR signalling that depends on presequences and restricted by UBR4, as monitored through the uORF-ATF4 reporter. Similar results in *n* = 2 independent experiments. **g**, DELE1 or HRI depletion rescues synthetic lethality after loss of *UBR4* and *TIMM8A*. **h**, ISRIB rescues the synthetic lethality after loss of *UBR4* and mitochondrial import or ETC assembly factors. Some competitions were performed at the same time as experiments for Fig. 1c and some controls are therefore reshown. **i**, Model of regulated stress response silencing by the E3 ligase SIFI. For gel source data, see Supplementary Fig. 1.

To probe the similarity between presequences and stress response degrons, we asked whether these motifs could complement each other. Deletion of both degrons in HRI led to the expected stabilization process (Fig. 5a). Insertion of a COX8A presequence into the original degron position revealed that even a single presequence could restore HRI degradation (Fig. 5a). A mutant presequence that is not recognized by the import machinery (Extended Data Fig. 8i,k) was unable to rescue HRI degradation in cells (Fig. 5a). Presequences that evolved to determine mitochondrial localization can therefore act as degrons within a stress response kinase activated by mitochondrial import defects.

Complementing these results, fusing helical HRI and cDELE1 degrons to GFP was sufficient to direct the hybrid proteins to mitochondria (Fig. 5b). To assess the efficiency of mitochondrial targeting by degrons, we exchanged the presequence of citrate synthase, a highly efficient import cargo^34^, with the HRI degron and monitored import by flow cytometry. This approach revealed that the HRI degron was as potent in promoting import as the physiological presequence (Fig. 5c). Similar to presequences, stress response degrons can therefore be recognized by both the import machinery and SIFI. We conclude that mitochondrial presequences and cDELE1 and HRI degrons are related bifunctional motifs that equally encode protein localization and stability. As these motifs probably emerged in response to different evolutionary pressures, we refer to such elements as ‘converging degrons’.

Our discovery of converging degrons raised the possibility that mitochondrial precursors compete with cDELE1 or HRI for access to SIFI to delay stress response silencing until import has been corrected. Indeed, a presequence peptide inhibited the SIFI-dependent ubiquitylation of HRI in a dose-dependent manner (Fig. 5d). Moreover, increasing the cytoplasmic levels of precursors by impairing import protected DELE1 and HRI against degradation in cells (Fig. 5e and Extended Data Fig. 8m–o). Overexpression of mitochondrial import cargo accordingly induced the ISR dependent on a presequence, DELE1 and HRI, but restricted by UBR4 (Fig. 5f and Extended Data Fig. 8p). On the basis of these findings, we propose that unimported mitochondrial precursors divert SIFI from DELE1 and HRI to sustain ISR signalling until the stress event has been resolved. Converging degrons therefore couple stress resolution to stress response silencing.

## Key role of stress response silencing

**Extended Data Fig. 9:**
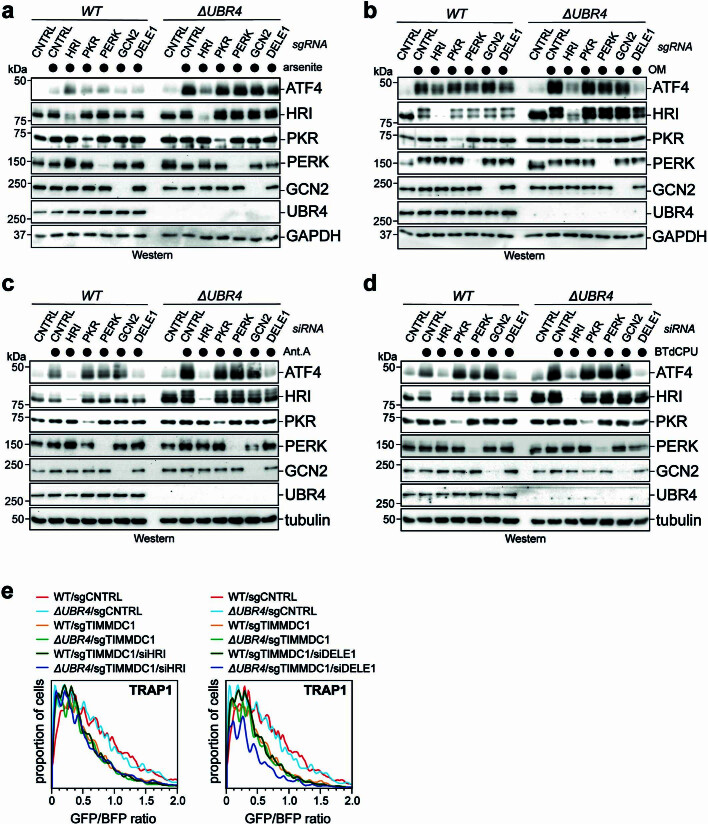
HRI and DELE1 mediate stress response signaling without affecting mitochondrial protein import. **a**. Depletion of HRI suppresses increased ISR activation in *ΔUBR4* cells treated with 5 μΜ sodium arsenite for 16 h, as monitored by Western blotting using antibodies against ATF4. Similar results in n = 2 independent experiments. **b**. Depletion of HRI or DELE1 suppresses increased ISR activation in *ΔUBR4* cells treated with 25 μΜ oligomycin for 8 h, as monitored by Western blotting using antibodies against ATF4. Similar results in n = 2 independent experiments. **c**. Depletion of HRI or DELE1 suppresses increased ISR activation in *ΔUBR4* cells treated with 0.6 μΜ antimycin A for 16 h, as monitored by Western blotting using antibodies against ATF4. Similar results in n = 2 independent experiments. **d**. Depletion of HRI or DELE1 suppresses increased ISR activation in *ΔUBR4* cells treated with 5 μΜ BTdCPU for 8 h, as monitored by Western blotting using antibodies against ATF4. Similar results in n = 2 independent experiments. **e**. Depletion of HRI and DELE1 by siRNA does not restore mitochondrial protein import in cells lacking TIMMDC1. Wildtype or *ΔUBR4* cells were depleted of TIMMDC1 using specific sgRNAs, as indicated. Mitochondrial protein import was monitored by reconstitution of GFP upon expression of TRAP1-GFP11 co-expressed with BFP in cells stably expressing GFP(1^–^10) in the mitochondrial matrix. GFP formation upon successful import was monitored by flow cytometry. Experiment was validated using the alternative mitochondrial import substrate HMT2-GFP11. For gel source data, see Supplementary Fig. 1.

As mutations in *UBR4* cause ataxia and early-onset dementia^13^, we wished to determine whether aggregation of mitochondrial precursors or prolonged stress signalling account for the deleterious consequences of *UBR4* deletion. Depletion of HRI or DELE1 in Δ*UBR4* cells was sufficient to reduce ISR signalling in response to chemical stressors (Extended Data Fig. 9a–d), whereas it did not affect mitochondrial import (Extended Data Fig. 9e). Notably, loss of HRI or DELE1 restored the proliferation of Δ*UBR4* cells subjected to mitochondrial import stress (Fig. 5g). This was a noteworthy result, as HRI inhibition releases the break on protein synthesis and thus increases the production of aggregation-prone proteins. Persistent stress response signalling, rather than accumulation of aggregation-prone proteins, therefore compromises the fitness of SIFI mutant cells.

**Extended Data Fig. 10:**
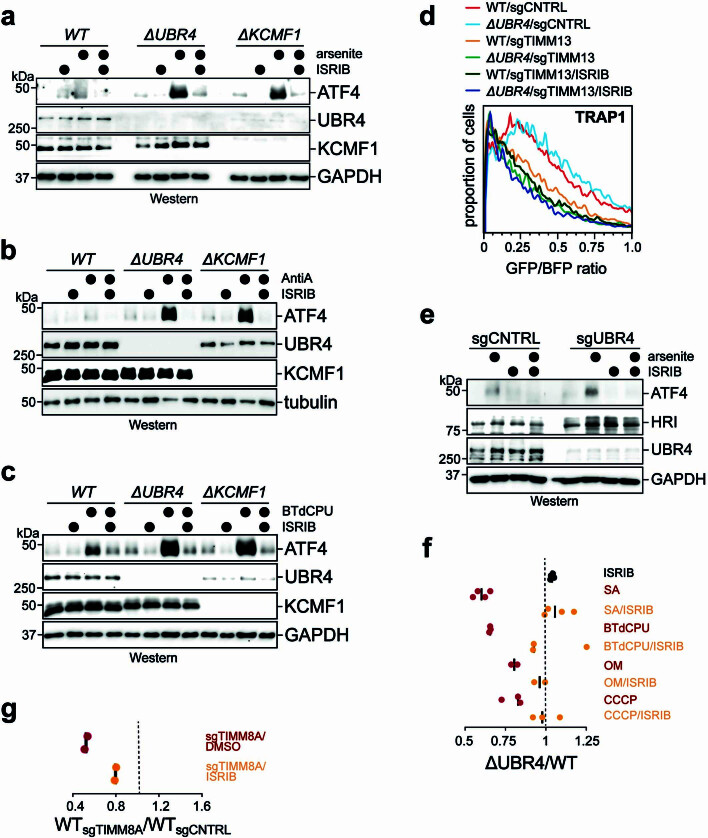
Stress response silencing restores cell survival. **a**. Pharmacological stress response silencing in cells lacking *UBR4* or *KCMF1* through ISRIB. Wildtype, *ΔUBR4*, or *ΔKCMF1* cells were treated with 5 μΜ sodium arsenite for 16 h and, as indicated, ISRIB. ATF4 levels were monitored by Western blotting. Similar results in n = 2 independent experiments. **b**. ISRIB inhibits stress response activation in cells that were lacking *UBR4* or *KCMF1* and were treated with antimycin A (0.6 μΜ) for 16 h. Similar results in n = 2 independent experiments. **c**. ISRIB inhibits stress response activation in cells that were lacking *UBR4* or *KCMF1* and were treated with BtdCPU (5 μΜ) for 8 h. Similar results in n = 2 independent experiments. **d**. ISRIB does not restore mitochondrial protein import in cells depleted of TIMM13. Import was measured upon GFP reconstitution by flow cytometry, as described above. Experiment was validated using the alternative mitochondrial import substrate HMT2-GFP11. **e**. ISRIB rescues ISR activation in human embryonic stem cells. As indicated, UBR4 was depleted by sgRNAs. Sodium arsenite (1.25 μΜ) and/or ISRIB were added for 8 h and ATF4 activation was monitored by Western blotting. Similar results in n = 2 independent experiments. **f**. Pharmacological silencing of the ISR with ISRIB rescues the synthetic lethality between *UBR4* deletion and chemical mitochondrial stressors. Cell competition assays were performed as described above. Some competitions were performed at the same time as for Fig. 1d and are therefore re-reshown from Fig. 1d. **g**. Pharmacological silencing of the ISR with ISRIB rescues the cells depleted of the disease gene *TIMM8A*. Cell competition assays were performed as described above. For gel source data, see Supplementary Fig. 1.

To corroborate these findings, we used the small-molecule compound ISRIB, which inactivates the ISR downstream of HRI^1,35^. ISRIB was sufficient to restrain ISR activation in Δ*UBR4* cells and in Δ*KCMF1* cells (Extended Data Fig. 10a–c) without correcting import defects (Extended Data Fig. 10d). ISRIB similarly blunted ISR signalling in *UBR4*-deficient stem cells or neurons (Extended Data Figs. 5k and 10e). Notably, ISRIB rescued Δ*UBR4* cells that were exposed to mitochondrial stressors or lacked import factors such as TIMM8A (mutated in Mohr–Tranebjærg syndrome) or PMPCB (deficient in childhood ataxia) (Fig. 5h and Extended Data Fig. 10f). Similar results were observed in WT cells that lacked TIMM8A and thus mimicked conditions in Mohr–Tranebjærg syndrome (Extended Data Fig. 10g). Pharmacological stress response silencing therefore restores cell survival even if aggregation-prone proteins cannot be cleared. As derivatives of ISRIB have entered clinical trials, these findings offer a path towards treating neurodegenerative diseases caused by mitochondrial protein import stress.

## Discussion

Our study demonstrated that stress response silencing is an active and regulated process that requires a dedicated silencing factor: the E3 ligase SIFI. SIFI targets unimported mitochondrial precursors, the sensor DELE1 and the stress response kinase HRI through motifs that equally encode protein trafficking and degradation. Stress response silencing therefore involves the monitoring of every step of the pathway through related sequence elements, referred to as converging degrons (Fig. 5i). As SIFI preferentially detects active HRI and cDELE1, we hypothesize that degrons are exposed by phosphorylation-dependent conformational changes in HRI^36^ and cleavage of DELE1 (refs. ^23–25^), respectively.

Because unimported precursors and stress response components possess similar degrons, competition for access to SIFI ensures that stress response silencing is delayed until mislocalized proteins have been cleared. In this manner, SIFI turns off the ISR after a specific stress (that is, mitochondrial import defects) has been addressed, even though HRI can also be activated by protein aggregation, haem depletion or pathogen infection^23,29,37^. The complex information encoded in converging degrons also enables SIFI to distinguish mitochondrial precursors from sequences that should not be targeted, such as positively charged microtubule-binding or nuclear localization signals. Converging degrons therefore endow cells with the capacity to accurately silence a broad stress response after a specific insult had been resolved. However, if unimported proteins cannot be cleared because of aggregation, continued competition of converging degrons for SIFI access results in persistent stress response signalling, with severe consequences for the cell.

Underscoring the importance of SIFI, mutation of *UBR4* or several genetic interactors causes neurodegenerative pathologies with overlapping symptoms^16,38,39^. Although mutant cells accumulate mislocalized proteins, which are known to be aggregation-prone^31,40^, we found that restoration of stress response silencing was sufficient to rescue their survival. We therefore propose that pathologies driven by persistent stress induction or delayed stress response inactivation, including early-onset dementia caused by *UBR4* mutations, could benefit from compound-induced stress response silencing. Notably, ISRIB restores memory formation in certain diseases^41^, which suggests that stress response silencing could boost both neuronal function and survival. As inactivation of a kinase-dependent stress response is probably more feasible than removal of large aggregates, it will be worth assessing whether pharmacological stress response silencing could similarly help patients with other protein aggregation diseases.

## Methods

### Data reporting

No statistical methods were used to predetermine sample sizes. The experiments were not randomized and the investigators were not blinded to allocation during experiments and outcome assessment.

### Mammalian cell culture

HEK293T and U2OS cells were maintained in DMEM + Glutamax (Gibco, 10566-016) and 10% FBS (VWR, 89510-186). All cell lines were purchased directly from the UC Berkeley Cell Culture Facility, authenticated by short tandem repeat analysis and were routinely tested for mycoplasma contamination using a Mycoplasma PCR Detection kit (abmGood, G238). All cell lines tested negative for mycoplasma. For growth in galactose, DMEM with no glucose (Gibco, 11966025) was supplemented with 20 mM galactose.

Plasmid transfections were performed using polyethylenimine (PEI; Polysciences 23966-1) at a 1:6 ratio of DNA (in μg) to PEI (in μl at a 1 mg ml^–1^ stock concentration) or Lipofectamine 3000 transfection reagent per the manufacturers’ instructions (Thermo Fisher, L3000008). siRNA transfections were performed using indicated siRNAs (at a final concentration of 20 nM) and 3 μl (12-well plate) or 6 μl (6-well plate) of RNAiMAX (Thermo Fisher, 13778150). siRNA sequences used in this study are available in Supplementary Table 6. Lentiviruses were produced in HEK293T cells by co-transfection of lentiviral and packaging plasmids using Lipofectamine 3000. Virus-containing supernatants were collected 48 h and 72 h after transfection, supernatants were spun down and concentrated using a Lenti-X concentrator (Takara, 631232), aliquoted and stored at −80 °C for later use. For lentiviral transduction, 10^5^ cells were seeded into 24-well plates and subjected to centrifugation for 45 min at 1,000*g* after addition of lentiviral particles and 6 μg ml^–1^ polybrene (Sigma-Aldrich, TR-1003). HEK293T transduced cells were drug-selected 24 h after infection with the following drug concentrations when applicable: puromycin (1 μg ml^–1^; Sigma-Aldrich, P8833); blasticidin (7.5 μg ml^–1^; Thermo Fisher, A1113903); or hygromycin (75 μg ml^–1^; Gibco, 10687010).

The following lentiviral constructs were used to infect human embryonic stem (ES) cells: (1) lentivirus vector pLG15_UBR4_GFP (sgUBR4) expressing GFP and the sgRNA sequence GGTCATCGAGAGGTACCGGG under the mU6 promoter; (2) lentivirus vector pLG15_NC766_mOrange (sgCNTRL) expressing mOrange and the control sgRNA sequence GGGTGATGCGGACAGGCCCG under the mU6 promoter. These lentiviruses were produced in HEK293T cells (American Type Culture Collection, CRL-3216) by co-transfection with three helper plasmids (pRSV-REV, pRRE and vesicular stomatitis virus G protein expression vector) using PEI. Lentiviral particles were then filtered through a 0.45 µm filter (EMD Millipore, SLFH05010), ultracentrifuged, resuspended in DMEM 100 times smaller than the original volume and stored at −80 °C. Human H1 ES cells were maintained in StemFlex medium (Thermo Fisher, A3349401) containing neomycin (final concentration of 300 µg ml^–1^; Thermo Fisher, 11811098) and hygromycin (final concentration of 50 µg ml^–1^; Sigma-Aldrich, H3274) on plates coated with Matrigel (Corning, 354234). Human H1 ES cells were used as the parental line for genetic engineering. ES cells were transfected with a piggybac vector with Ubc-dCas9-BFP-KRAB/EF1α-rtTA-T2A-hygromycin and a Super PiggyBac Transposase Expression vector (System BioSciences, PB210PA-1) by using Lipofectamine Stem Transfection reagent (Thermo Fisher, STEM00001). After 1 week of selection with 50 µg ml^–1^ hygromycin, BFP-positive ES cells were sorted by FACS and plated in a serial dilution series. Individual clones were picked under an inverted microscope in a tissue culture hood by manual scraping. Clones that were 100% BFP positive in flow cytometry analysis were selected and transfected with a piggybac vector with TetO-Ngn2/EF1a-rtTA-IRES-NEO and a Super PiggyBac Transposase Expression vector by using Lipofectamine Stem Transfection reagent. Cells selected by 300 µg ml^–1^ of neomycin for 2 weeks were used for further experiments.

To generate *UBR4* knockdown cells, cultures were briefly dissociated using accutase (Innovative Cell Technologies, AT104), replated at a density of 5 × 10^5^ cells per well in a 6-well plate on Matrigel in the presence of 10 µM of the ROCK inhibitor Y-27632 (Axon Medchem, 1683). At the same time as plating, lentivirus prepared as described above (3 µl per well of a 6-well plate) was added. The day after plating, medium was changed to StemFlex medium without Y-27632, and the following day, neomycin and hygromycin were reintroduced into the medium. For analysis of ISR activation, cells infected with either sgCNTRL or sg*UBR4* lentivirus were treated with either 0 µM or 5 µM sodium arsenite (Fisher Scientific, 7142-16) for 8 h both in the presence and absence of 200 nM ISRIB (Sigma Aldrich, SML0843). After treatment, cells were dissociated using accutase, washed 3× with PBS and pelleted by table-top centrifugation. Cell pellets were snap-frozen in liquid nitrogen and stored at −80 °C until western blot analysis.

For iNeurons experiments, induced pluripotent stem cells (iPS cells) harbouring doxycycline-inducible murine neurogenin-2 (*Ngn2*) and expressing dCas9–KRAB in the WTC-11 background (gift from M. Ward, NIH) were maintained in mTeSR plus (StemCell Technologies, 100-0276) on Matrigel-coated plates (Corning, 356231). Guide RNAs (NTC: GTGCACCCGGCTAGGACCGG; UBR4: GGGGAGCCGCAGTAGTACGA) were cloned into the pMK1334 vector (gift from M. Kampmann, Addgene, 127965) and introduced to iPS cells by lentiviral transduction. Neuronal differentiation was performed as previously described^42^. In brief, iPS cells were dissociated using accutase (StemCell Technologies, 07920) and replated on Matrigel-coated plates in N2 induction medium containing DMEM/F12 with Glutamax (Gibco, 10565018), 1× MEM NEAA (Gibco, 11140050), 1× N-2 supplement (Gibco, 17502048), doxycycline (2 μg ml^–1^) and Chroman I (50 nM; MedChem Express, HY-15392). N2 induction medium was changed daily, omitting Chroman I. After 48–72 h of exposure to doxycycline, pre-differentiated neurons were dissociated by accutase treatment and replated onto poly-l-ornithine-coated (Sigma Aldrich, P3655) 12-well plates at 5 × 10^5^ cells per well in neuronal maturation medium containing 50% BrainPhys (StemCell Technologies, 05790), 50% DMEM/F12 (Gibco, 11039021), 1× B-27 plus supplement (Gibco, A3582801), GDNF, BDNF, NT-3 (10 ng ml^–1^ each; PeproTech, 450-10, 450-02, 450-03), mouse laminin (1 μg ml^–1^; Gibco, 23017015), and doxycycline (2 μg ml^–1^). After 3 days, a full medium change was performed using neuronal maturation medium containing 100% BrainPhys without doxycycline. Drug treatments were conducted on day 7 after replating onto poly-l-ornithine-coated plates.

### Plasmids

The list of all constructs used in this study are provided in Supplementary Table 4. Most cloning was performed using Gibson assembly using HIFI DNA Assembly master mix (NEB, E2621L).

### Generation of CRISPR–cas9 genome edited cell lines

All CRISPR–cas9 edited cell lines used in this publication were generated from HEK293T cells. sgRNA sequences were designed using the online resource provided by IDT. DNA oligonucleotides for sgRNA and their complementary sequence were phosphorylated (NEB, M0201), annealed and ligated (NEB, M0202) into pX330. HEK293T cells were cultured in a 6-well plate and transfected at 50% confluence with 2 µg of px330 plasmids (and 1 μl of 10 μM single stranded donor oligonucleotide when applicable) using Mirus TransIT-293 Transfection reagent (Mirus, MIR2705). At 48 h after transfection, individual clones were expanded in 96-well plates. Homozygous clones were screened by PCR and DNA sequencing and confirmed by western blotting when applicable.

HEK293T Flag–UBR4 and Flag–UBR5 cells were generated as previously described^10^. For generation of Δ*UBR4* cells, two sgRNAs were used to remove exon 2 with protospacer sequences 5′-ggttgatgatactatctacc-3′ and 5′-ccttacctaggctaaccaag-3′. Δ*KCMF1* cells were generated in the Flag–UBR4 background, two sgRNAs were used to remove exon 3 with protospacer sequences 5′-tgtaatctcagctgctccgg-3′ and 5′-acggtatcattacactgagc-3′. For generation of KCMF1–Flag, we used the following sgRNA: 5′-gaattgggatgtcatcaaag-3′ and ssODN 5′-gctttagaaaacctaaatttaaaagagagtaataaaggaaatgagcctccaccacctcctcttggcgcgccagactacaaagaccatgacggtgattataaagatcatgatatcgattacaaggatgacgatgacaagtgatgacatcccaattcgcagacaatgtcctctgtgctgtatttgccaatgaaagtggacaa-3′.

UBR4-ΔKCMF1 (Δ2333–2498), UBR4-ΔUBR (Δ1653–1725), UBR4-ΔCALM (Δ4036–4131) were generated in the Flag–UBR4 background with the following protospacer sequences that created in-frame deletions: UBR4-ΔKCMF1: 5′-gggtttccaccaataccagc-3′ and 5′-ctgtgacacacgctcactat-3′; UBR4-ΔUBR: 5′-caagccaccctttatagctc-3′ and 5′-gttgactcgcaaatgacccg-3′; UBR4-ΔCALM: 5′-gagcgtgttaagataagcag-3′ and 5′-gagtgaccttaagctcaatg-3′.

Δ*UBR5* cells were generated as previously described^43^. For generation of Δ*RNF126* cells, the following sgRNAs were used to remove exon 2: 5′-gccctccaggacccacgggtt-3′ and 5′-gctcttccagcctcttcaac-3′.

DELE1–HA cells were generated using the following sgRNAs: 5′-gaaaggagtgttgtaagact-3′ and 5′-agtcttacaacactcctttc-3′ and ssODN 5′-ctattcccccacacccctacccactggaaaggagtgttgtaagactaggttttggctacccgtatgatgttccggattacgctggctacccatacgacgtcccagactacgctggctacccatacgacgtcccagactacgcttaaggtgagataaaacatagtccctggtgcctcttaggggccagagcgggcaggagg-3′.

### Synthetic lethal whole-genome CRISPR–Cas9 screen

We followed a CRISPR–Cas9 screening protocol as previously described^44^. In brief, pooled sgRNA viruses were obtained by transfection of the Human GeCKO v2 library (Addgene, 1000000048) into HEK293T cells together with lentiviral packaging plasmids using Mirus TransIT-293 Transfection reagent. HEK293T WT and Δ*UBR4* cells were spinfected with the pooled sgRNA virus at a multiplicity of infection of 0.3 with 8 μg ml^–1^ polybrene in 12-well plates. Cells were trypsinized and replated the next day onto 15-cm plates and selected with puromycin (1 μg ml^–1^) for 3 days, until the untransduced control cells were all dead. After puromycin selection, cells were split and seeded at a density of 2.5 × 10^6^ cells per 15-cm plate and this marked day 0. Cells were grown in DMEM + Glutamax with penicillin–streptomycin (Gibco, 15070063) and split every 3 days until day 21, the final day of the screen. Cells were cultured such that a representation of at least 500 cells per sgRNA element was maintained throughout the screen. A total of 70 × 10^6^ cells were collected at day 0 and day 21 for genomic DNA extraction, which was performed using a Zymo Research Quick-gDNA MidiPrep kits (Zymo Research, D3100) according to the manufacturer’s protocol. sgRNA-encoding regions were amplified with Q5 High-Fidelity DNA polymerase (NEB, M0491). All PCRs for a given sample were pooled, and 500 µl was used to perform ampure bead clean-up with 0.65× and 0.9× cut-off values (Beckman Coulter, A63881). Samples were run on a 8% TBE gel (Thermo Fisher, EC6215BOX), gel purified and sequenced at the UC Berkeley Vincent J. Coates Genomics Sequencing laboratory on a HiSeq4000. sgRNA counts were processed using count_spacers.py^44^. Subsequently, CasTLE^45^ was run to identify top candidate genes that were synthetic lethal or protective in Δ*UBR4* cells compared with WT cells. We used the non-expressed genes (as defined by having zero transcripts per million (TPM) in HEK293T WT cells by RNA-seq analysis, *n* = 4,710) as the negative control gene set instead of non-targeting control guides (sgNTCs) to run CasTLE. This allows for a much more representative background distribution because there are few sgNTCs in the lentiv2 library and they are known to introduce biases due to the absence of cutting^46^. To identify pathways enriched in the candidate genes, we took genes in the 5% top CasTLE score with a negative CasTLE Effect and ran Gene Ontology enrichment analysis (Cytoscape, ClueGO v.3.7.1). CasTLE effects and scores are available in Supplementary Table 1.

### Mass spectrometry

Mass spectrometry was performed on immunoprecipitates prepared from 40 15-cm plates of endogenously Flag-tagged UBR4 or KCMF1 HEK293T cell lines (Supplementary Table 2). Cells were lysed in lysis buffer (20 mM HEPES, pH 7.5, 150 mM NaCl, 0.2% Nonidet P-40, benzonase (Sigma-Aldrich, E1014), 1× complete protease inhibitor cocktail (Roche, 11836170001), 1× PMSF, 10 mM NaF and 1 mM sodium orthovanadate), lysed extracts were clarified by centrifugation at 21,000*g* and bound to anti-Flag M2 affinity resin (Sigma-Aldrich, A2220) for 2 h at 4 °C. Immunoprecipitates were then washed 4× and eluted 3× at 30 °C with 0.5 mg ml^–1^ of 3×Flag peptide (Sigma, F4799) buffered in 1× PBS plus 0.1% Triton X-100. Elutions were pooled and precipitated overnight at 4 °C with 20% trichloroacetic acid. Spun down pellets were washed 3× with an ice-cold acetone and 0.1 N HCl solution, dried, resolubilized in 8 M urea buffered in 100 mM Tris pH 8.5, reduced with TCEP, at a final concentration of 5 mM, (Sigma-Aldrich, C4706) for 20 min, alkylated with iodoacetamide, at a final concentration of 10 mM (Thermo Fisher, A39271) for 15 min, diluted 4-fold with 100 mM Tris pH 8.5, and digested with 0.5 mg ml^–1^ of trypsin (Promega, v5111) supplemented with CaCl_2_ (at a final concentration of 1 mM) overnight at 37 °C. Trypsin-digested samples were submitted to the Vincent J. Coates Proteomics/Mass Spectrometry Laboratory at UC Berkeley for analysis. Peptides were processed using multidimensional protein identification technology (MudPIT) and ran on a LTQ XL linear ion trap mass spectrometer. To identify high-confidence interactors, CompPASS analysis^47^ was performed against mass spectrometry results from unrelated Flag immunoprecipitates performed in our laboratory. For Fig. 1e, protein spectral counts were normalized to the total spectral counts, multiplied by 10^6^, added 1 and the log_2_ was taken (log_2_((spectral counts_protein_/total spectral counts) × 10^6^ + 1). Proteins with more than 2 spectral counts and a CompPASS *z* score > 80% of max *z* score in Flag–UBR4 sample (Flag–UBR4 is an average of 2 biological replicates) or 3 spectral counts and a CompPASS *z* score > 80% of max *z* score in Flag–KCMF1 sample were plotted on a scatter plot. For Extended Data Fig. 2d, we normalized values in a similar manner but used spectral counts of the bait instead of total spectral counts. A subset of the identified interactors are plotted in Extended Data Fig. 2d. Total spectral counts and *z* scores computed using CompPASS are available in Supplementary Table 2.

### Growth competition assays

HEK293T and Δ*UBR4* cells were transduced to express either GFP or mCherry using the lentiviral pLVX-GFP-P2A-Blasticidin or pLVX-mCherry-P2A-Blasticidin vector, respectively. For sgRNA depletion competition assays, 5 × 10^4^ WT–GFP and 5 × 10^4^ Δ*UBR4*–mCherry cells were mixed in 24-well plates and spin-infected with lentiviral particles as described above. After 24 h, viral supernatants were removed and cells were expanded to 6-well plates and selected with puromycin for 5 days. Competition assays were conducted for 12 days after selection. When indicated, ISRIB was added throughout the competition assay after antibiotic selection. The percentage of mCherry^+^ cells and GFP^+^ cells was determined using a BD LSRFortessa instrument, analysed using FlowJo 10.8.1 and normalized to the sgCNTRL ratio. The ratio of mCherry-labelled to GFP-labelled cells is reported as (Δ*UBR4*_sgRNA_/WT_sgRNA_)/(Δ*UBR4*_sgCNTRL_/WT_sgCNTRL_) for each sgRNA tested.

For drug competition assays, 5 × 10^4^ WT–GFP and 5 × 10^4^ Δ*UBR4*–mCherry cells were mixed in 6-well plates. The next day, indicated drugs were added for 72 h. The ratio of mCherry^+^/GFP^+^ cells was determined using a BD LSRFortessa instrument, analysed using FlowJo 10.8.1 and normalized to the untreated sample. The ratio of mCherry-labelled to GFP-labelled cells is reported as (Δ*UBR4*_treatment_/WT_treatment_)/(Δ*UBR4*_control_/WT_control_). For growth in DMEM + galactose, competition assays were performed for 11 days and the mCherry/GFP ratio was normalized to the ratio of growth in DMEM + glucose. Gating strategies for flow cytometry analysis are shown in Supplementary Fig. 2.

### Drug treatments

For 3-day growth competition experiments with drug-treated cells, we used the following drug concentrations: 2.5 μM sodium arsenite (Ricca Chemical, 714216); 2.5 μM oligomycin A (Santa Cruz Biotechnology, sc-201551); 50 nM rotenone (Sigma-Aldrich, R8875-1G); 10 μM CCCP (Cayman Chemicals, 25458); 5 μM BTdCPU (EMD Millipore, 324892); 10 nM thapsigargin (Sigma-Aldrich, T9033-.5MG); 100 nM tunicamycin (Calbiochem, 65438010); 1.25 μM EN6 (Sigma-Aldrich, SML2689-5MG)^48^; 4 nM bafilomycin A1 (Selleck Chemicals, S1413); and 40 nM 17-DMAG (Selleck Chemicals, S1142). For overnight drug treatments, we used 5 μM sodium arsenite, 10 μM CCCP, 0.2 μM oligomycin, 5 μM antimycin A (Santa Cruz Biotechnology, sc-202467) or otherwise indicated in the figure legends. To inhibit the proteasome or autophagy, we used 2 μM carfilzomib (Selleck Chemicals, S2853) for 6 h or 700 nM bafilomycin A1 for 6 h, respectively. ISRIB (Sigma-Aldrich, SML0843) was used at a concentration of 200 nM.

### Mitochondrial import assay

Mitochondrial split-GFP import flow-cytometry-based assays measuring reconstitution of GFP after transport of a GFP11-tagged protein into the mitochondrial matrix were performed based on previously described imaging experiments^20^. HEK293T and Δ*UBR4* cells were transfected with MTS-mScarlett-GFP1-10-IRES-Puro and seeded in 96-well plates at a density of one cell per well and selected for individual clones with random integration using puromycin selection. Single-cell clones with identical expression of mScarlett determined by flow cytometry were selected and used for further experiments. Cells were transfected with 0.5 μg of inducible GFP11 reporter constructs (TRAP1-GFP11-IRES-BFP, HMT2-GFP11-IRES-BFP or CS-GFP11-IRES-BFP) and 1.5 μg of empty vector construct using Lipofectamine 3000. Expression was induced by addition of doxycycline (1 μg ml^–1^) after 24 h. Flow cytometry was performed after another 24 h of incubation using a BD LSRFortessa instrument. Mitochondrial import was calculated as a function of the GFP^+^/BFP^+^ ratio in mScarlett^+^ cells. Gating strategies for flow cytometry analysis are shown in Supplementary Fig. 2.

### Protein stability reporter assay

The pCS2+-degron-GFP-IRES-mCherry reporter constructs were generated as previously described^21^. The ISR reporter was designed as previously described^23^. All pCS2-degron-GFP-IRES-mCherry constructs are listed in Supplementary Table 4. A library of GFP-tagged candidate targets (associated with Fig. 2b) included proteins that are genetic and physical interactors of SIFI as well as proteins anticorrelated with SIFI subunits in proteomics analyses^49^ or across genetic screens (DepMap). Cells were seeded in 6-well plates at a density of 200,000 cells. The next day, 40 ng of reporter plasmid and empty vector up to 400 ng total were transfected into HEK293T cells on 6-well plates using PEI and collected for flow cytometry after 48 h. When siRNA depletions were carried out, 200,000 cells were seeded in 6-well plates. The next day siRNA transfections were performed using Lipofectamine RNAiMAX as described above. The following day, 50 ng of reporter and empty vector up to 500 ng total DNA were transfected using Lipofectamine 3000 according to the manufacturer’s instructions. After 24 h of reporter transfection, cells were collected and processed for flow cytometry. Cells were analysed using either a BD Bioscience LSR Fortessa or a LSR Fortessa X20, and the GFP/mCherry ratio was analysed using FlowJo. Gating strategies for flow cytometry analysis are shown in Supplementary Fig. 2.

### Western blotting

For western blot analysis of whole cell lysates, cells were collected at indicated time points by washing in PBS, pelleting and snap freezing. Cells were lysed in lysis buffer (150 mM NaCl, 50 mM HEPES pH 7.5 and 1% NP-40 substitute) supplemented with Roche complete protease inhibitor cocktail (Sigma, 11836145001), PhosSTOP phosphatase inhibitor cocktail (Roche, 4906837001), carfilzomib (2 μM) and benzonase (EMD Millipore, 70746-4) on ice. Samples were then normalized to protein concentration using Pierce 660 nm Protein Assay reagent (Thermo Fisher, 22660). Next, 2× urea sample buffer (120 mM Tris pH 6.8, 4% SDS, 4 M urea, 20% glycerol and bromophenol blue) was added to the samples. SDS–PAGE and immunoblotting were performed using the indicated antibodies. Images were captured using a ProteinSimple FluorChem M device.

### Small-scale immunoprecipitations

Cells were collected after washing in PBS, pelleted and snap frozen. Frozen pellets were resuspended in lysis buffer (40 mM HEPES pH 7.5, 100 mM NaCl, 0.1% NP40, with Roche complete protease inhibitor cocktail (Sigma-Aldrich, 11873580001), PhosSTOP phosphatase inhibitor cocktail (Roche, 4906837001), carfilzomib (2 μM, Selleckchem, S2853) and benzonase (EMD Millipore, 70746-4). Lysates were incubated for 20 min on ice and cleared by centrifugation for 20 min at 21,000*g*, 4 °C. Supernatants were normalized to volume and protein concentration, and 5% of the sample was removed as input and the sample was added to equilibrated anti-Flag-M2 Affinity Agarose Gel slurry (Sigma-Aldrich, A2220) and rotated for 1–2 h at 4 °C. Beads were washed 3× and eluted with 2× urea sample buffer. SDS–PAGE and immunoblotting were performed using the indicated antibodies. Images were captured using a ProteinSimple FluorChem M device.

### His-ubiquitin immunoprecipitation

Five 15-cm plates of WT HEK293T or Δ*UBR4* cells were transfected 2 days before collection with 2 μg of pcs2-HRI-3×Flag and 10 μg of pcs2-His-ubiquitin per 15 cm plate. Cells were treated with carfilzomib (2 μM) for 6 h, collected and flash frozen. Cells were lysed in 1 ml of 8 M urea lysis buffer (8 M urea, 300 mM NaCl, 0.5% NP-40, 50 mM Na_2_HPO_4_, 50 mM Tris-HCl pH 8, 10 mM imidazole, 10 mM *N*-ethylmaleimide (Sigma-Aldrich, E3876), with Roche complete protease inhibitor cocktail (Sigma-Aldrich, 11873580001), PhosSTOP phosphatase inhibitor cocktail (Roche, 4906837001), carfilzomib (2 μM, Selleckchem, S2853)) and incubated at room temperature for 20 min. Samples were sonicated at 20 Amp for 10 s (1 s on/1 s off). Samples were centrifuged at 15,000*g* for 15 min at room temperature and supernatants were normalized to volume and protein concentration. Next, 5% of the sample was removed as input and the sample was added to equilibrated Ni-NTA resin and rotated for 4 h at room temperature. Resin was washed twice with wash buffer (8 M urea, 300 mM NaCl, 50 mM Na_2_HPO_4_ and 50 mM Tris-HCl pH 8) containing 20 mM imidazole and once with wash buffer containing 40 mM imidazole, and eluted with Laemmli sample buffer containing 200 mM imidazole. SDS–PAGE and immunoblotting were performed using the indicated antibodies. Images were captured using a ProteinSimple FluorChem M device.

### Antibodies

The following antibodies were used for immunoblot analyses: anti-Flag (mouse, clone M2, Sigma-Aldrich, F1804; dilution 1:1,000); anti-Flag (rabbit, Cell Signaling Technology (CST), 2368; dilution 1:1,000); anti-HA-tag (rabbit, C29F4, CST, 3724; dilution 1:1,000); anti-GAPDH (rabbit, D16H11, CST, 5174; dilution 1:1,000); anti-α-tubulin (mouse, DM1A, Calbiochem, CP06; dilution 1:1,000); anti-UBR4/p600 (rabbit, A302, Bethyl, A302-277A; dilution 1:1,000); anti-UBR4/p600 (rabbit, A302, Bethyl, A302-278A; dilution 1:1,000); anti-UBR4/p600 (rabbit, A302, Bethyl, A302-279A; dilution 1:1,000); anti-PKR (mouse, B-10, Santa Cruz, sc-6282; dilution 1:200); anti-GCN2 (mouse, F-7, Santa Cruz, sc-374609; dilution 1:200); anti-PERK (mouse, B-5, Santa Cruz, sc-377400; dilution 1:200); anti-UBE2A/B (mouse, G-9, Santa Cruz, sc-365507; dilution 1:150); anti-ATF4 (rabbit, D4B8, CST, 11815S; dilution 1:1,000); anti-EIF2AK1 (rabbit, Proteintech, 20499-1-AP; dilution 1:1,000), anti-SSBP1 (rabbit, Proteintech, 12212-1-AP; dilution 1:1,000); anti-TIM8A (rabbit, Proteintech, 11179-1-AP; dilution 1:500); anti-KCMF1 (rabbit, Sigma, HPA030383, dilution 1:1,000); anti-NIPSNAP3A (rabbit, Thermo Fisher, PA5-20657; dilution 1:1,000); anti-GADD34 (rabbit, Proteintech 10449-1-AP, dilution 1:1,000); anti-CReP (rabbit, Proteintech 14634-1-AP; dilution 1:1,000); anti-ubiquitin (rabbit, CST, 43124; dilution 1:1,000); goat anti-rabbit IgG (H+L) HRP (Vector Laboratories, PI-1000; dilution 1:5,000); sheep anti-mouse IgG (H+L) HRP (Sigma, A5906; dilution 1:5,000); and goat anti-mouse IgG light-chain-specific HRP conjugated (Jackson Immunoresearch, 115-035-174; dilution 1:5,000). The following antibodies were used for immunofluorescence: anti-TOM20 antibody (rabbit, Proteintech 11802-1-AP; dilution 1:500) and secondary antibody goat anti-rabbit AF647 (Thermo Fisher, A21245; dilution 1:500).

### In vitro transcription/translation of substrates

In vitro synthesized substrates were all cloned into pCS2 vectors containing a SP6 promoter, as previously described^50^, and are summarized in Supplementary Table 4. The SUMO tag was appended to HRI and DELE1 for solubility. ^35^S-labelled substrates were generated by incubating 2.5 µg of plasmid DNA in 10 µl of wheat germ extract (Promega, L3260) supplemented with 2 µM carfilzomib and 1 µl of ^35^S-Met (PerkinElmer, NEG009H001MC) for 2 h at 25 °C. ^35^S-labelled substrates were used for in vitro ubiquitylation assays.

### In vitro ubiquitylation assays

For in vitro ubiquitylation assays, human SIFI complex was purified using an endogenous Flag–UBR4 HEK293T cell line. Each in vitro ubiquitylation reaction required material from 2.5 15-cm plates of Flag–UBR4 cells. Frozen cell pellets were lysed at 4 °C for 30 min in 1 ml of lysis buffer per 10 15-cm plates (40 mM HEPES, pH 7.5, 5 mM KCl, 150 mM NaCl, 0.1% Nonidet P-40, 1 mM DTT, 1× complete protease inhibitor cocktail, 2 μM carfilzomib and 4 μl of benzonase per 10 15-cm plates). Lysed extracts were pelleted at 21,000*g* to remove cellular debris and the clarified lysate was bound to anti-Flag M2 resin (20 μl of slurry per 2.5 15-cm plates of material) for 2 h rotating at 4 °C. UBR4-coupled beads were washed 2× with detergent (40 mM HEPES, pH 7.5, 5 mM KCl, 150 mM NaCl, 0.1% Nonidet P-40, 1 mM DTT) and 2× without detergent (40 mM HEPES, pH 7.5, 5 mM KCl, 150 mM NaCl and 1 mM DTT). All liquid was removed from the beads using a crushed gel loading tip before addition of the in vitro ubiquitylation reaction.

In vitro ubiquitylation assays were performed in a 10 μl reaction volume: 0.5 μl of 10 μM E1 (250 nM final), 0.5 μl of 50 μM UBE2A (2.5 μM final), 0.5 μl of 50 μM UBE2D3 (2.5 μM final), 1 μl of 10 mg ml^–1^ ubiquitin (1 mg ml^−1^ final) (R&D Systems, U-100H), 0.5 μl of 200 mM DTT, 1.5 μl of energy mix (150 mM creatine phosphate (Sigma-Aldrich, 10621714001-5G), 20 mM ATP, 20 mM MgCl_2_, 2 mM EGTA, pH to 7.5 with KOH), 1 μl of 10× ubiquitylation assay buffer (250 mM Tris pH 7.5, 500 mM NaCl and 100 mM MgCl_2_), 0.5 μl of 1 mg ml^–1^ tandem ubiquitin binding entities (TUBEs) were pre-mixed and added to 10 μl of UBR4-coupled bed resin. Next, 3 μl of in vitro translated substrate or 1 μl of 100 µM TAMRA-labelled peptide was added to the reactions. Competitor proteins or peptides, or 1× PBS was added to reach final volume of 10 μl. Peptide sequences used in this study are summarized in Supplementary Table 7. Reactions were performed at 30 °C with shaking for 2 h. Reactions were stopped by adding 2× urea sample buffer and resolved on SDS–PAGE gels before autoradiography. TAMRA-labelled peptide ubiquitylation assays were run on 4–20% gradient gels (Thermo Fisher, EC6026BOX) and imaged using a ProteinSimple Fluorchem M imager. To test ubiquitin linkage specificity of SIFI, we used commercially available recombinant human ubiquitin mutants (R&D Systems, UM-K6R, UM-K11R, UM-K27R, UM-K29R, UM-K33R, UM-K48R, UM-K480, UM-K63R, UM-NOK, UM-K60, UM-K110, UM-K270, UM-K290, UM-K330 and UM-K630). E1 enzyme UBA1 was purified as previously described^51^. UBE2A, UBE2D3, TUBE, TOM20 WT and TOM20(I74S,V109S) recombinant proteins were purified as described below.

### Recombinant protein purification

Human UBE2A and UBE2D3 were cloned into a pET28a His-tagged expression vector (pET28a-6×His-UBE2A, pET28a-6×His-UBE2D3) and were expressed in LOBSTR-BL21(DE3)-RIL cells. TUBEs were expressed from the pET28a-6×His-TEV-HALO-4×UbiquilinUBA in LOBSTR-BL21(DE3)-RIL cells. Protein expression was induced at OD_600_ = 0.6 with 250 μM IPTG for 16 h at 18 °C. Cells were lysed in lysis buffer (50 mM HEPES pH 7.5, 500 mM NaCl, 10 mM imidazole,10% glycerol, 5 mM BME, 1× PMSF (Sigma-Aldrich, P7626), 1 mg ml^–1^ lysozyme (Sigma-Aldrich, L6876-10G) and benzonase) by sonication. Lysates were clarified by centrifugation before 90 min of incubation with equilibrated Ni-NTA agarose beads (Qiagen, 20350). Beads were washed 3× in wash buffer (50 mM HEPES pH 7.5, 500 mM NaCl, 10% glycerol and 5 mM BME) with increasing concentration of imidazole (20 mM, 40 mM and 60 mM). Proteins were eluted in wash buffer and 250 mM imidazole and dialysed overnight using dialysis cassettes (Thermo Fisher, 66380) in storage buffer (50 mM HEPES pH 7.5, 150 mM NaCl, 10% glycerol and 2 mM DTT). TEV protease (at 1 μg:100 μg TEV to protein ratio, UC Berkeley QB3 MacroLab) was added to the HALO-TEV-TUBEs during dialysis. The next day, TUBE protein was bound to equilibrated Ni-NTA agarose beads, and the flow-through was collected to remove TEV protease and uncleaved proteins. Dialysed proteins were concentrated using Amicon Ultra-4 3 K (UBE2A, UBE2D3) and 10 K (TUBEs) (Sigma-Aldrich, UFC800324, UFC801024), flash-frozen and stored at −80 °C for future use.

His-SUMO-TEV-TOM20(62–128) and His-SUMO-TEV-TOM20(62–128,I74S,V109S) were cloned into a pET28a His-tagged expression vector (pET28a-6×His-SUMO-TOMM20, pET28a-6×His-SUMO-TOMM20(I74S,V109S)) and were expressed in LOBSTR-BL21(DE3)-RIL cells. Protein expression was induced at log phase with 250 μM IPTG for 16 h at 18 °C. Cells were lysed in lysis buffer (50 mM HEPES pH 7.5, 150 mM NaCl, 10 mM imidazole, 5 mM BME and 1 mM PMSF) using a LM10 Microfluidizer. Lysate was clarified before 1 h of incubation with equilibrated Ni-NTA agarose beads, and beads were washed in wash buffer (50 mM HEPES pH 7.5, 150 mM NaCl, 5 mM BME and 20 mM imidazole) and proteins were eluted in wash buffer containing 250 mM imidazole, dialysed overnight in dialysis cassettes in dialysis buffer (50 mM HEPES pH 7.5, 150 mM NaCl and 5 mM BME) containing TEV protease (at 1 μg:100 μg TEV to protein ratio, UC Berkeley QB3 MacroLab). The next day, dialysed protein was bound to equilibrated Ni-NTA agarose beads, and the flow-through was collected to remove TEV protease and uncleaved proteins. The flow-through was run on a S75 column (50 mM HEPES pH 7.5, 150 mM NaCl and 1 mM TCEP). Fractions containing the proteins were run on Coomassie for validation, concentrated with Amicon Ultra-4 3 K, aliquoted, flash-frozen and stored at −80 °C for future use.

### RNA-seq sample preparation and analysis

WT sgCNTRL*,* Δ*UBR4* sgCNTRL*,* WT sg*TIMM8A,* Δ*UBR4* sg*TIMM8A,* ISRIB-treated (200 nM, 16 h) Δ*UBR4* sg*TIMM8A* and arsenite-treated (5 µM, 16 h) WT sgCNTRL and Δ*UBR4* sgCNTRL cells were collected after washing in PBS, pelleted and snap-frozen. Three biological replicates were processed for each condition. Total RNA was extracted using a nucleospin RNA kit (Macherey-Nagel, 740955). Library preparation and deep sequencing were performed by Novogene. In brief, mRNA was purified from total RNA using polyT oligonucleotide attached magnetic beads. mRNA was fragmented and first-strand synthesis was performed with random hexamers followed by second-strand cDNA synthesis. This was followed by end repair, A-tailing, adapter ligation, size selection, amplification and purification. Libraries were sequenced by paired-end sequencing on an Illumina NovaSeq sequencer.

To obtain transcript abundance counts, sequencing reads were mapped to the human reference transcriptome (GRCh38, Ensembl Release 96) using Kallisto (v.0.48.0). Gene-level count estimates were obtained by summing counts or TPMs across all transcripts from a given gene. Differential gene-expression analysis was performed using DESeq2 (ref. ^52^) ran on the Galaxy server (Galaxy v.2.11.40.7)^53^ using the WT sgCNTRL as control for all samples. DESeq2 analysis results are provided in Supplementary Table 3. Genes with >1 TPM were retained for subsequent analysis. Genes significantly differentially expressed (*P* adjusted < 0.05), showing at least a twofold change, in the WT sgCNTRL cells treated with sodium arsenite were selected. Hierarchical clustering was performed in Custer (v.3.0)^54^ and results were visualized using Java Treeview^55^. HEK293T WT sgCNTRL and WT sg*HRI* treated with oligomycin from ref. ^23^ (NCBI Gene Expression Omnibus (GEO) identifier: GSE134986) were also clustered and used to isolate the upregulated ISR genes cluster. Raw and processed data have been deposited to the GEO under accession number GSE232191.

### qPCR

Total RNA was purified using a nucleospin RNA kit (Macherey-Nagel, 740955). cDNA was generated using a RevertAid First Strand cDNA Synthesis kit (Thermo Fisher Scientific, K1622) and RT–qPCRs were performed on a LightCycler 480 II Instrument (Roche) using 2× KAPA SYBR Fast qPCR master mix (Roche, KK4602). Fold changes in expression were calculated using the ΔΔC_t_ method. qPCR primer sequences are presented in Supplementary Table 5.

### Immunofluorescence and confocal microscopy

U2OS cells were seeded on 12-mm glass coverslips (Fisher Scientific, 1254580) at 100,000 cells per well in a 12-well plate. Cells were transfected the next day with pCS2-HRIhelix2-GFP-IRES-mCherry using Lipofectamine 3000. Medium was changed 24 h after transfection. At 48 h after transfection, cells were fixed in a solution of 4% paraformaldehyde in 1× dPBS for 20 min, followed by permeabilization with 0.3% Triton X-100 in 1× dPBS for 20 min, and finally blocked with 10% FBS in 1× dPBS for 30 min. Samples were probed with anti-TOM20 antibody (1:500) for 3 h in 1× dPBS, 10% FBS and 0.1% Triton X-100. Samples were incubated with secondary antibody goat anti-rabbit AF647 (1:500, Thermo Fisher, A21245) and stained with Hoechst 33342 (1:3,000, Anaspec, 83218) for 1 h. All sample processing was carried out at room temperature. Coverslips were mounted onto microscope slides with ProLong gold (Thermo Fisher, P36930) and imaged using a Zeiss LSM 900 with Airyscan 2 microscope. Images were captured with a ×63 oil objective and Airyscan SR. Images were processed using Zen Blue (Zeiss) Airyscan processing and Fiji.

### Software and code for data analysis

The following freely or commercially available software and codes were used to analyse data: FlowJo (v.10.8.1), GraphPad Prism (v.9), ImageJ2 (v.2.9.0/1.53t), Cytoscape ClueGO (v.3.7.1), CasTLE (v.1.0), Kallisto (v.0.48.0), DESeq2 (Galaxy v.2.11.40.7), Cluster 3.0 and Java TreeView (v.1.1.6r4).

### Reporting summary

Further information on research design is available in the Nature Portfolio Reporting Summary linked to this article.

## Online content

Any methods, additional references, Nature Portfolio reporting summaries, source data, extended data, supplementary information, acknowledgements, peer review information; details of author contributions and competing interests; and statements of data and code availability are available at 10.1038/s41586-023-06985-7.

### Supplementary information

Supplementary Information Supplementary Fig. 1 contains the uncropped gels and Supplementary Fig. 2 shows the gating strategy for flow cytometry experiments.

Reporting Summary

Supplementary Tables Supplementary Tables 1–7 comprising source data for the whole genome CRISPR screen (Table 1), source data for proteomics data (Table 2), source data for RNA-seq experiments (Table 3), plasmid list (Table 4), qPCR primer sequences (Table 5), six siRNA sequences (Table 6) and synthetic peptide sequences (Table 7).

## Data Availability

Source data for immunoblots are provided in Supplementary Fig. 1. Gating strategies for flow cytometry experiments are provided in Supplementary Fig. 2. Source data for the CRISPR screen are provided in Supplementary Table 1. Immunoprecipitation and mass spectrometry source data (associated with Fig. 1e and Extended Data Fig. 2d) are provided in Supplementary Table 2. RNA-seq data (associated with Fig. 3 and Extended Data Fig. 3h) have been deposited into the GEO (accession number GSE232191). Source data for this RNA-seq analysis are also provided in Supplementary Table 3. The human reference transcriptome (GRCh38, Ensembl Release 96), which was used to align the RNA-seq data can be accessed at Ensembl (http://apr2019.archive.ensembl.org/Homo_sapiens/Info/Index). The previously published RNA-seq data of HEK293T WT sgCNTRL cells and sg*HRI* cells treated with oligomycin^23^ can be accessed at the GEO (accession number GSE134986). There are no restrictions on data availability.

## Code availability

Custom Python scripts used to plot Fig. 1b,e are available from the corresponding author on request.
