# Supplementary material for: Stress response silencing by an E3 ligase mutated in neurodegeneration
Source: Nature. 2024 Jan 31;626(8000):874–80. doi: 10.1038/s41586-023-06985-7 (PMC10881396; doi:10.1038/s41586-023-06985-7)
Supplement: Supplementary file 1 — Supplementary Fig. 1 contains the uncropped gels and Supplementary Fig. 2 shows the gating strategy for flow cytometry experiments. [file 41586_2023_6985_MOESM1_ESM.pdf]

nature portfolio

https://doi.org/10.1038/s41586-023-06985-7

Supplementary information

Stress response silencing by an E3 ligase mutated in neurodegeneration

In the format provided by the authors and unedited

Figure 2d

Chemiluminescence **Chemiluminescence + visible light**

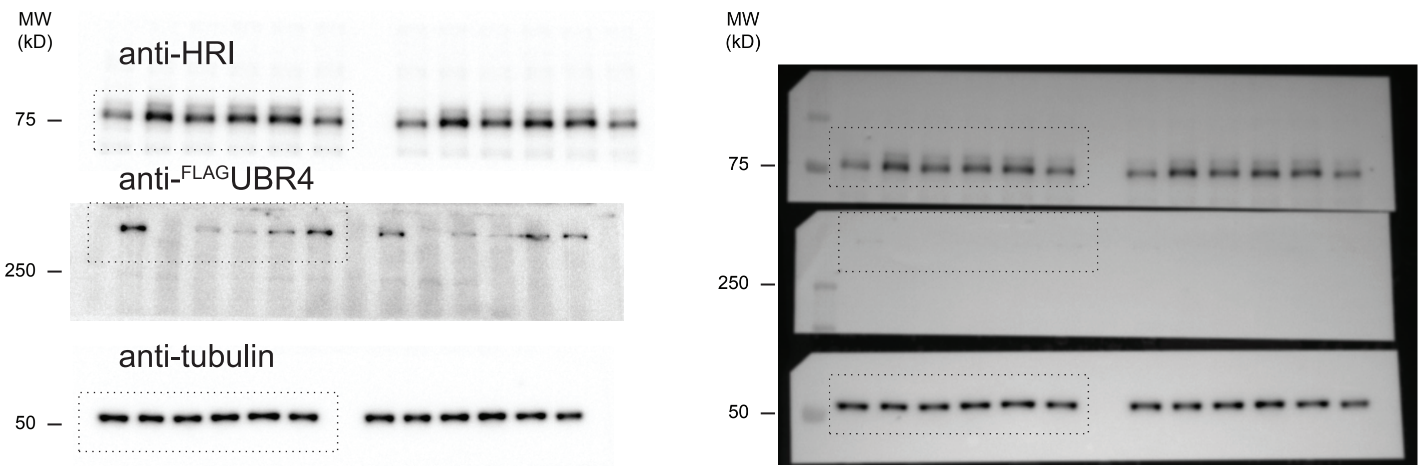

The loading control tubulin was run on the same gel as FLAGUBR4 and HRI.

Figure 2e

Chemiluminescence **Chemiluminescence + visible light**

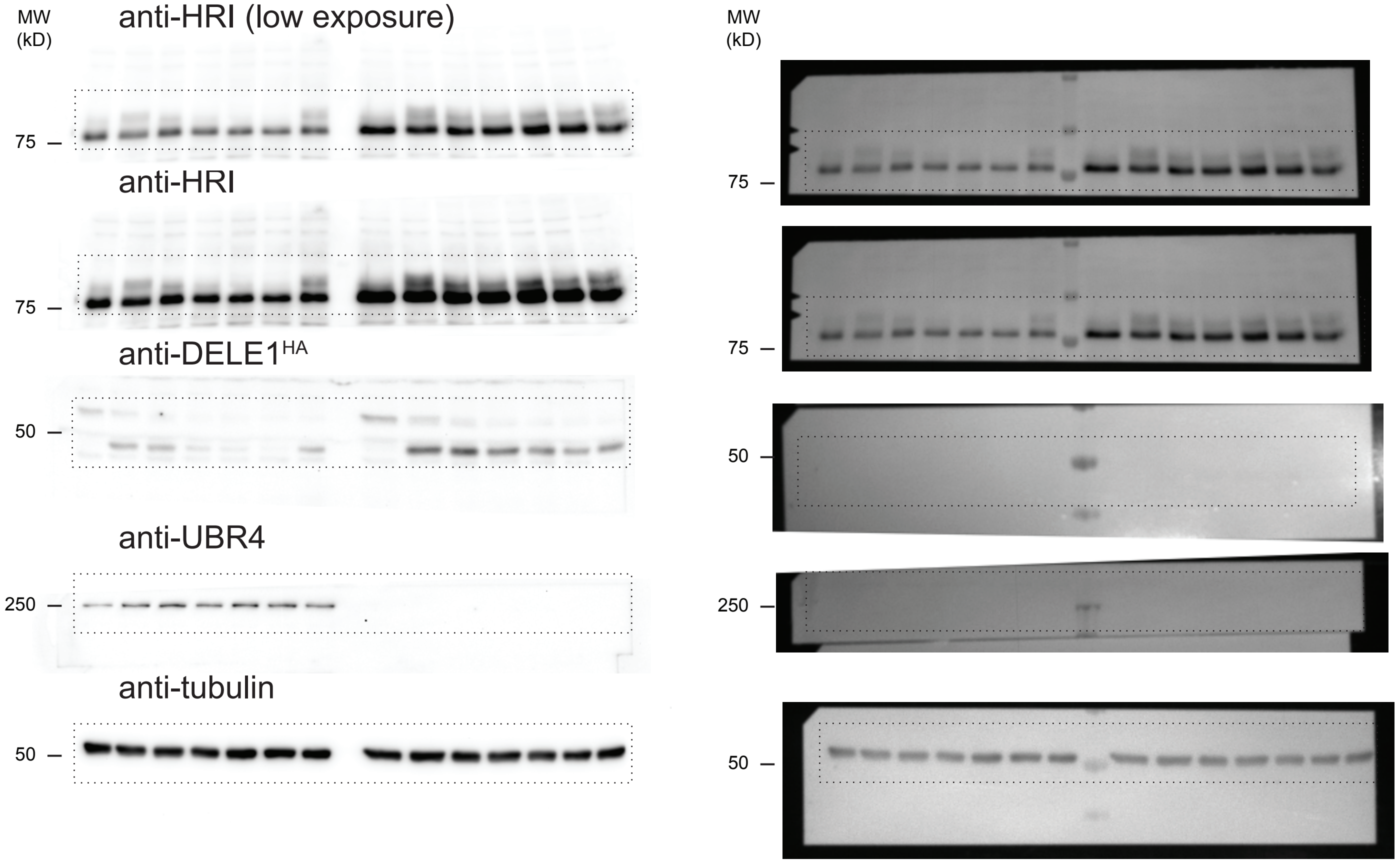

The loading control tubulin was obtained by stripping the DELE1HA membrane, which was run on the same gel as UBR4.

Figure 2f

Raw files | **Contrasted raw files to discern typically low S^{35} autoradiography signal**

HRI-sumo autoradiography

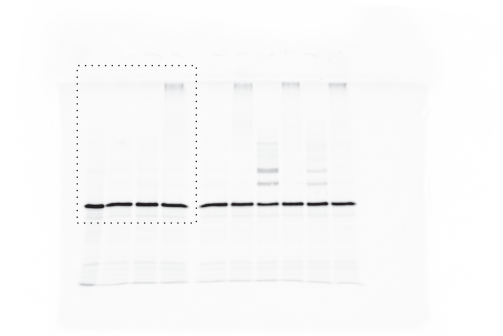

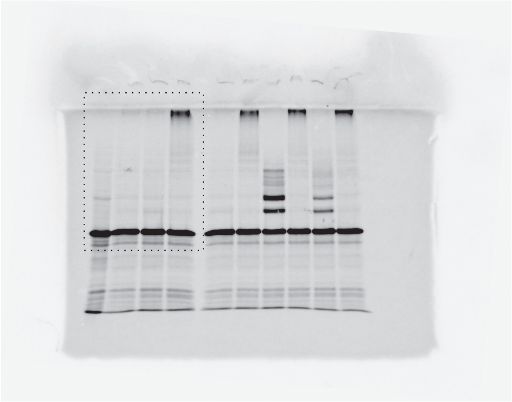

DELE1-sumo autoradiography

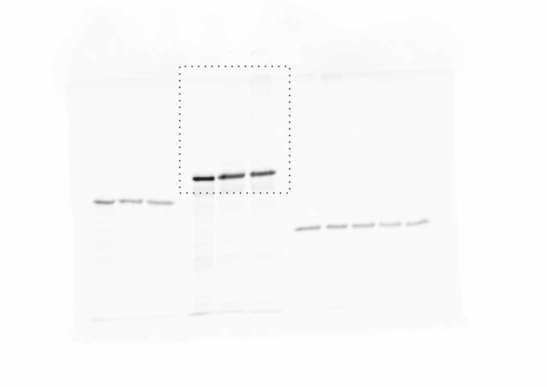

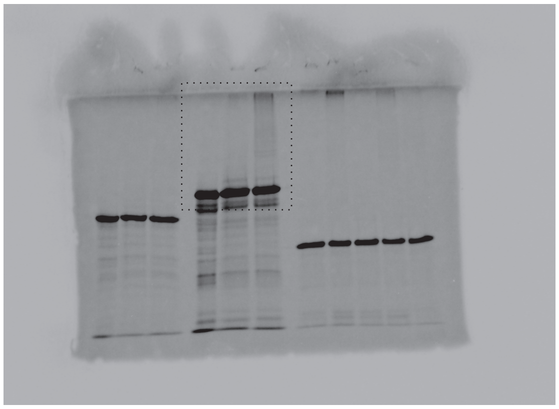

Figure 2g

HRI-sumo autoradiography

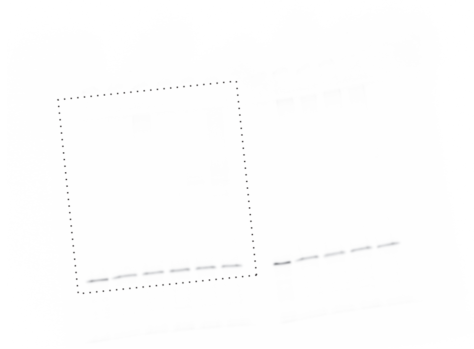

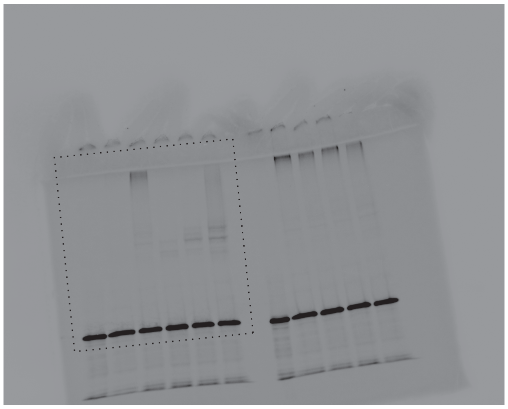

Figure 2h

HRI-sumo autoradiography

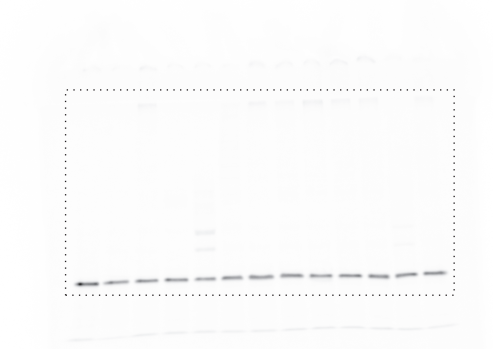

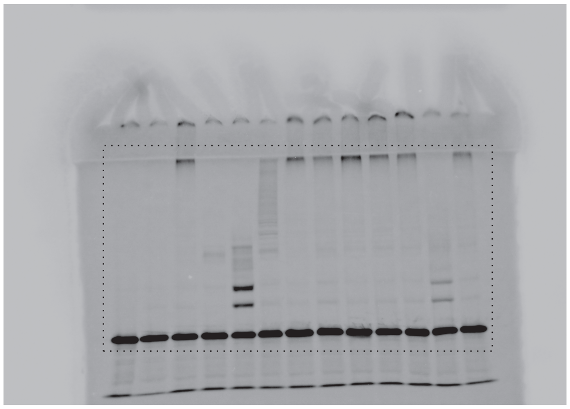

Figure 3b

Chemiluminescence

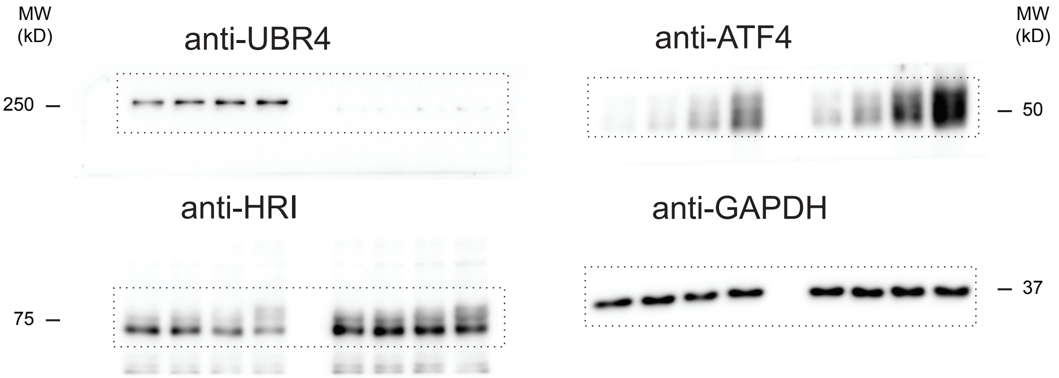

Chemiluminescence + visible light

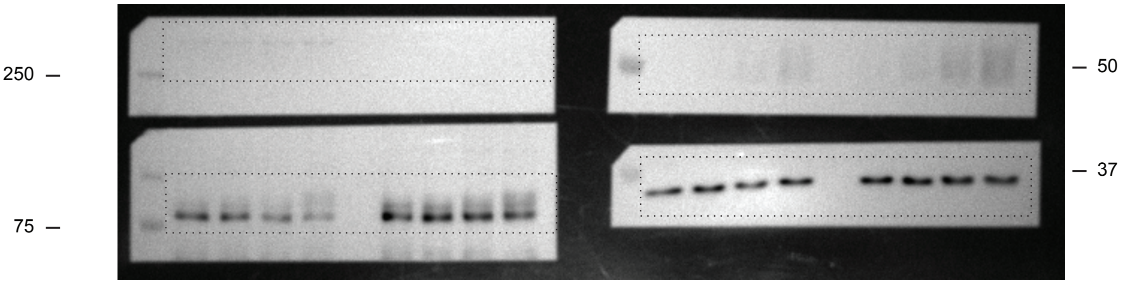

The loading control GAPDH was run on the same gel as UBR4, HRI and ATF4.

Figure 3c

Chemiluminescence

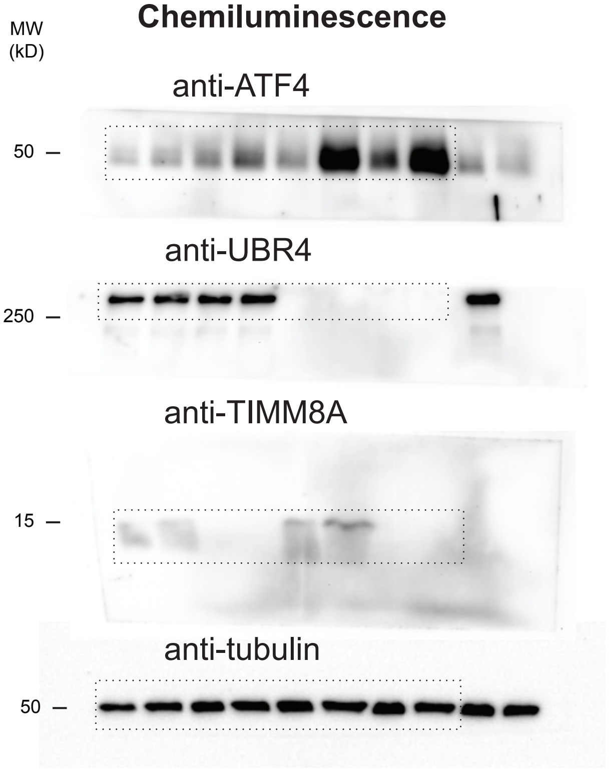

Chemiluminescence + visible light

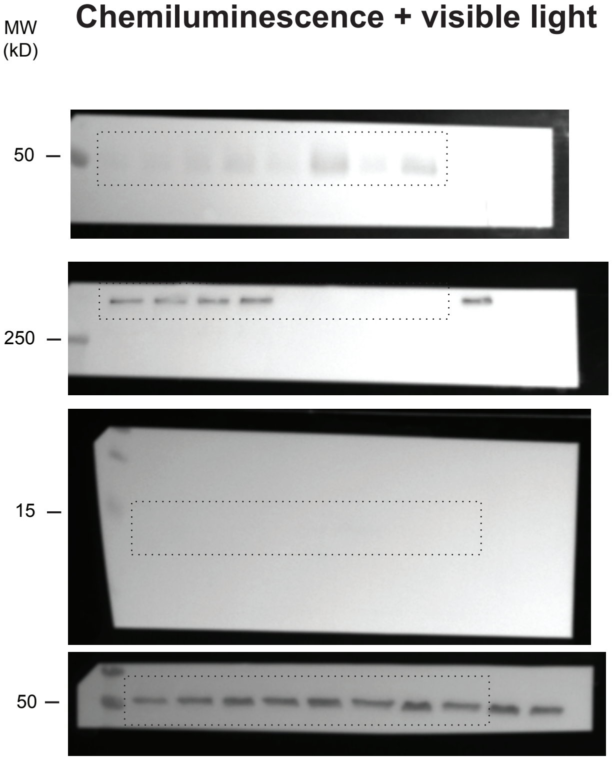

The loading control tubulin was run on the same gel as TIMM8A.

Figure 3f

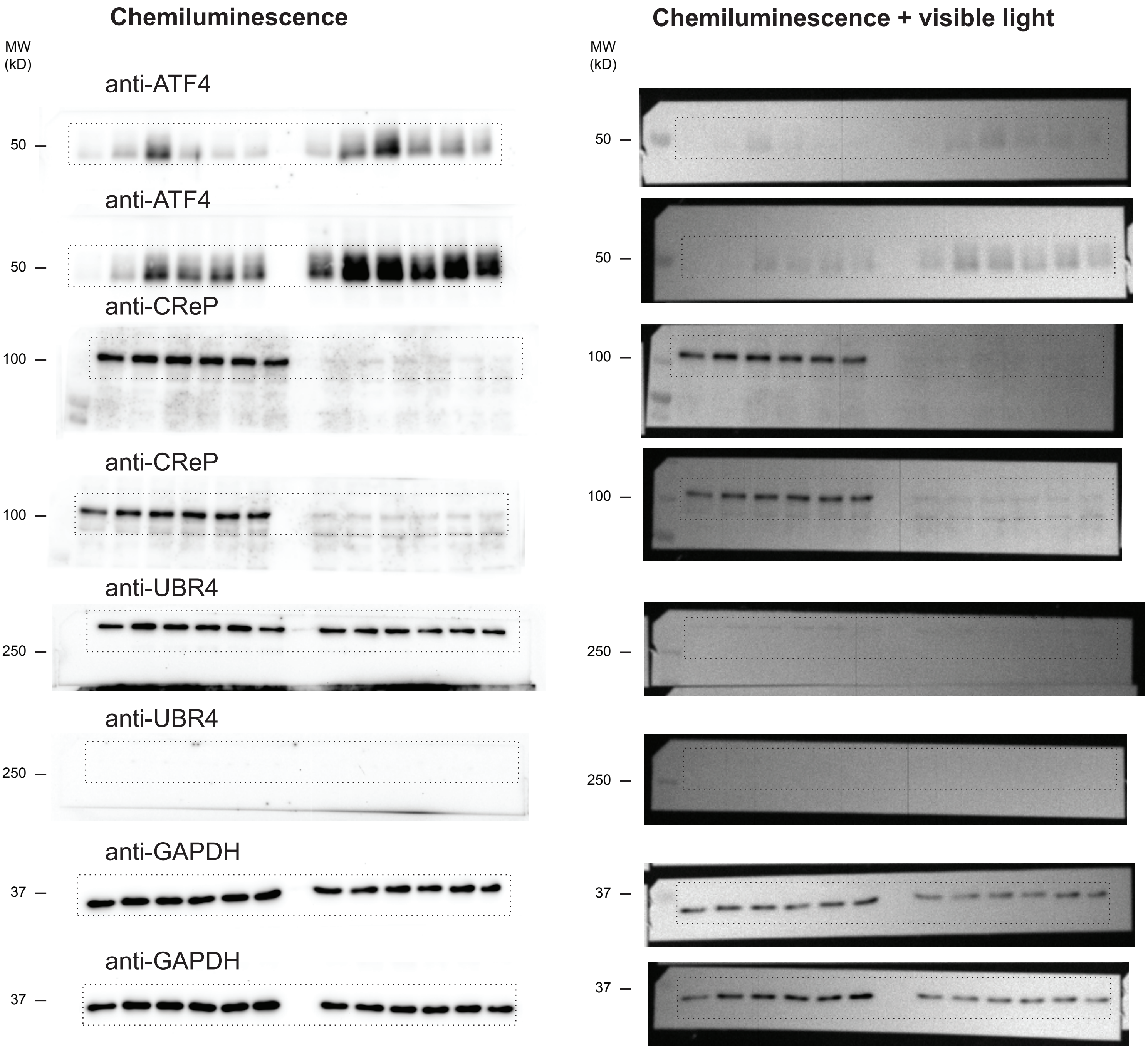

The loading controls GAPDH were run on the same gels as ATF4, CReP and UBR4 in wt and *ΔUBR4* respectively.

Figure 4d

Raw file

HRI-sumo autoradiography

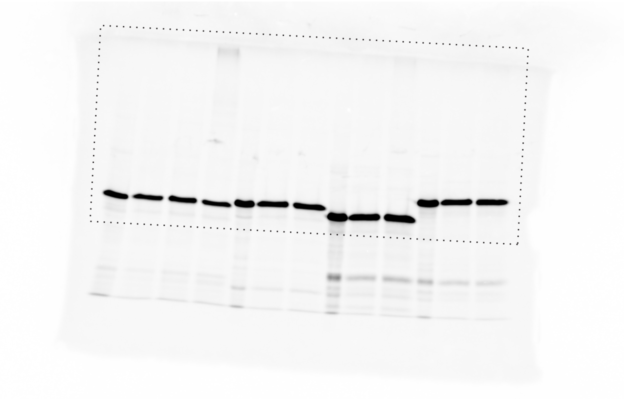

Contrasted raw files to discern typically low S^{35} autoradiography signal

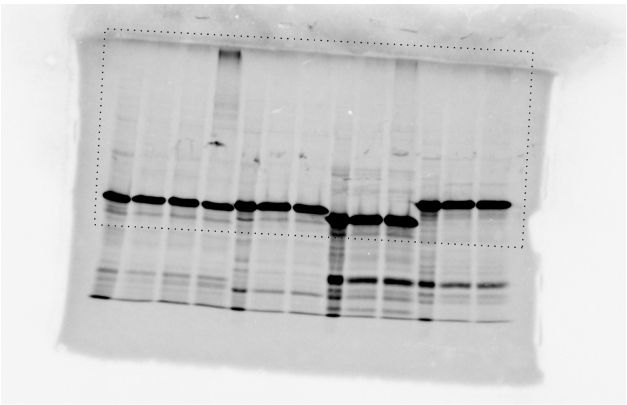

Figure 4e

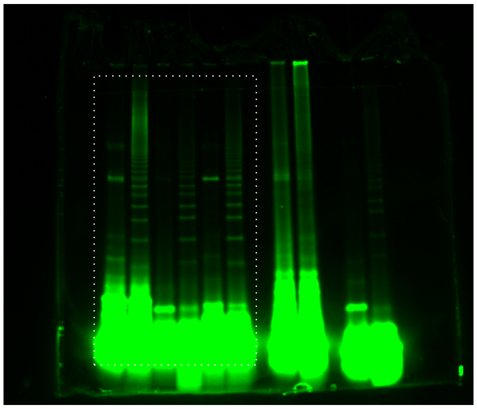

Figure 4g

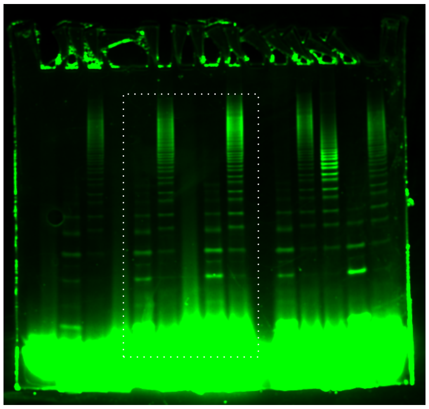

Figure 4h

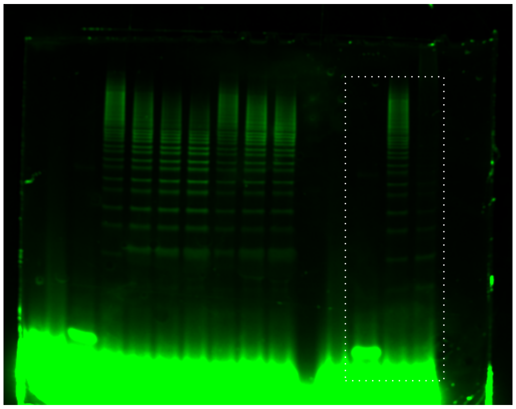

Figure 4i

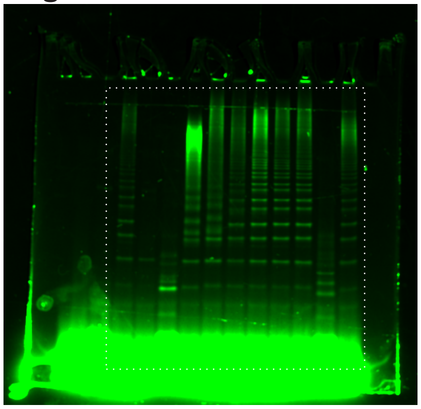

Figure 5d

Raw file

HRI-sumo autoradiography

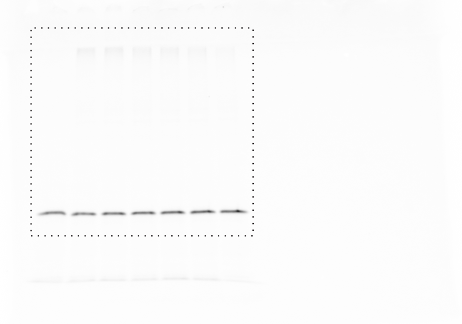

Contrasted raw files to discern typically low S^{35} autoradiography signal

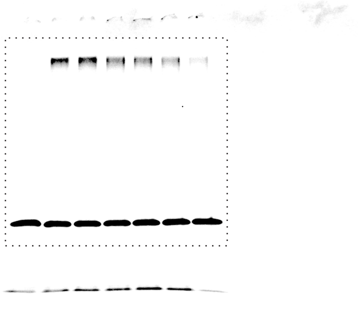

Extended Data Figure 1a

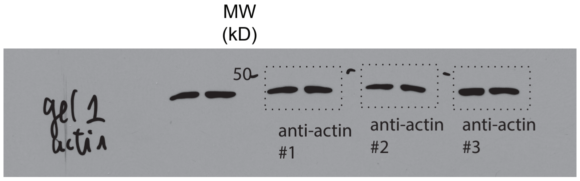

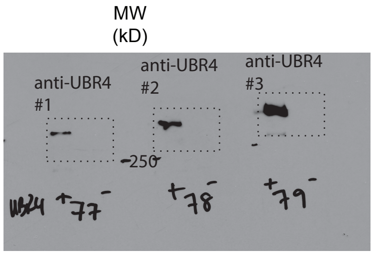

The loading control actin was blotted from the same gel as UBR4 samples.

Extended Data Figure 2a

Chemiluminescence

Chemiluminescence + visible light

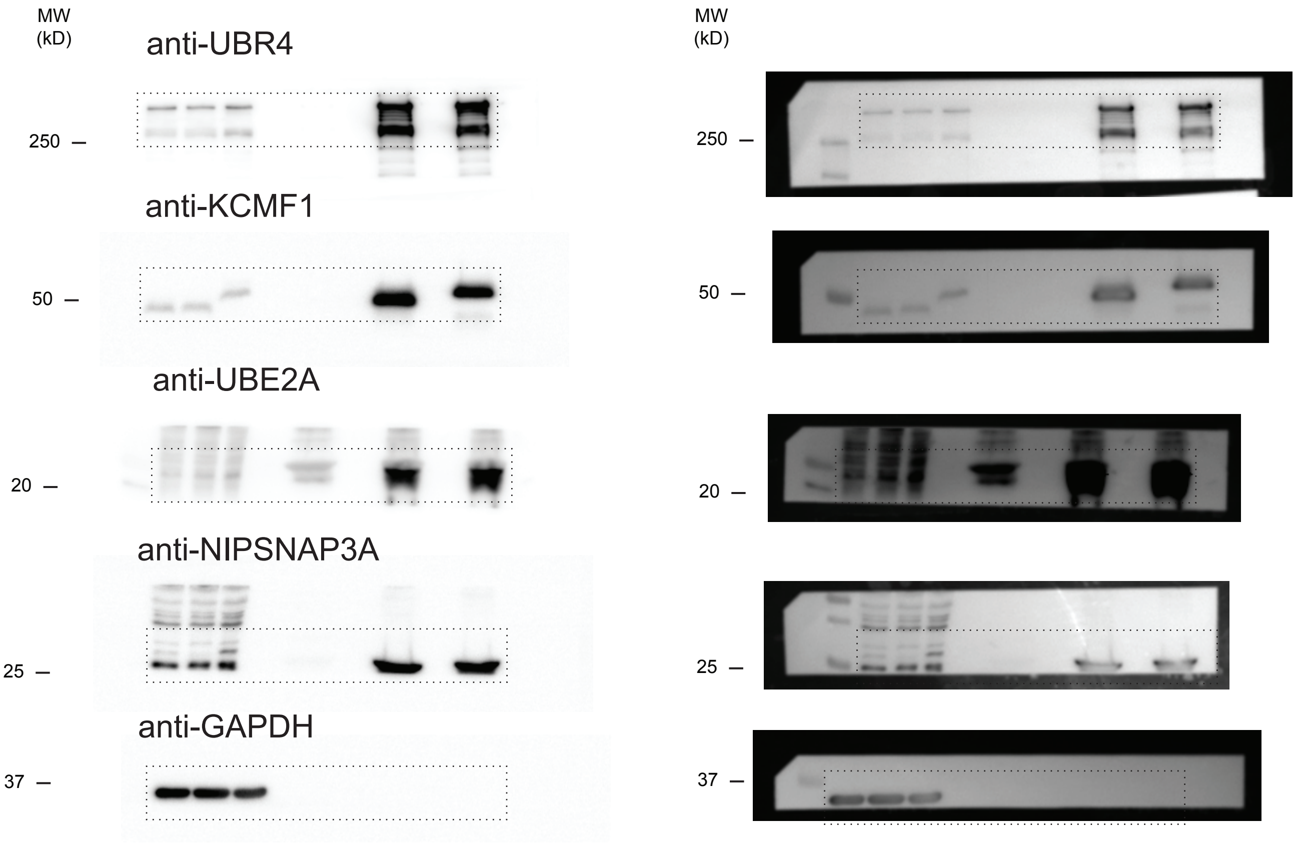

The loading control GAPDH was run on the same gel as UBR4 and KCMF1.

Chemiluminescence

Chemiluminescence + visible light

Extended Data Figure 2b

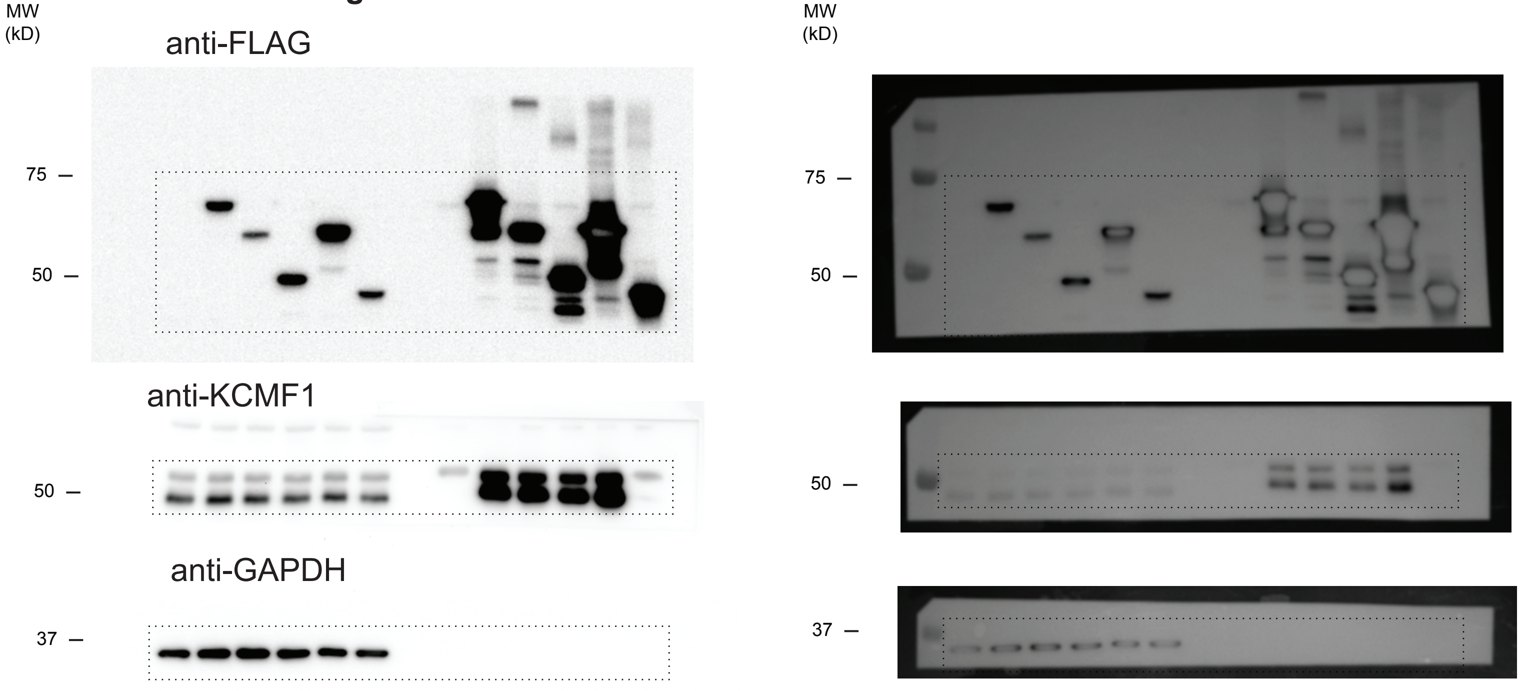

The loading control GAPDH was run on the same gel as KCMF1.

Extended Data Figure 2c

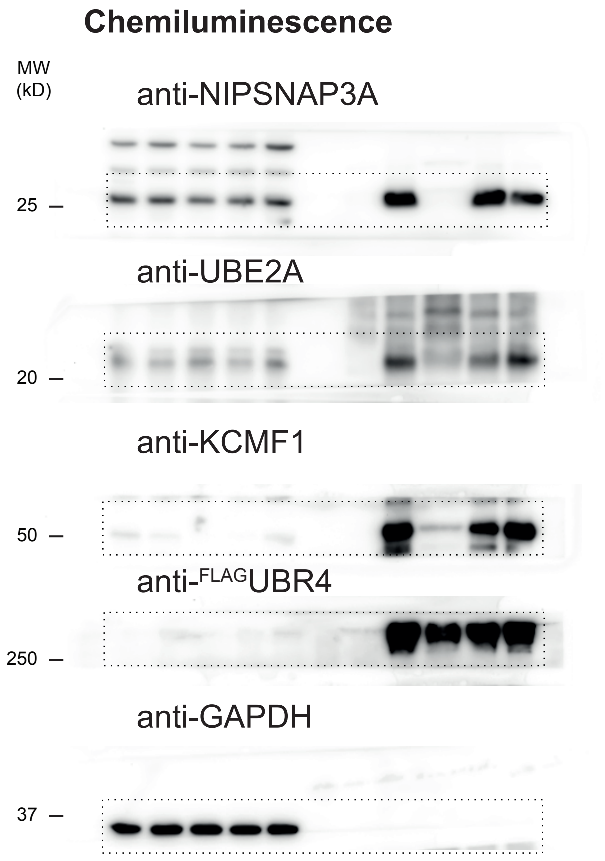

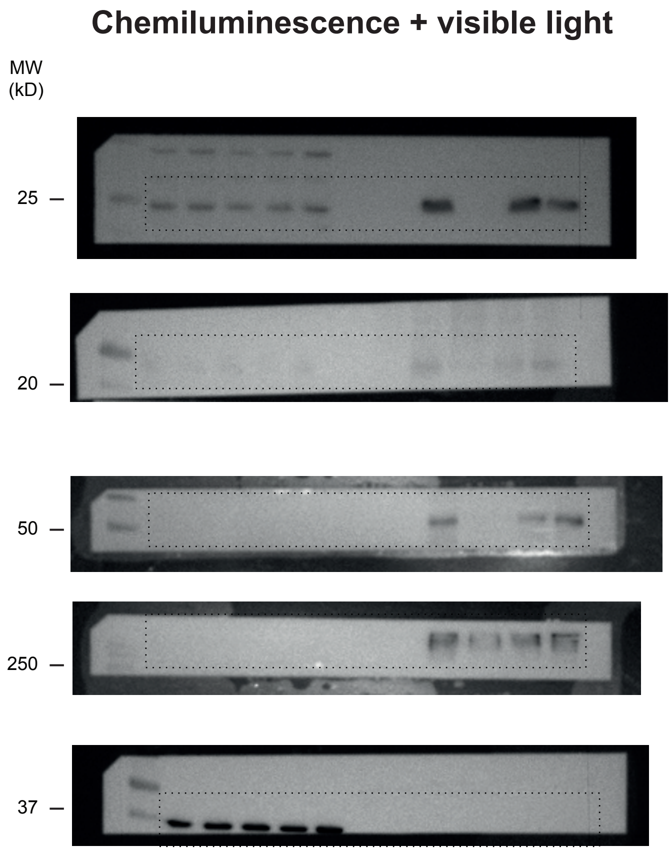

The loading control GAPDH was run on the same gel as UBE2A and FLAGUBR4.

Extended Data Figure 4a

Raw files

HRI-sumo/MBP-sumo autoradiography

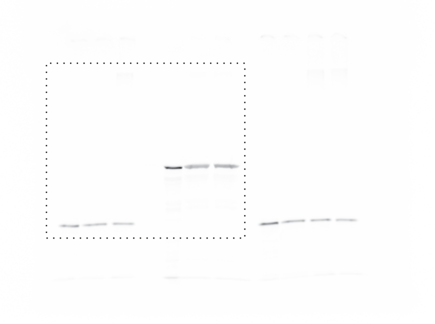

Contrasted raw files to discern typically low S^{35} autoradiography signal

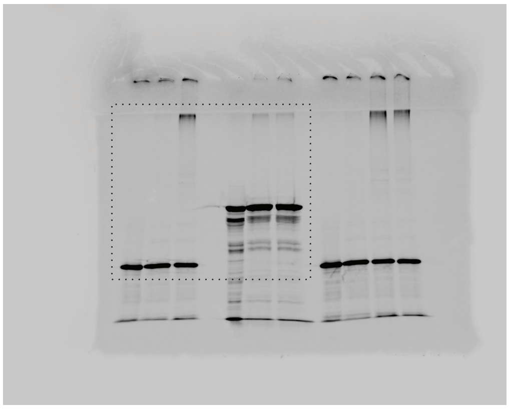

Extended Data Figure 4b

HRI-sumo autoradiography

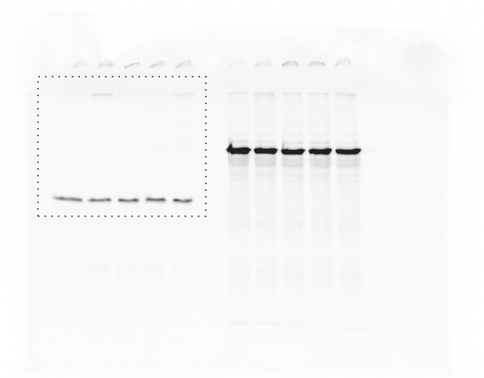

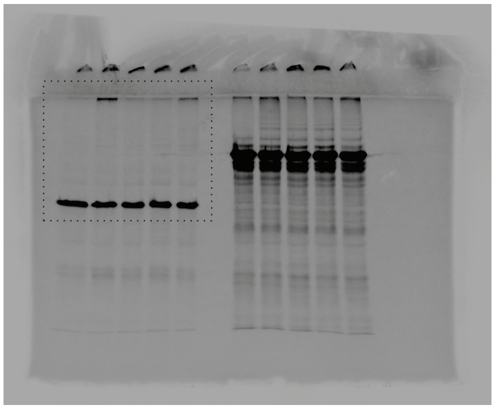

Extended Data Figure 4c

Chemiluminescence

Anti-FLAG Anti-Ub

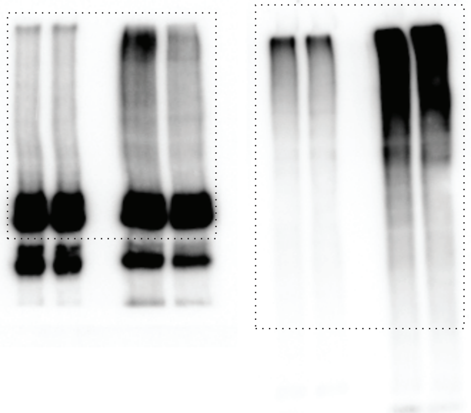

Chemiluminescence + visible light

Anti-FLAG

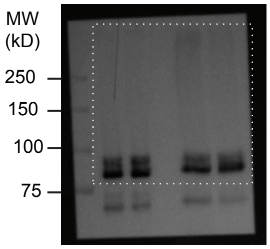

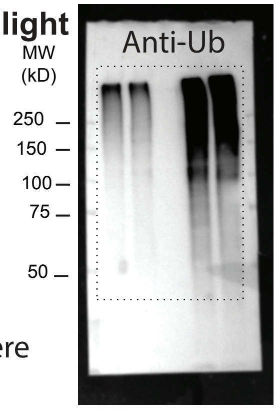

Ubiquitin and FLAG were run on different gels

Extended Data Figure 4d

Raw file

HRI-sumo autoradiography

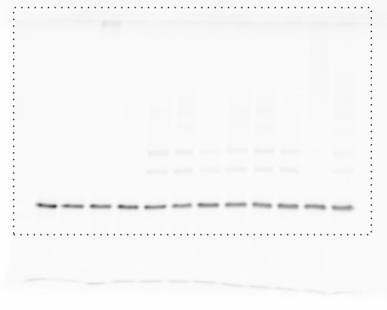

Contrasted raw file to discern typically low S^{35} autoradiography signal

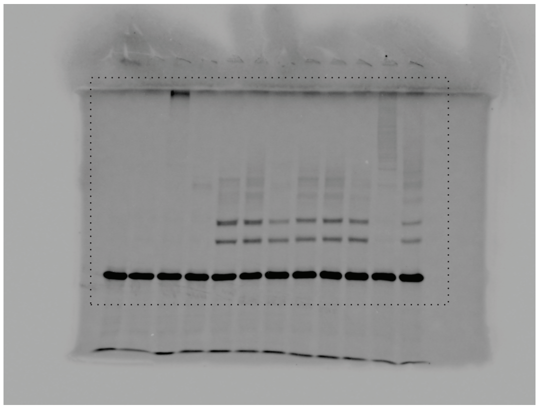

Extended Data Figure 5b

Chemiluminescence | Chemiluminescence + visible light

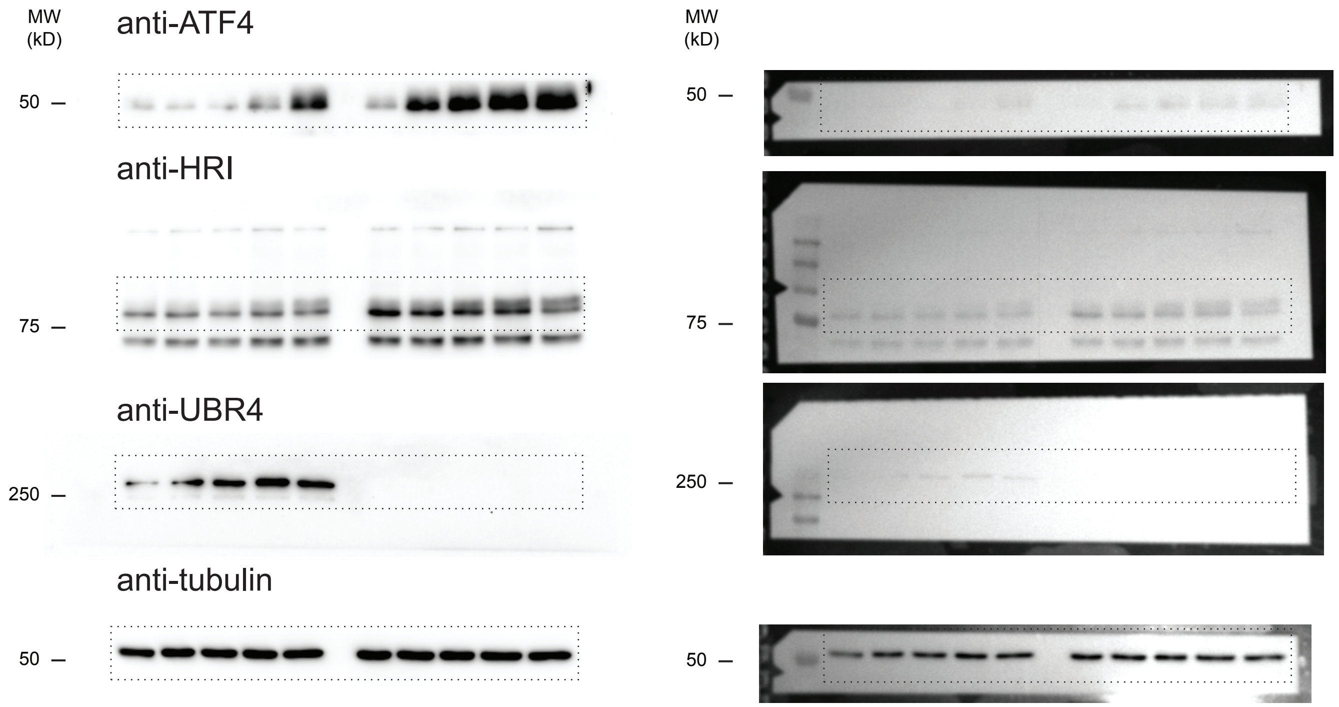

The loading control tubulin was run on the same gel as UBR4 .

Extended Data Figure 5c

Chemiluminescence | Chemiluminescence + visible light

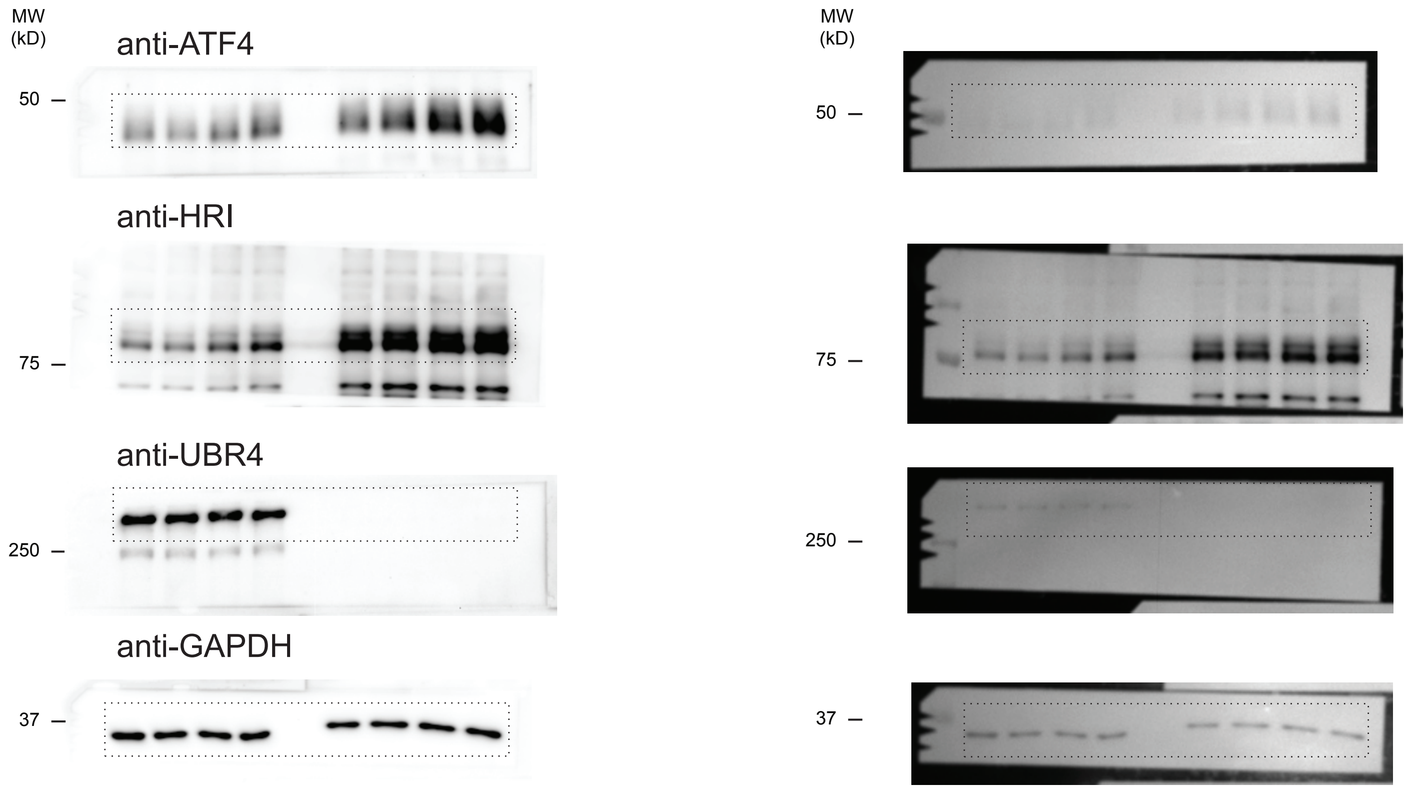

The loading control GAPDH was run on the same gel as ATF4, HRI and UBR4.

Extended Data Figure 5d

Chemiluminescence **Chemiluminescence + visible light**

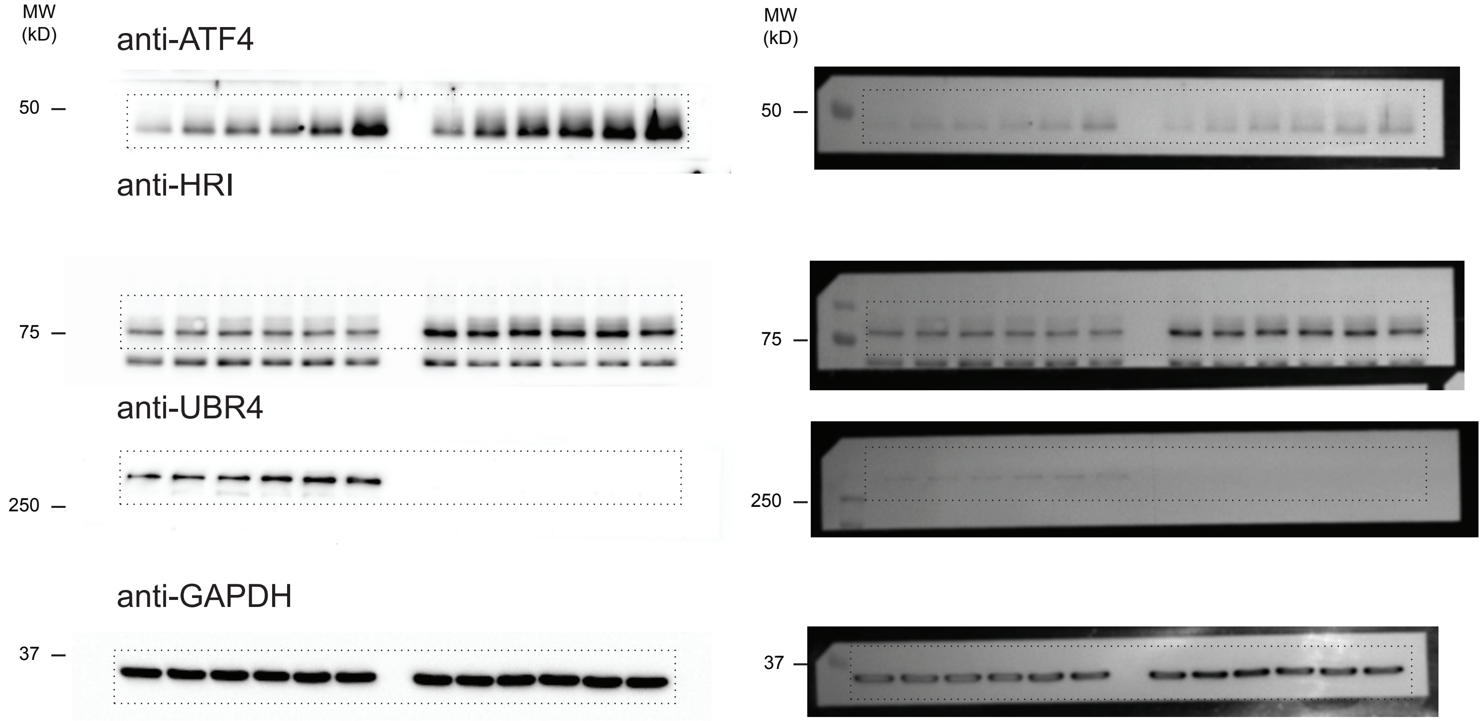

The loading control GAPDH was run on the same gel as ATF4, HRI and UBR4.

Extended Data Figure 5e

Chemiluminescence **Chemiluminescence + visible light**

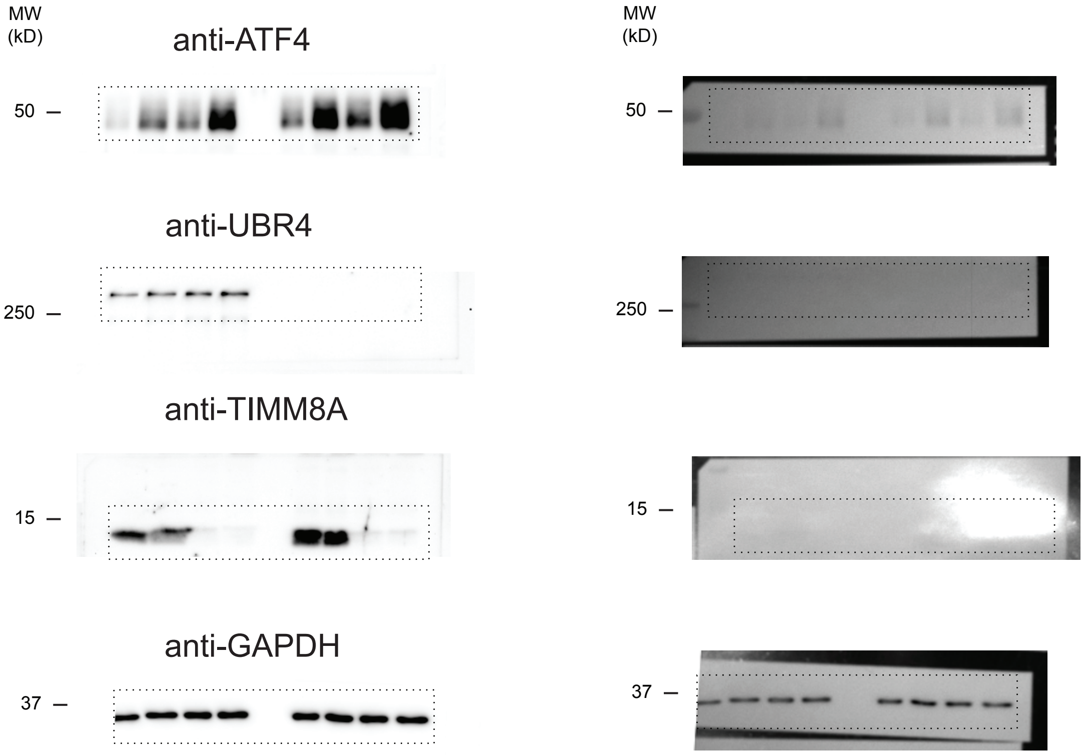

The loading control GAPDH was run on the same gel as ATF4 and UBR4.

Extended Data Figure 5h

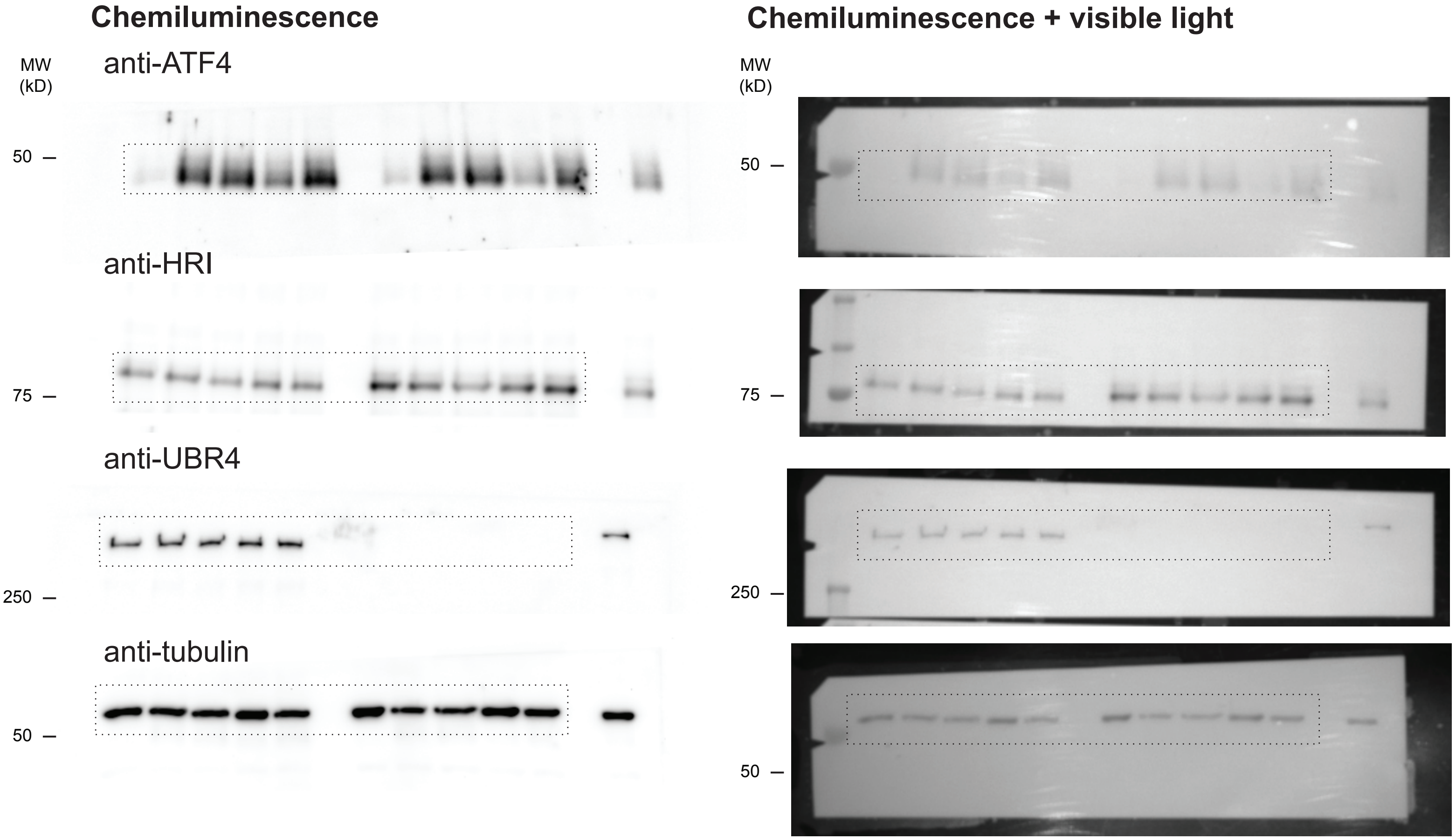

The loading control tubulin was obtained by stripping the ATF4 membrane, which was run on the same gel as HRI and UBR4.

Extended Data Figure 5i

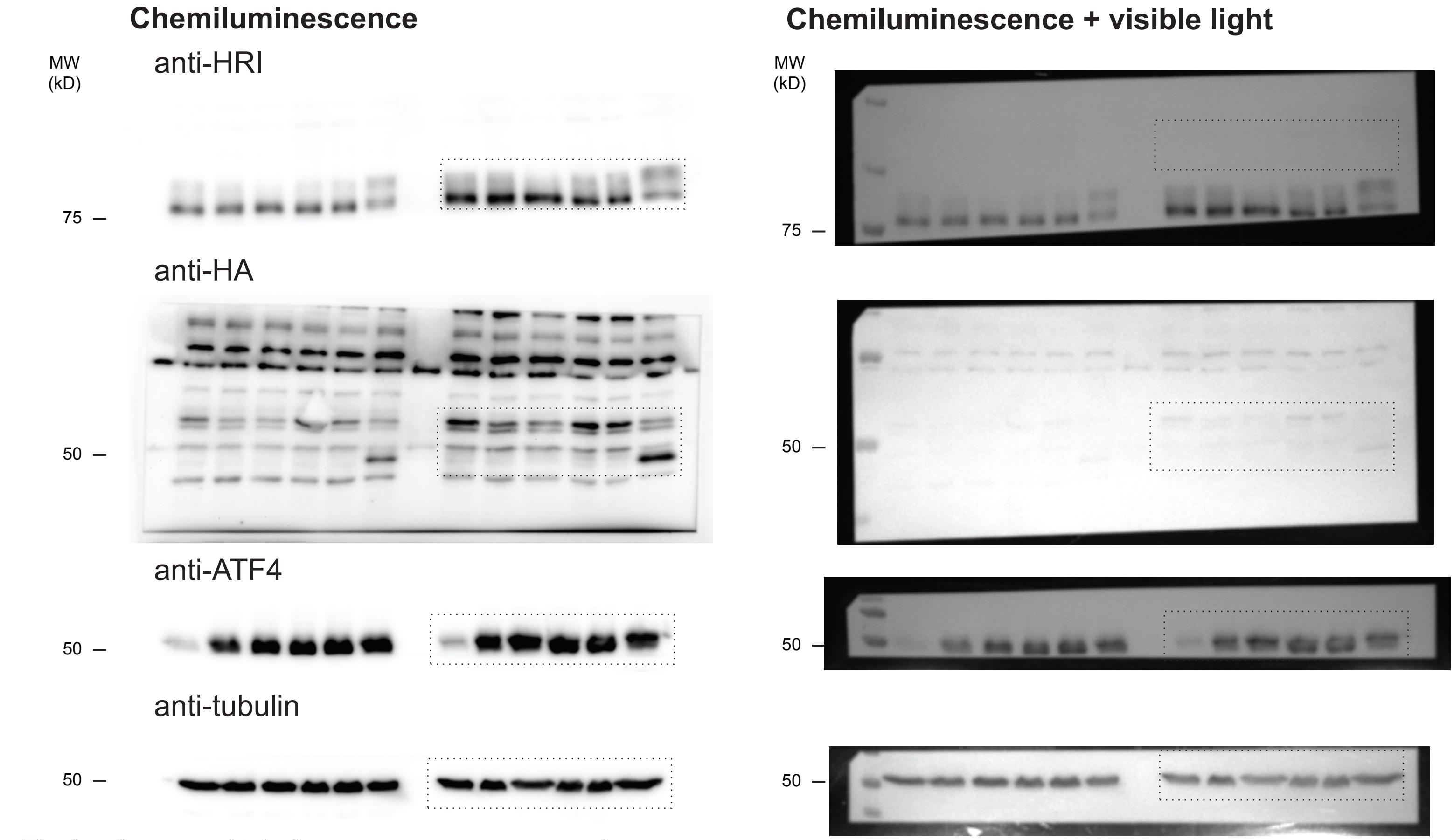

The loading control tubulin was run on a separate gel.

Extended Data Figure 6a

Chemiluminescence

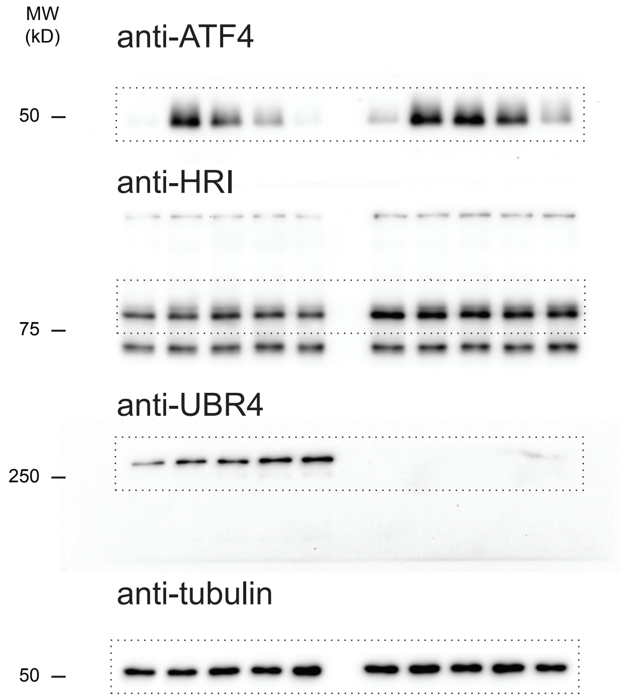

Chemiluminescence + visible light

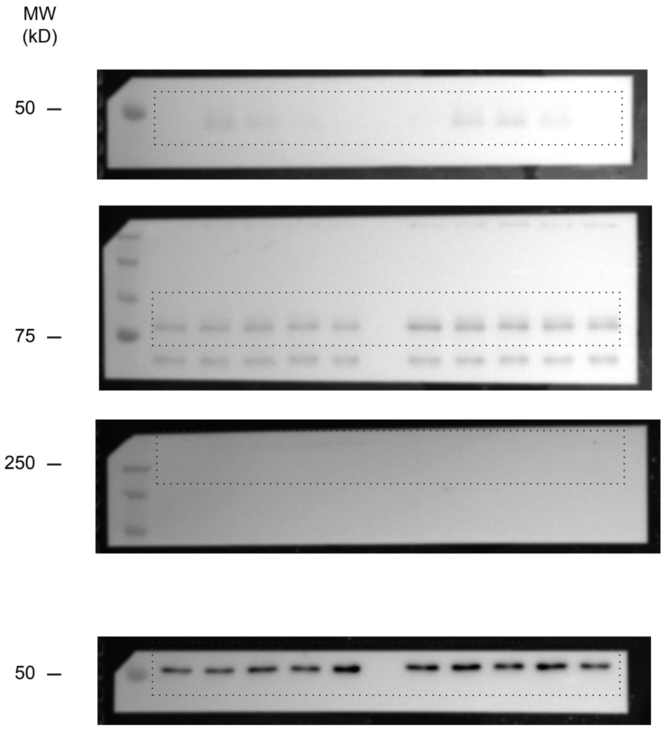

The loading control tubulin was run on the same gel as UBR4.

Extended Data Figure 6b

Chemiluminescence

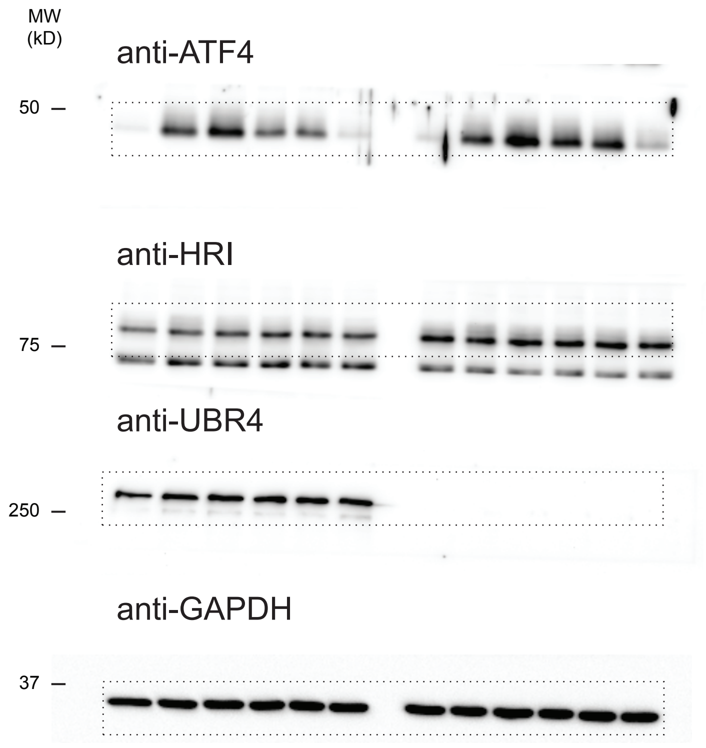

Chemiluminescence + visible light

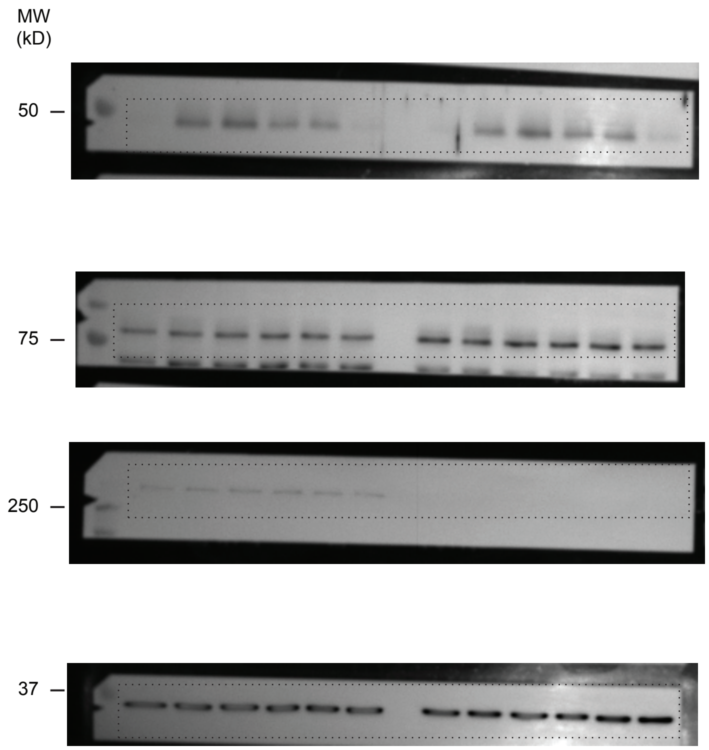

The loading control GAPDH was run on the same gel as ATF4, HRI and UBR4.

Extended Figure 6c

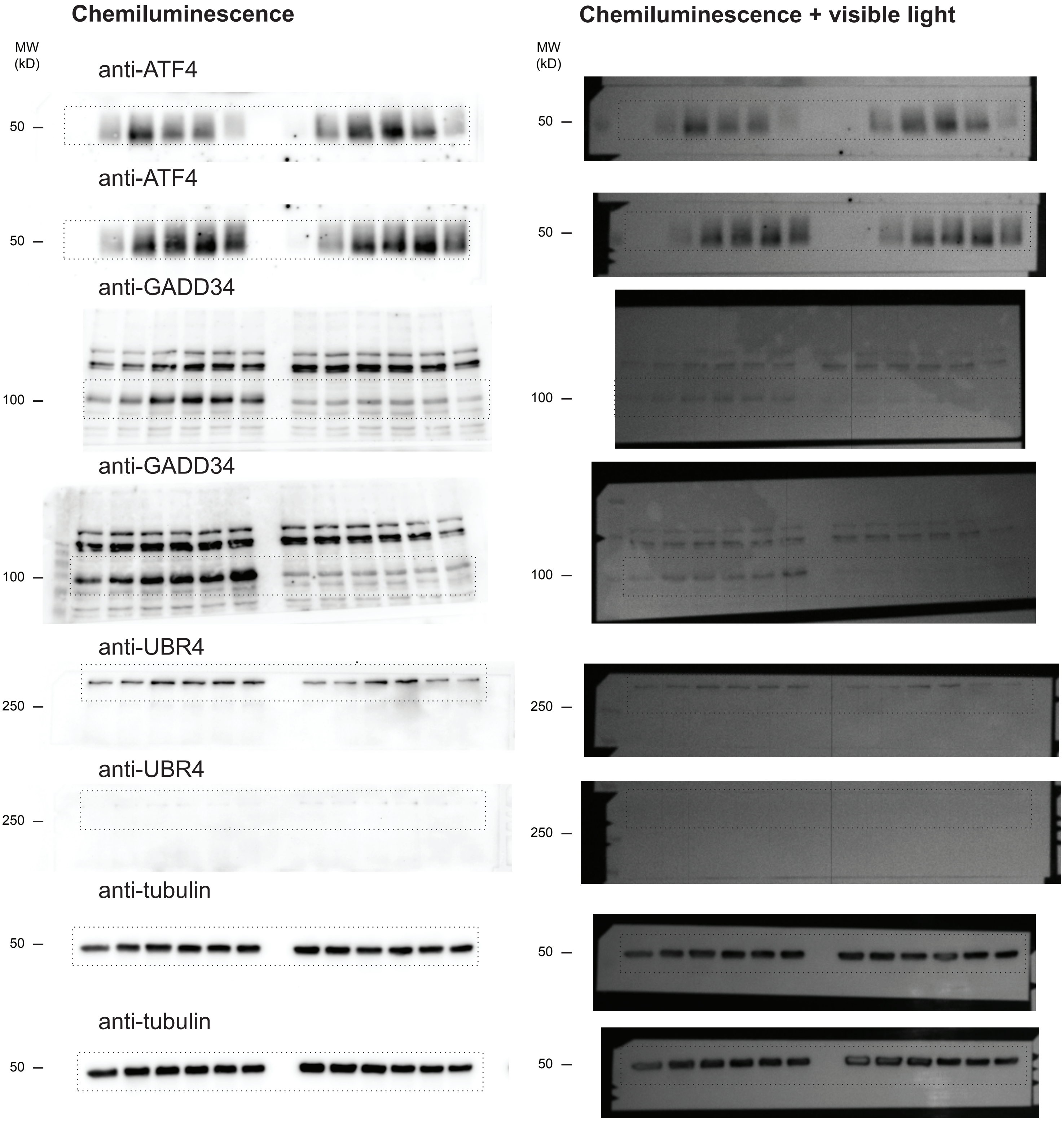

The loading controls tubulin were obtained by stripping the corresponding ATF4 membranes, which were run on the same gels as UBR4 in wt and *ΔUBR4* respectively.

Extended Data Figure 6d

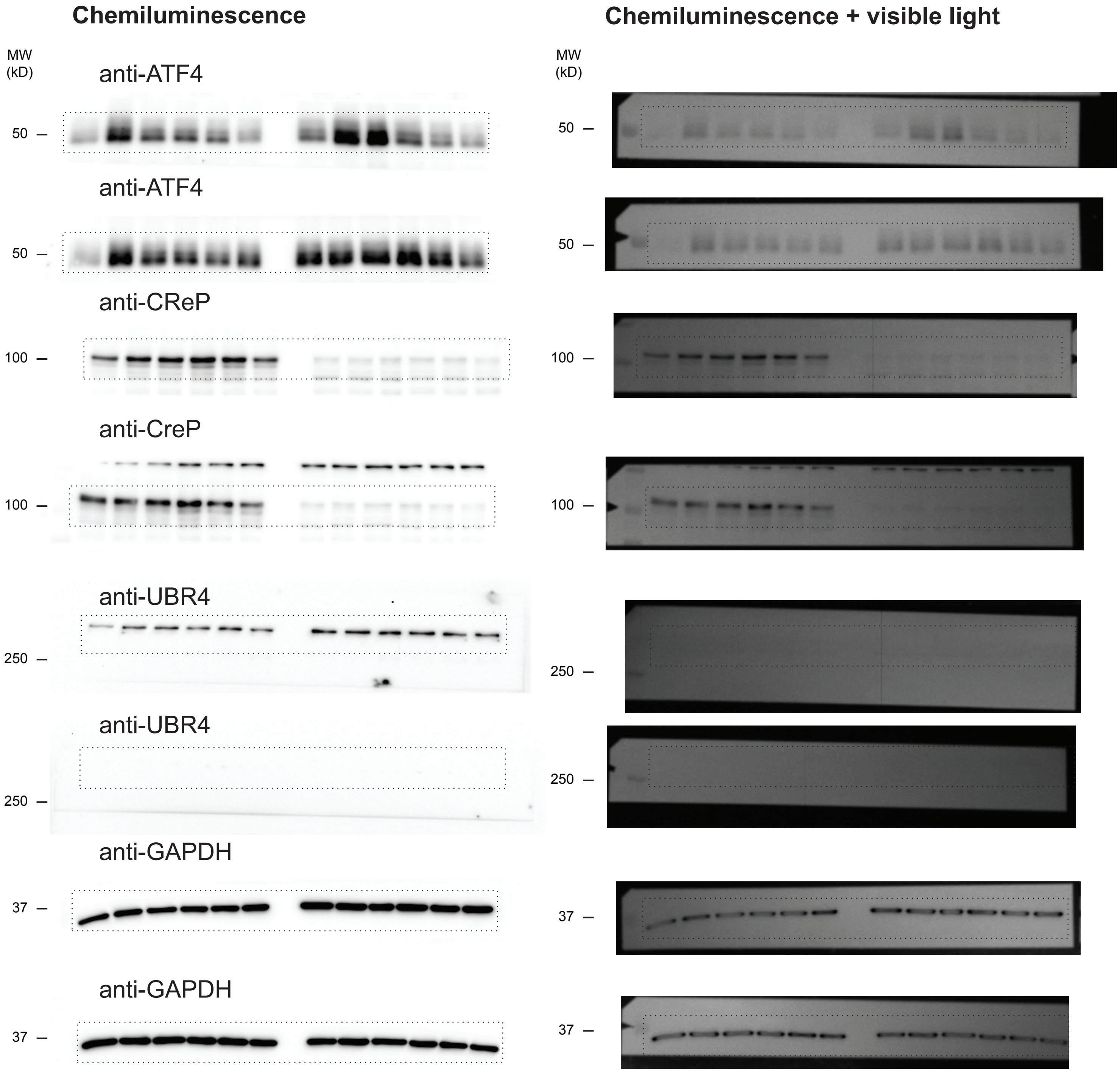

The loading controls GAPDH were run on the same gels as ATF4, CReP and UBR4 in wt and *ΔUBR4* respectively.

Extended Data Figure 6e

Chemiluminescence

Chemiluminescence + visible light

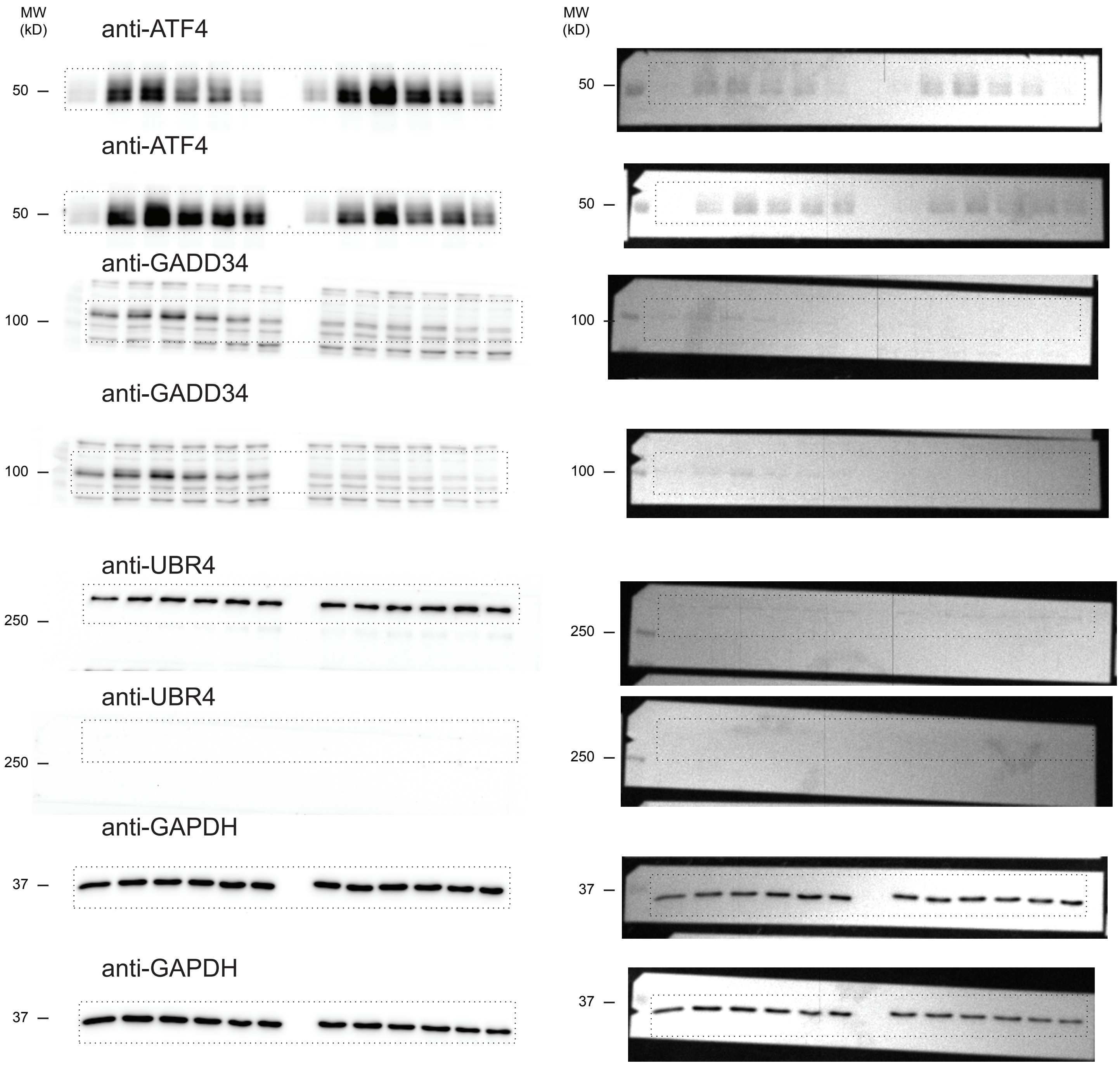

The loading controls GAPDH were run on the same gels as ATF4, GADD34 and UBR4 in wt and *ΔUBR4* respectively.

Extended Data Figure 7b

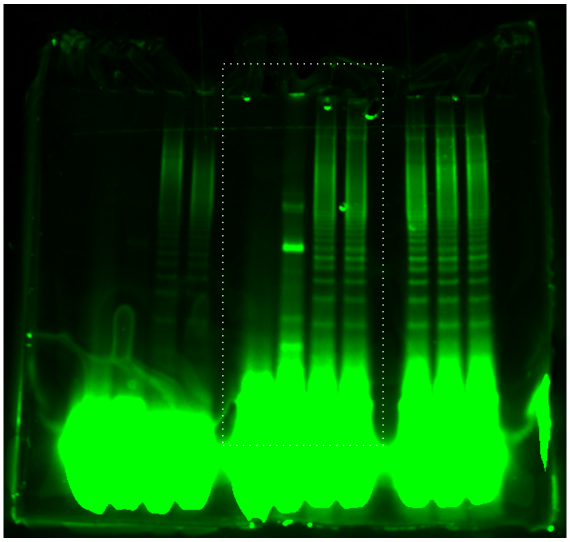

Extended Data Figure 7c

Raw file

HRI-sumo autoradiography

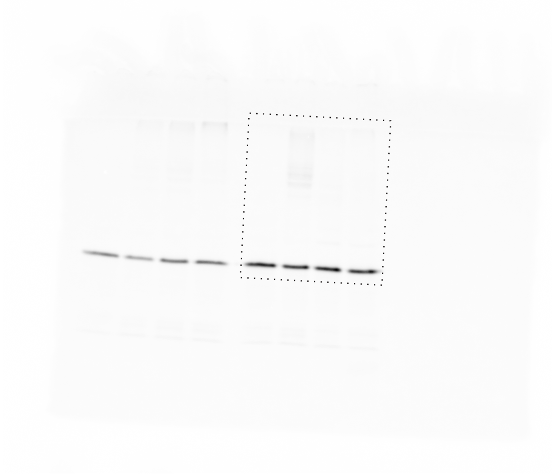

Contrasted raw file to discern typically low S^{35} autoradiography signal

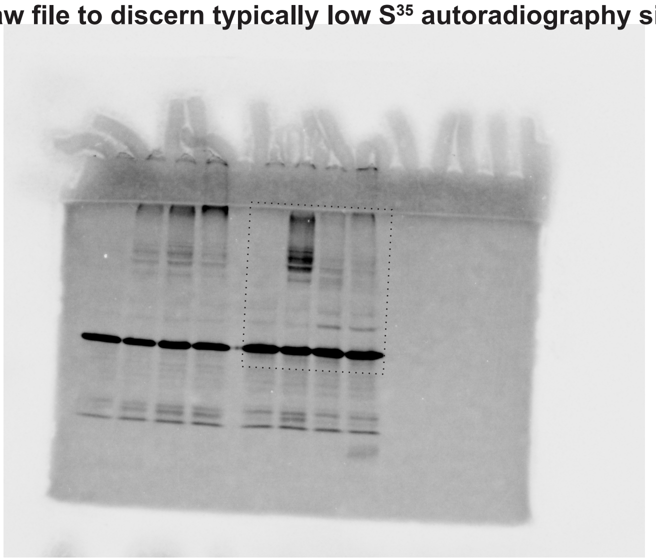

Extended Data Figure 7f

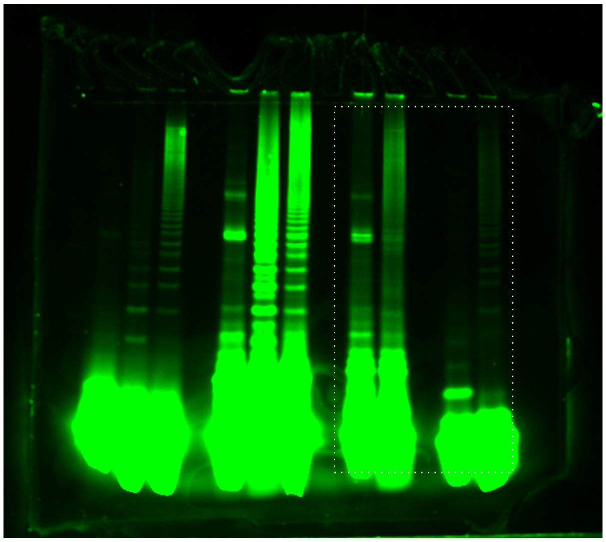

Extended Data Figure 8c

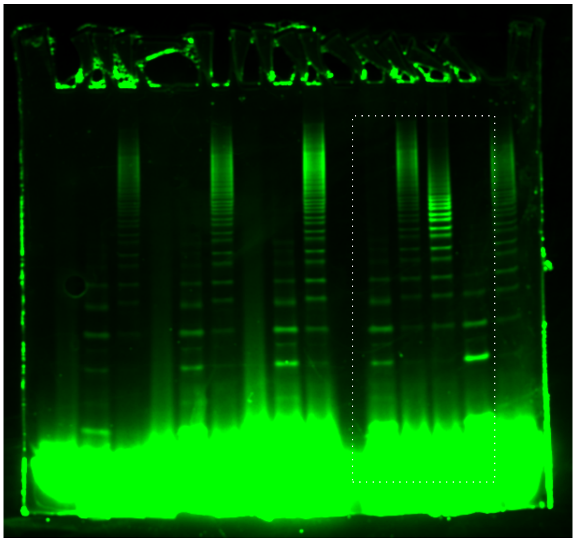

Extended Data Figure 8d

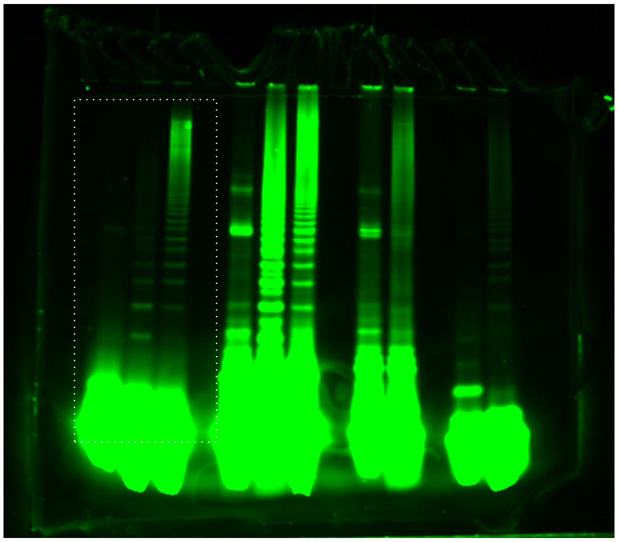

Extended Data Figure 8e

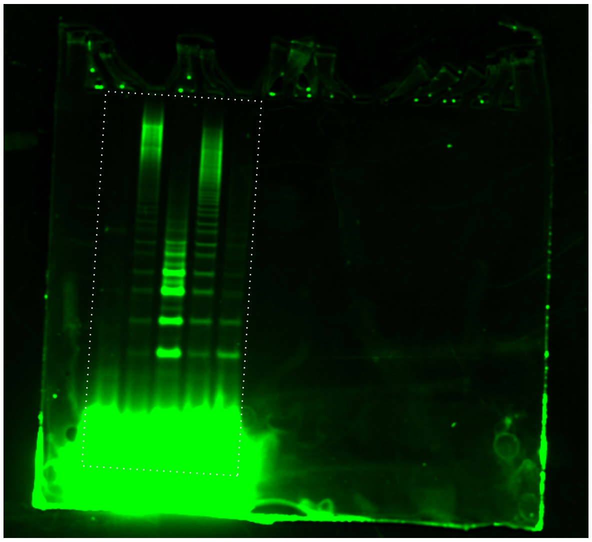

Extended Data Figure 8f

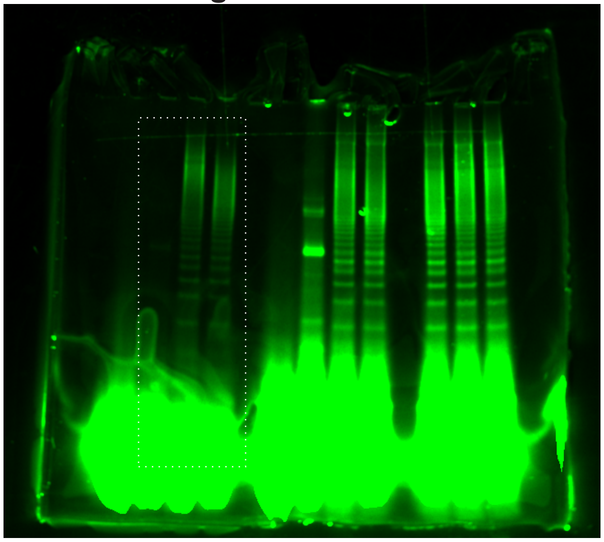

Extended Data Figure 8g

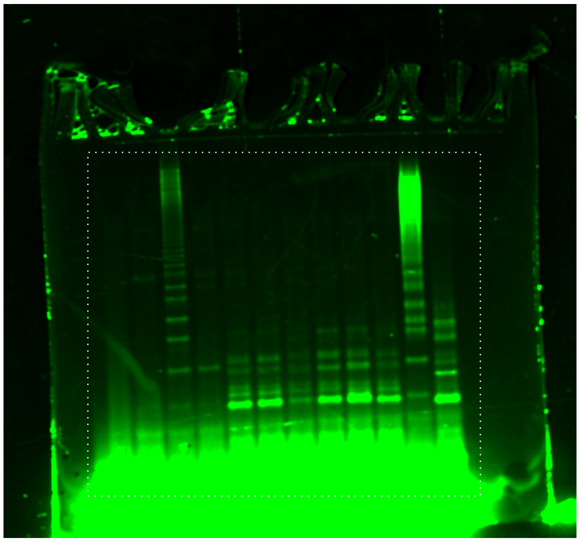

Extended Data Figure 8j

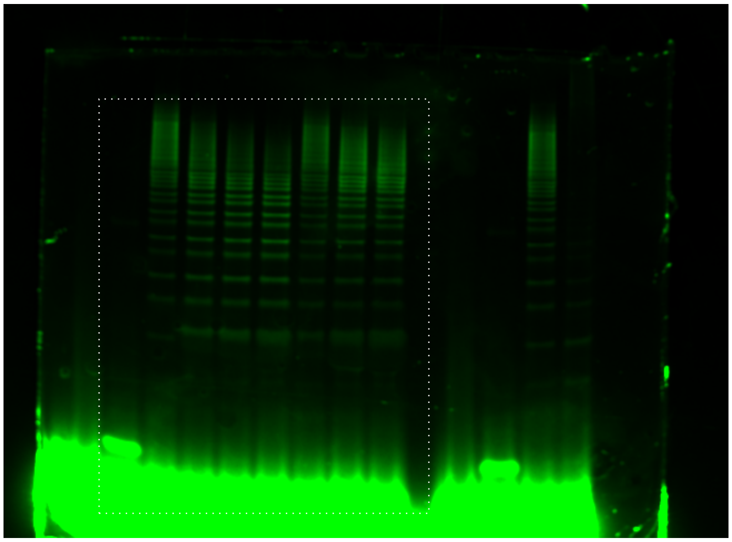

Extended Data Figure 8k

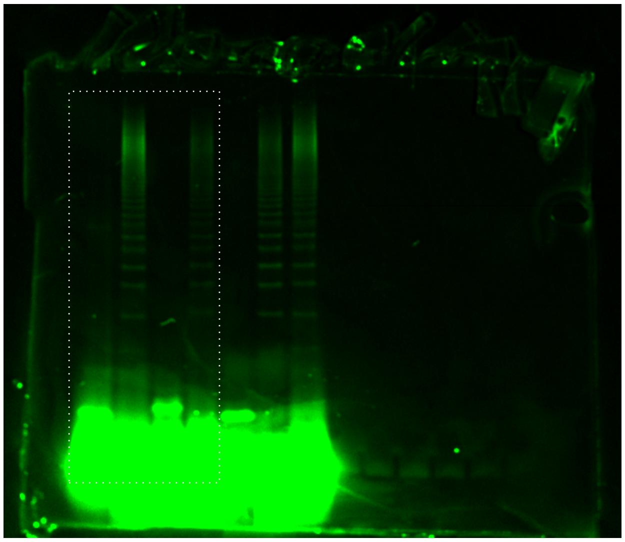

Extended Data Figure 8l

Chemiluminescence

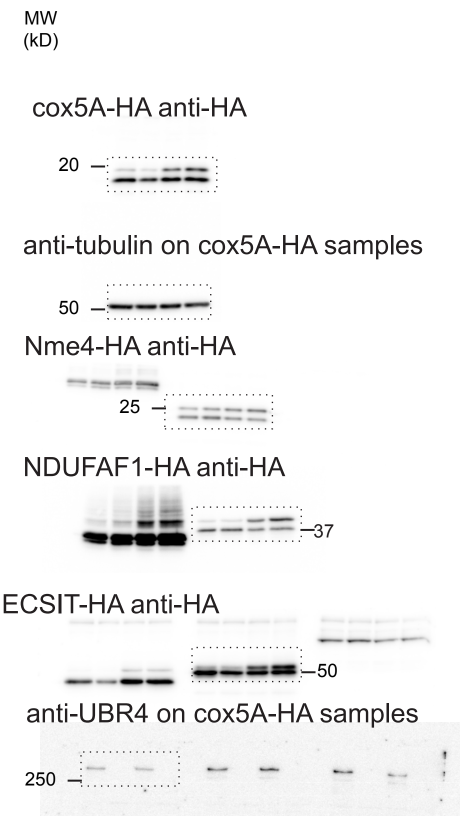

Chemiluminescence + visible light

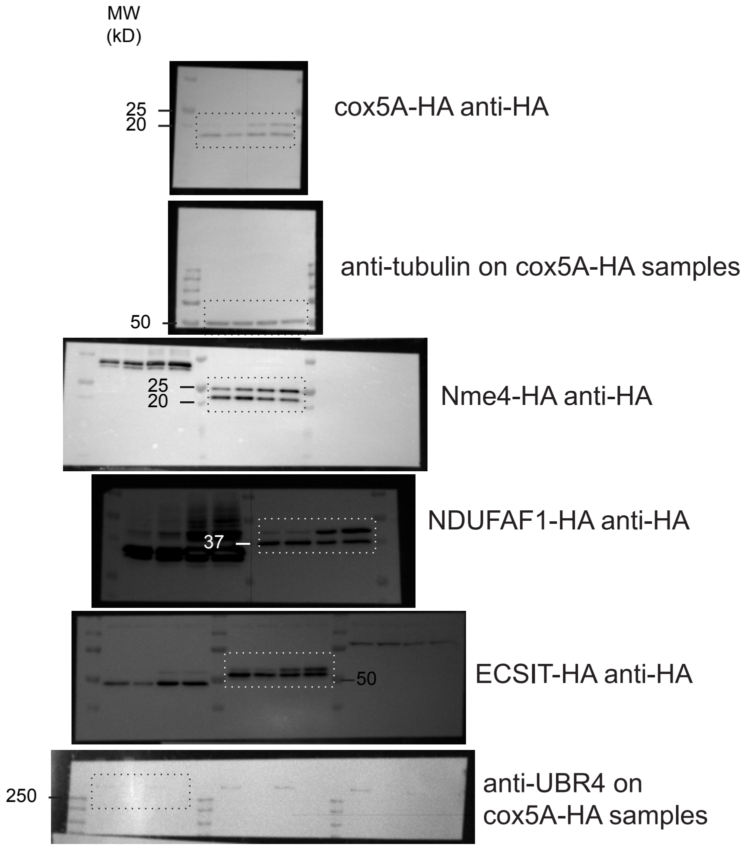

The loading control Tubulin was run on the same gel as cox5AHA.

Extended Data Figure 9a

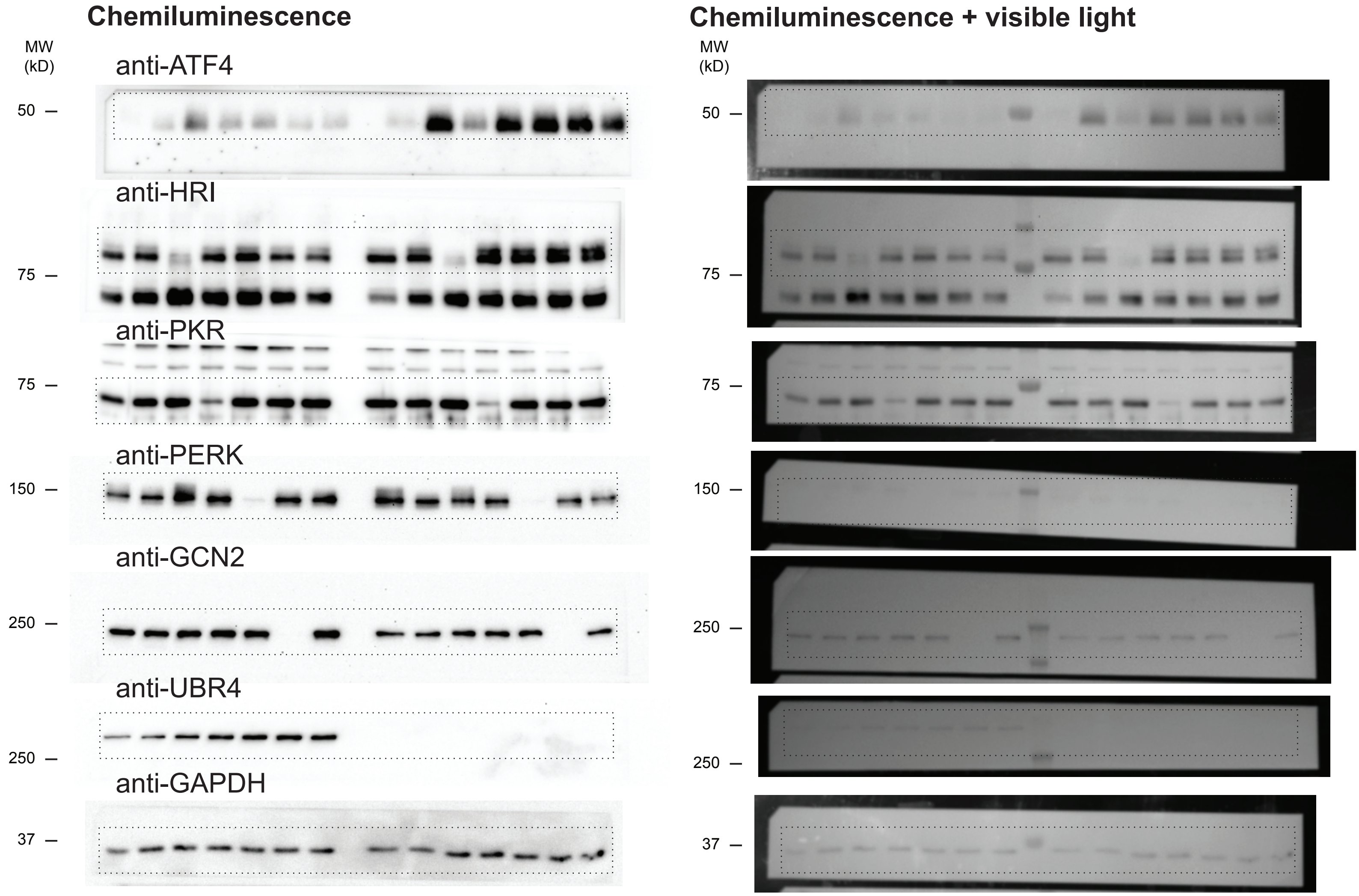

The loading control GAPDH was run on the same gel as PKR, PERK and UBR4.

Extended Data Figure 9b

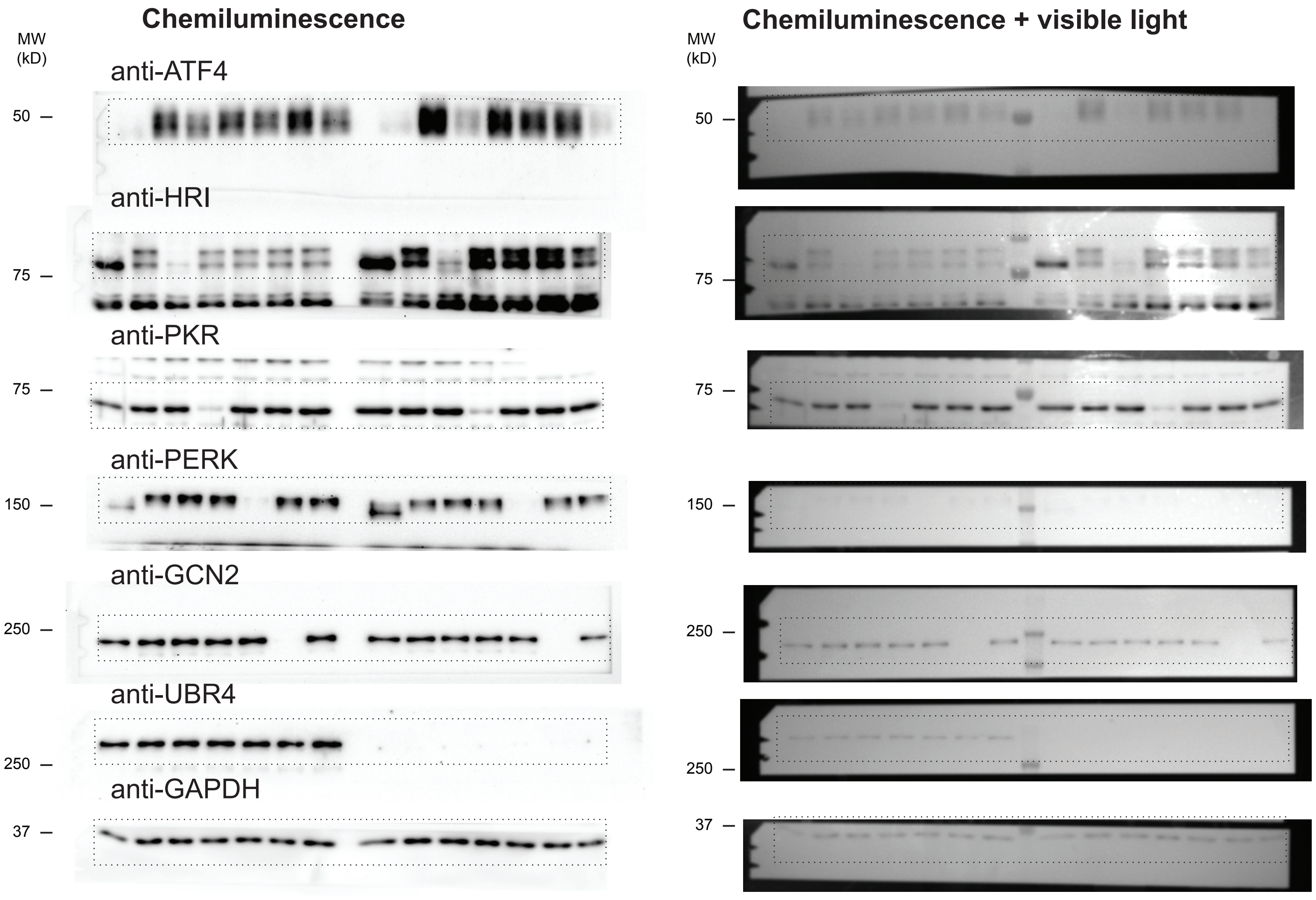

The loading control GAPDH was run on the same gel as PKR, PERK and UBR4.

Extended Data Figure 9c

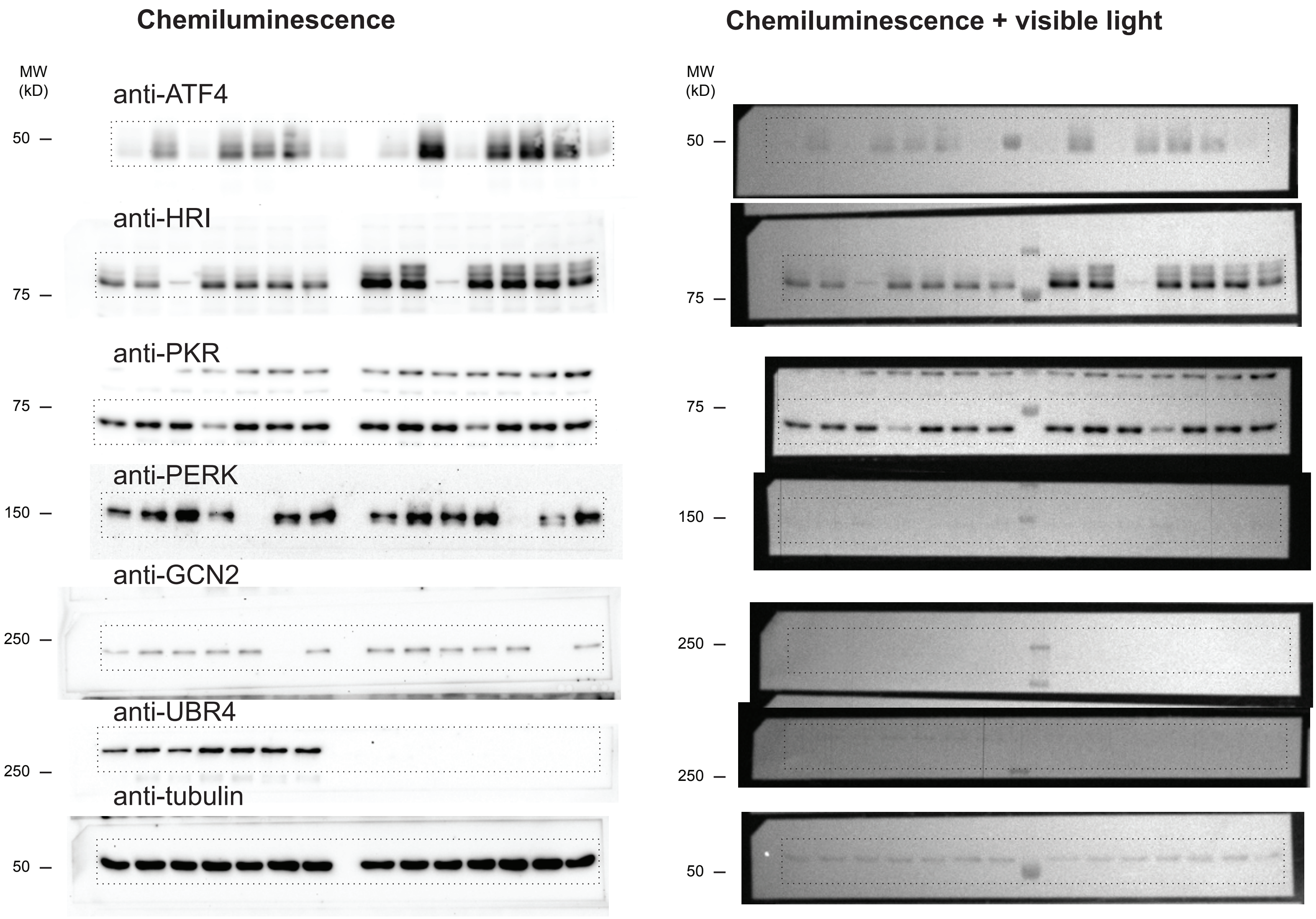

The loading control tubulin was run on the same gel as GCN2 and HRI.

Extended Data Figure 9d

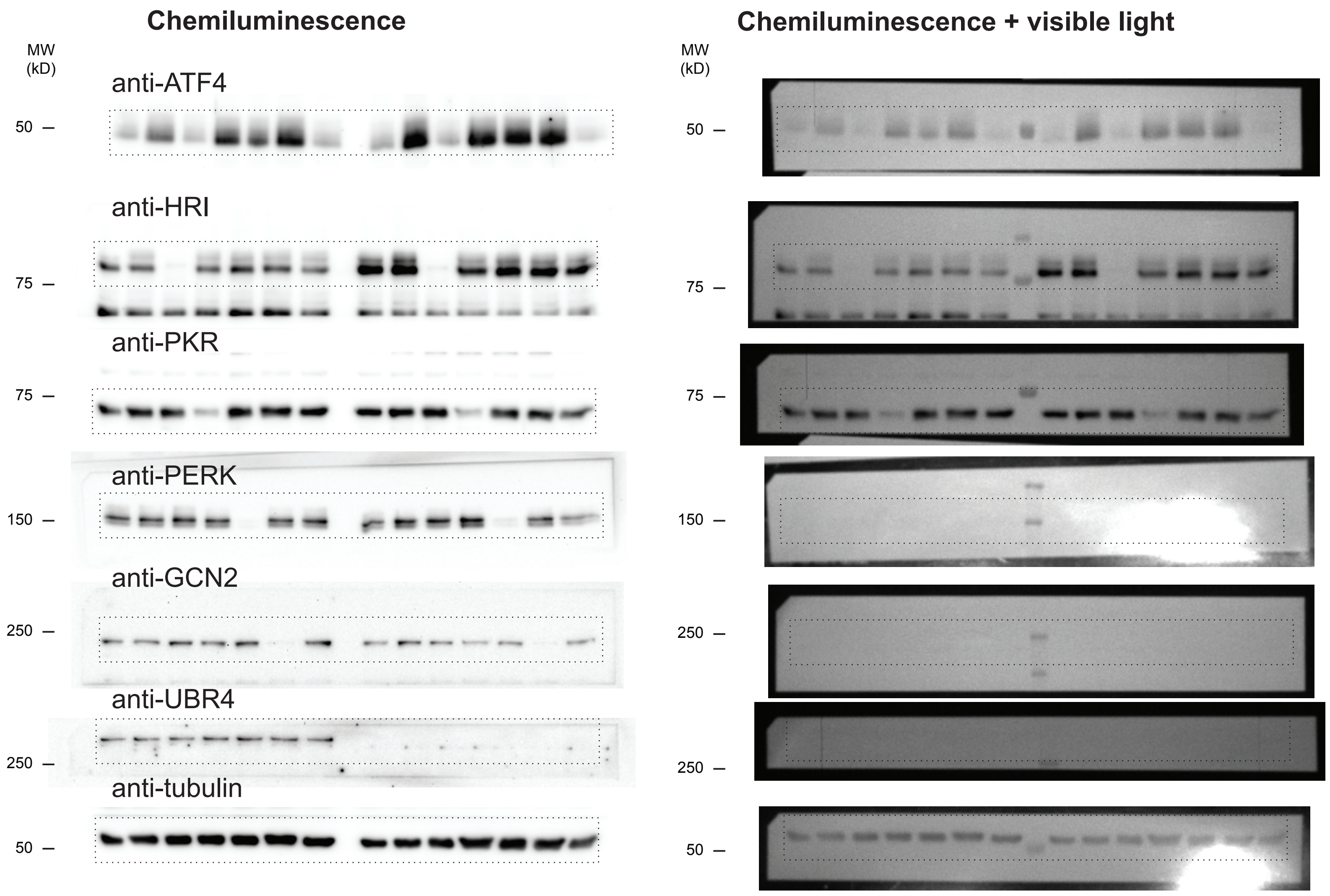

The loading control tubulin was run on the same gel as GCN2 and HRI.

Extended Data Figure 10a

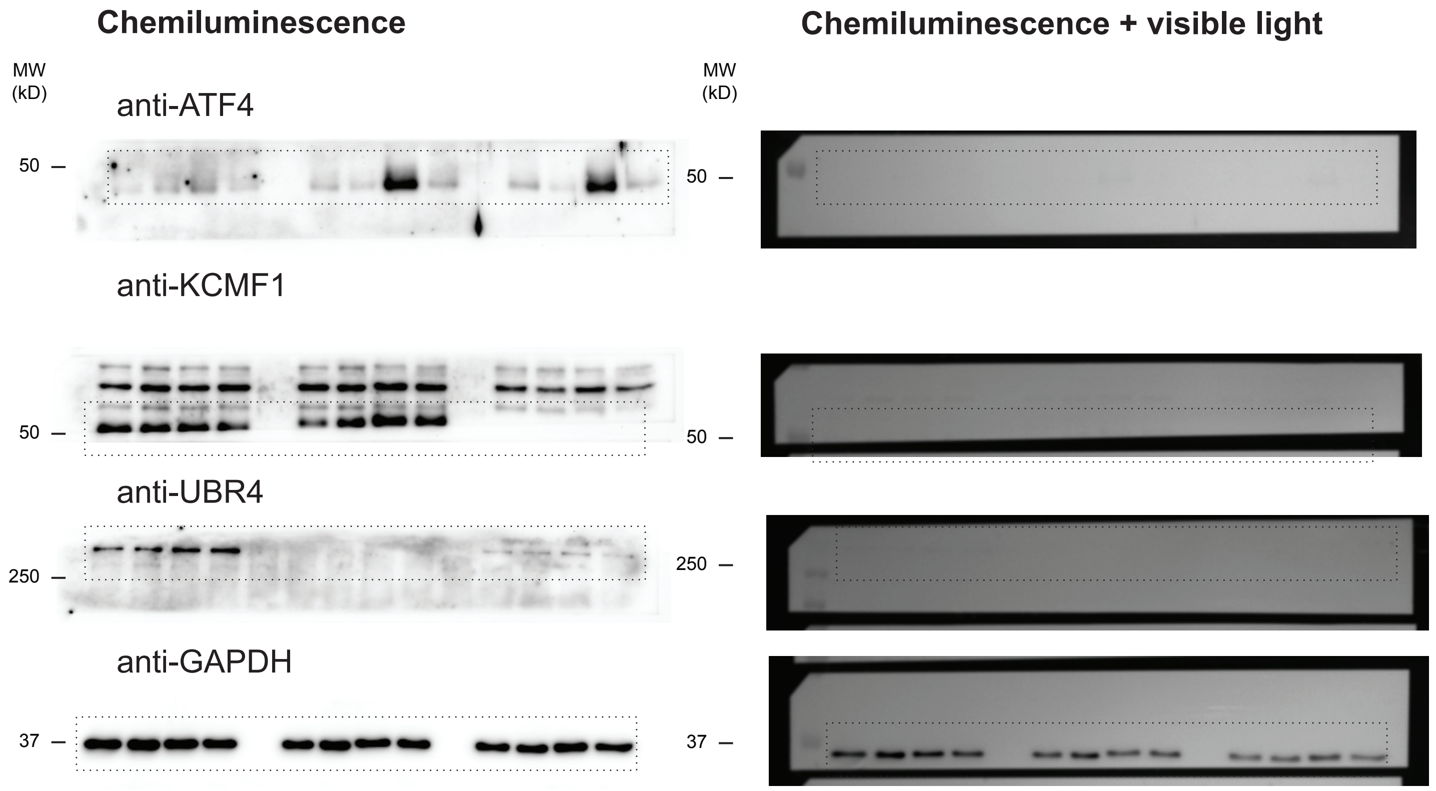

The loading control GAPDH was run on the same gel as UBR4 and KCMF1.

Extended Data Figure 10b

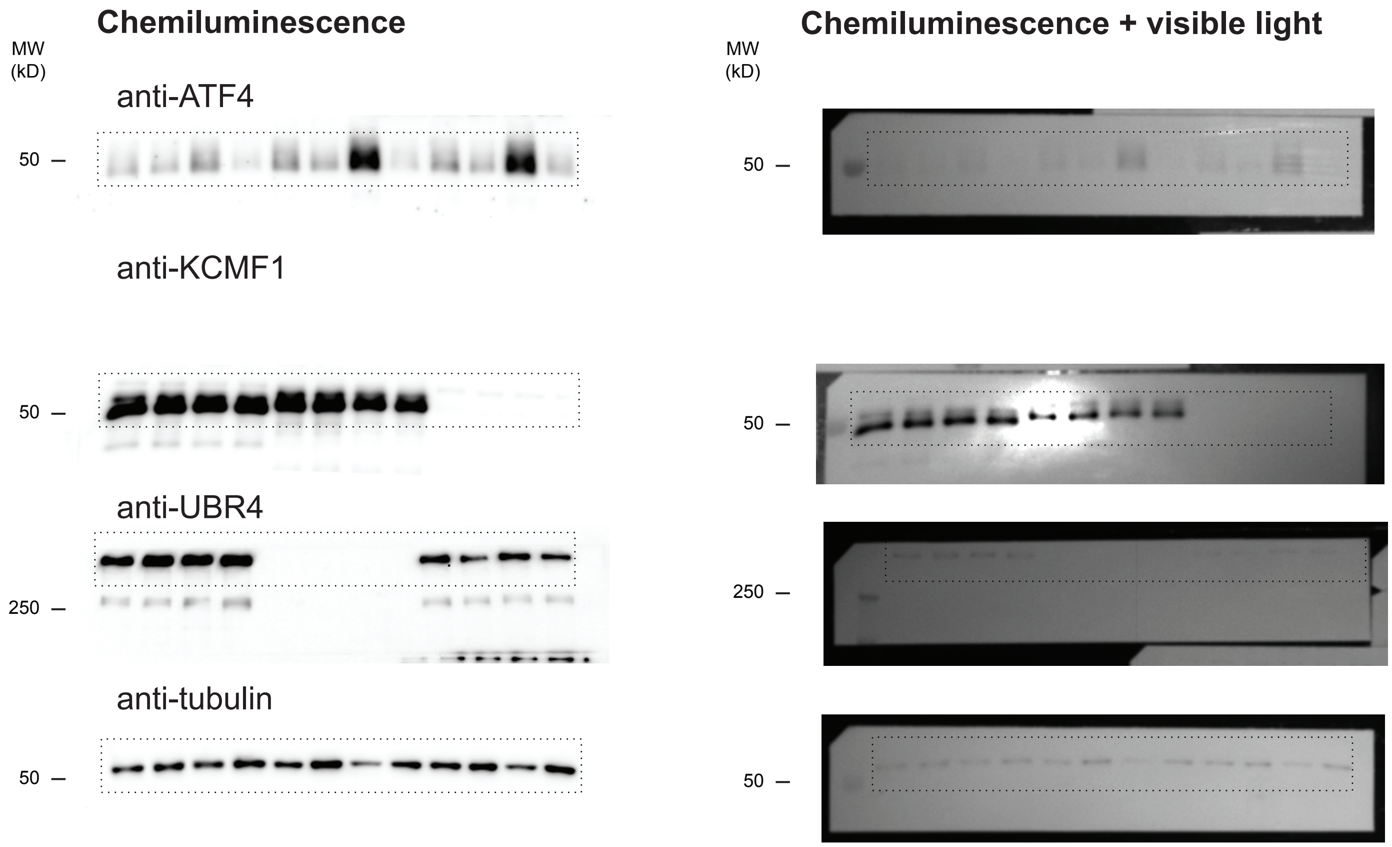

The loading control tubulin was obtained by stripping the ATF4 membrane, which was run on the same gel as UBR4.

Extended Data Figure 10c

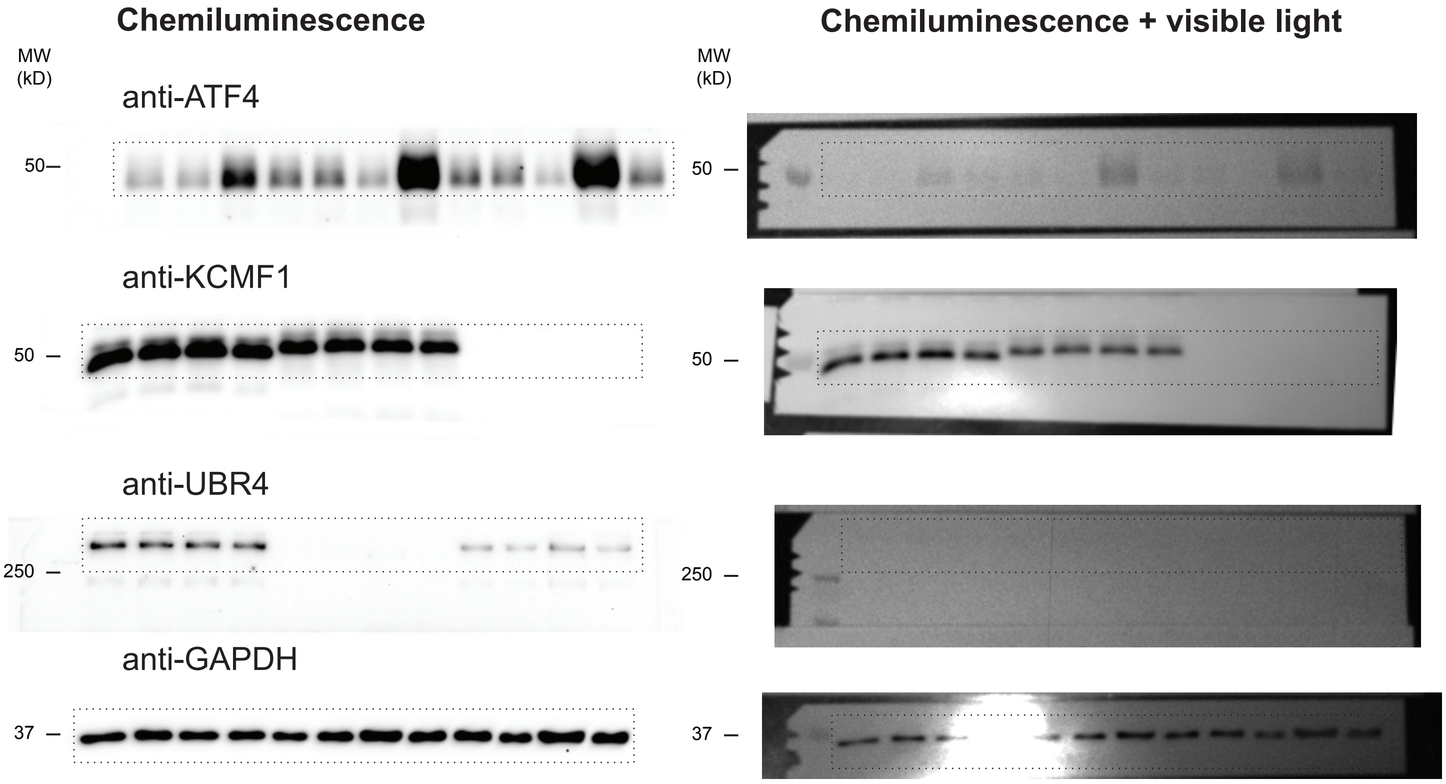

The loading control GAPDH was run on the same gel as UBR4 and ATF4.

Extended Data Figure 10e

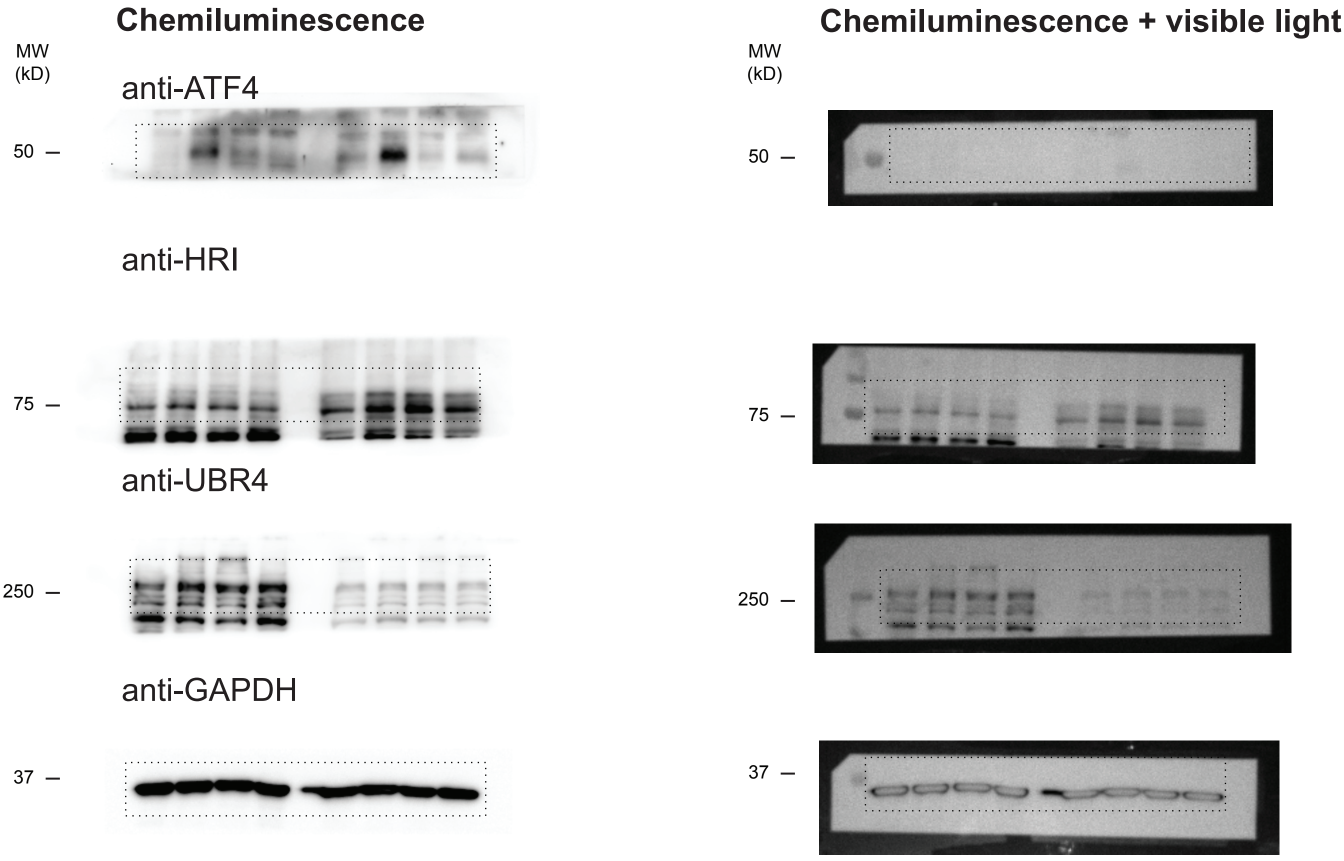

The loading control GAPDH was run on the same gel as UBR4, HRI and ATF4.

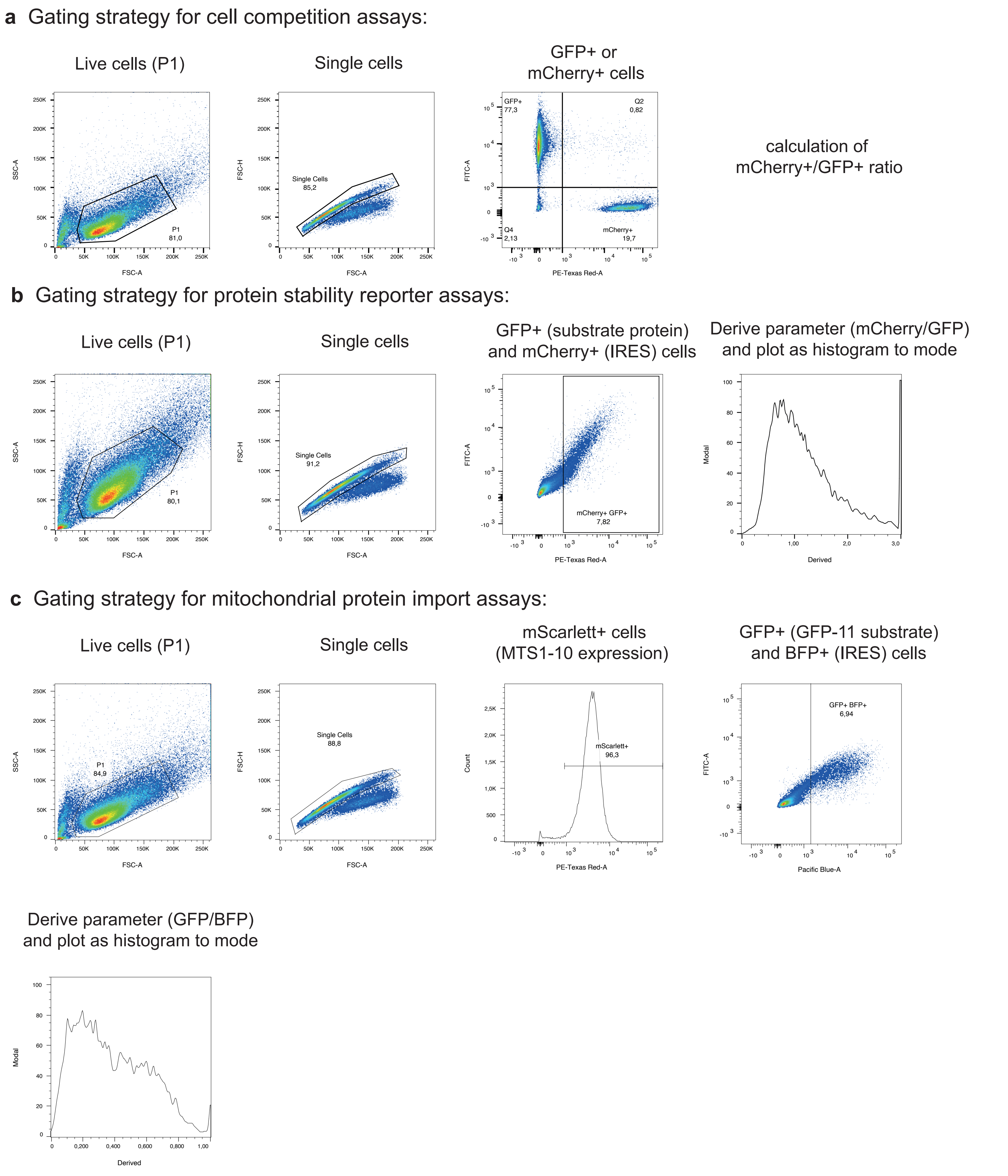

Supplementary Figure 2. Gating strategy for flow cytometry experiments. Representative plots shown for each type of flow cytometry analysis in this study. **a.** Gating strategy for cell competition assays as used in Fig. 1c, 1d, 1f, 5g, 5h and Extended Data Fig. 3e, 10f, 10g.
b. Gating strategy for protein stability reporter assays as used in Fig. 2b, 2c, 3a, 4a, 4c, 4f, 4j, 4k, 4l, 5a, 5e, 5f and Extended Data Fig. 3f, 3g, 3j, 3k, 4e, 5a, 5f, 5g, 6f, 6g, 7a, 7d, 7e, 7g, 8j, 8k, 8m, 8n, 8o, 8p.
c. Gating strategy for mitochondrial protein import assays as used in Fig. 2a, 5c and Extended Data Fig. 3a, 3b, 3c, 9e, 10d.
