# Supplementary material for: Stress response silencing by an E3 ligase mutated in neurodegeneration
Source: Nature. 2024 Jan 31;626(8000):874–80. doi: 10.1038/s41586-023-06985-7 (PMC10881396; doi:10.1038/s41586-023-06985-7)
Supplement: Supplementary file 2 — Reporting Summary [file 41586_2023_6985_MOESM2_ESM.pdf]

nature portfolio

Corresponding author(s): Michael Rape

Last updated by author(s): 01/10/2024

Reporting Summary

Nature Portfolio wishes to improve the reproducibility of the work that we publish. This form provides structure for consistency and transparency in reporting. For further information on Nature Portfolio policies, see our Editorial Policies and the Editorial Policy Checklist.

Statistics

For all statistical analyses, confirm that the following items are present in the figure legend, table legend, main text, or Methods section.

n/a	Confirmed	
☐	☒	The exact sample size (n) for each experimental group/condition, given as a discrete number and unit of measurement
☐	☒	A statement on whether measurements were taken from distinct samples or whether the same sample was measured repeatedly
☐	☒	The statistical test(s) used AND whether they are one- or two-sided<br>*Only common tests should be described solely by name; describe more complex techniques in the Methods section.*
☒	☐	A description of all covariates tested
☒	☐	A description of any assumptions or corrections, such as tests of normality and adjustment for multiple comparisons
☐	☒	A full description of the statistical parameters including central tendency (e.g. means) or other basic estimates (e.g. regression coefficient) AND variation (e.g. standard deviation) or associated estimates of uncertainty (e.g. confidence intervals)
☐	☒	For null hypothesis testing, the test statistic (e.g. F, t, r) with confidence intervals, effect sizes, degrees of freedom and P value noted<br>*Give P values as exact values whenever suitable.*
☒	☐	For Bayesian analysis, information on the choice of priors and Markov chain Monte Carlo settings
☒	☐	For hierarchical and complex designs, identification of the appropriate level for tests and full reporting of outcomes
☒	☐	Estimates of effect sizes (e.g. Cohen's d, Pearson's r), indicating how they were calculated

Our web collection on statistics for biologists contains articles on many of the points above.

Software and code

Policy information about availability of computer code

Data collection	All software used is freely/comercially available: FACSDiva (Version 9.0)
Data analysis	All software used is freely/comercially available: FlowJo (Version 10.8.1), GraphPad Prism (Version 9), ImageJ2 (Version 2.9.0/1.53t), Cytoscape ClueGO (v3.7.1), CasTLE (v1.0), Kallisto (v.0.48.0), DESeq2 (Galaxy Version 2.11.40.7), Cluster 3.0, Java TreeView (v.1.1.6r4).

For manuscripts utilizing custom algorithms or software that are central to the research but not yet described in published literature, software must be made available to editors and reviewers. We strongly encourage code deposition in a community repository (e.g. GitHub). See the Nature Portfolio guidelines for submitting code & software for further information.

Data

Policy information about availability of data

All manuscripts must include a data availability statement. This statement should provide the following information, where applicable:

- Accession codes, unique identifiers, or web links for publicly available datasets
- A description of any restrictions on data availability
- For clinical datasets or third party data, please ensure that the statement adheres to our policy

Source data for immunoblots are provided in Supplementary Fig. 1. Gating strategies for flow cytometry experiments are provided in Supplementary Fig. 2. Source data for the CRISPR screen are provided in Supplementary Table 1. Immunoprecipitation and mass spectrometry source data (associated with Fig. 1e and Extended Data Fig. 2d) are provided in Supplementary Table 2. RNA-seq data (associated with Fig. 3 and Extended Data Fig. 3h) have been deposited into the GEO (accession

number GSE232191). Source data for this RNA-seq analysis are also provided in Supplementary Table 3. The human reference transcriptome (GRCh38, Ensembl Release 96), which was used to align the RNA-seq data can be accessed at Ensembl (http://apr2019.archive.ensembl.org/Homo_sapiens/Info/Index). The previously published RNA-seq data of HEK293T WT sgCNTRL cells and sgHRI cells treated with oligomycin[23] can be accessed at the GEO (accession number GSE134986). There are no restrictions on data availability.

Research involving human participants, their data, or biological material

Policy information about studies with human participants or human data. See also policy information about sex, gender (identity/presentation), and sexual orientation and race, ethnicity and racism.

Reporting on sex and gender	This study does not involve human participants, their data or their biological material
Reporting on race, ethnicity, or other socially relevant groupings	This study does not involve human participants, their data or their biological material
Population characteristics	This study does not involve human participants, their data or their biological material
Recruitment	This study does not involve human participants, their data or their biological material
Ethics oversight	This study does not involve human participants, their data or their biological material

Note that full information on the approval of the study protocol must also be provided in the manuscript.

Field-specific reporting

Please select the one below that is the best fit for your research. If you are not sure, read the appropriate sections before making your selection.

☒ Life sciences ☐ Behavioural & social sciences ☐ Ecological, evolutionary & environmental sciences

For a reference copy of the document with all sections, see nature.com/documents/nr-reporting-summary-flat.pdf

Life sciences study design

All studies must disclose on these points even when the disclosure is negative.

Sample size	No methods to predetermine sample size for experiments were used. A minimum of 500X coverage per sgRNA was used throughout the whole-genome CRISPR/Cas9-screen to ensure adequate representation of individual sgRNAs. Sample sizes for other experiments were chosen based on data from previous publications (Oh et al., Nature 2020; Yau et al., Cell 2017), robustness of the assay and technical and economical considerations.
Data exclusions	No data were excluded.
Replication	Biological replicates were performed and have been indicated in the figure legends as independent experiments. For experiments without biological replicates, the hypothesis was validated using an alternative experimental setup (different technique, ...) to address the same question.
Randomization	Not applicable, there was no subjective rating of data involved in our study. Randomization is not applicable for most standard cell culture based assays and in vitro biochemical experiments.
Blinding	Not applicable, there was no subjective rating of data involved in our study.

Reporting for specific materials, systems and methods

We require information from authors about some types of materials, experimental systems and methods used in many studies. Here, indicate whether each material, system or method listed is relevant to your study. If you are not sure if a list item applies to your research, read the appropriate section before selecting a response.

Materials & experimental systems

n/a	Involved in the study
☐	☒ Antibodies
☐	☒ Eukaryotic cell lines
☒	☐ Palaeontology and archaeology
☒	☐ Animals and other organisms
☒	☐ Clinical data
☒	☐ Dual use research of concern
☒	☐ Plants

Methods

n/a	Involved in the study
☒	☐ ChIP-seq
☐	☒ Flow cytometry
☒	☐ MRI-based neuroimaging

Antibodies

Antibodies used	Following antibodies were used for immunoblot analyses: anti-Flag (mouse, Clone M2, Sigma-Aldrich, F1804, dilution 1:1000), anti-Flag (rabbit, Cell Signaling Technology (CST), 2368, dilution 1:1000), anti-HA-Tag (rabbit, C29F4, CST, 3724, dilution 1:1000), anti-GAPDH (rabbit, D16H11, CST, 5174, dilution 1:1000), anti-α Tubulin (mouse, DM1A, Calbiochem, CP06) , dilution 1:1000, anti-UBR4/p600 (rabbit, A302, Bethyl, A302-277A, dilution 1:1000), anti-UBR4/p600 (rabbit, A302, Bethyl, A302-278A, dilution 1:1000), anti-UBR4/p600 (rabbit, A302, Bethyl, A302-279A, dilution 1:1000), anti-PKR (mouse, B-10, Santa Cruz, sc-6282, dilution 1:200), anti-GCN2 (mouse, F-7, Santa Cruz, sc-374609, dilution 1:200), anti-PERK (mouse, B-5, Santa Cruz, sc-377400, dilution 1:200), anti-UBE2A/B (mouse, G-9, Santa Cruz, sc-365507, dilution 1:150), anti-ATF4 (rabbit, D4B8, CST, 11815S, dilution 1:1000), anti-EIF2AK1 (rabbit, Proteintech, 20499-1-AP, dilution 1:1000), anti-SSBP1 (rabbit, Proteintech, 12212-1-AP, dilution 1:1000), anti-TIM8A (rabbit, Proteintech, 11179-1-AP, dilution 1:500), anti-KCMF1 (rabbit, Sigma, HPA030383, dilution 1:1000), anti-NIPSNAP3A (rabbit, ThermoFisher, PA5-20657, dilution 1:1000), anti-GADD34 (rabbit, Proteintech 10449-1-AP, dilution 1:1000), anti-CReP (rabbit, Proteintech 14634-1-AP, dilution 1:1000), anti-Ubiquitin (rabbit, Cell Signaling Technology (CST), 43124, dilution 1:1000) goat anti-rabbit IgG (H+L) HRP (Vector Laboratories, PI-1000, dilution 1:5000), Sheep anti-mouse IgG (H+L) HRP (Sigma, A5906, dilution 1:5000), goat anti-mouse IgG light chain specific HRP conjugated (Jackson Immunoresearch, 115-035-174, dilution 1:5000). Following antibodies were used for immunofluorescence: anti-TOM20 antibody (rabbit, Proteintech 11802-1-AP, dilution 1:500), secondary antibody goat anti-rabbit AF647 (ThermoFisher, A21245, dilution 1:500).
Validation	Antibodies validated by knockdown/-out shown in this study: anti-UBR4/p600 (rabbit, A302, Bethyl, A302-277A, validated for WB in human cells, see Extended Data Fig 1a), anti-UBR4/p600 (rabbit, A302, Bethyl, A302-278A, validated for WB in human cells, see Extended Data Fig 1a), anti-UBR4/p600 (rabbit, A302, Bethyl, A302-279A, validated for WB in human cells, see Extended Data Fig 1a), anti-PKR (mouse, B-10, Santa Cruz, sc-6282, validated for WB in human cells, see Extended Data Fig 9a-d), anti-GCN2 (mouse, F-7, Santa Cruz, sc-374609, validated for WB in human cells, see Extended Data Fig 9a-d), anti-PERK (mouse, B-5, Santa Cruz, sc-377400, validated for WB in human cells, see Extended Data Fig 9a-d), anti-EIF2AK1 (rabbit, Proteintech, 20499-1-AP, validated for WB in human cells, see Extended Data Fig 9a-d), anti-TIM8A (rabbit, Proteintech, 11179-1-AP, validated for WB in human cells, see Fig 3c and Extended Data Fig 5e), anti-KCMF1 (rabbit, Sigma, HPA030383, validated for WB in human cells, see Extended Data Fig 10a-c), anti-GADD34 (rabbit, Proteintech 10449-1-AP, validated for WB in human cells, see Extended Data Fig 6c + 6e), anti-CReP (rabbit, Proteintech 14634-1-AP, validated for WB in human cells, see Extended Data Fig 6d and Figure 3f)<br><br>Antibodies validated by manufacturer: anti-Flag (mouse, Clone M2, Sigma-Aldrich, F1804, https://www.sigmaaldrich.com/US/en/product/sigma/f1804, used in 8252 publications, previously validated in our lab on recombinant proteins), anti-Flag (rabbit, Cell Signaling Technology (CST), 2368, https://www.cellsignal.com/products/primary-antibodies/dykddddk-tag-antibody-binds-to-same-epitope-as-sigma-s-anti-flag-m2-antibody/2368, used in 722 publications, previously validated in our lab on recombinant proteins), anti-HA-Tag (rabbit, C29F4, CST, 3724, https://www.cellsignal.com/products/primary-antibodies/ha-tag-c29f4-rabbit-mab/3724, used in 2406 publications, previously validated in our lab on recombinant proteins), anti-GAPDH (rabbit, D16H11, CST, 5174, https://www.cellsignal.com/products/primary-antibodies/gapdh-d16h11-xp-rabbit-mab/5174, used in 5800 publications, detected protein at correct size in this study), anti-α Tubulin (mouse, DM1A, Calbiochem, CP06, https://www.sigmaaldrich.com/US/en/product/mm/cp06, used in 673 publications, detected protein correct size in this study), anti-UBE2A/B (mouse, G-9, Santa Cruz, sc-365507, https://www.scbt.com/p/ube2a-b-antibody-g-9, used in >10 publications, detected protein at correct size in this study), anti-ATF4 (rabbit, D4B8, CST, 11815S, https://www.cellsignal.com/products/primary-antibodies/atf-4-d4b8-rabbit-mab/11815, used in 703 publications, detected protein at correct size and induced upon ISR induction in this study), anti-NIPSNAP3A (rabbit, ThermoFisher, PA5-20657, https://www.thermofisher.com/antibody/product/NIPSNAP3A-Antibody-Polyclonal/10751-1-AP, validated by manufacturer for IP, IF, WB and IHC, detected protein at correct size in this study), , anti-Ubiquitin (rabbit, Cell Signaling Technology (CST), 43124,https://www.cellsignal.com/products/primary-antibodies/ubiquitin-e4i2j-rabbit-mab/43124, used in 42 publications, recognizes endogenous levels of free ubiquitin and polyubiquitinated proteins. This antibody is able to detect free ubiquitin, linear polyubiquitin (M1-linked), and homotypic polyubiquitin chains consisting of K6, K11, K27, K29, K33, K48 and K63 linkages. validated by manufacturer for western blotting applications.), anti-TOM20 antibody (rabbit, Proteintech 11802-1-AP, https://www.ptglab.com/products/TOM20-Antibody-11802-1-AP.htm, used in 224 publications, specifically stained outer mitochondrial membrane in this study).

Eukaryotic cell lines

Policy information about cell lines and Sex and Gender in Research

Cell line source(s)	HEK293T and U2OS cells were purchased from the Berkeley Cell Culture Facility. iPSCs originated from the lab of M. Ward (NIH). Male human embryonic stem cells (ESCs) line H1: WiCell Research Institute, Inc. (WA01).
Authentication	All cell lines were authenticated by short tandem repeat analysis.

Mycoplasma contamination	All cell lines were routinely tested biweekly for mycoplasma contamination using the Mycoplasma PCR Detection Kit (abm, G238) and consistently tested negative.
Commonly misidentified lines (See ICLAC register)	No commonly misidentified cell lines were used in this study.

Flow Cytometry

Plots

Confirm that:

☒ The axis labels state the marker and fluorochrome used (e.g. CD4-FITC).

☒ The axis scales are clearly visible. Include numbers along axes only for bottom left plot of group (a 'group' is an analysis of identical markers).

☒ All plots are contour plots with outliers or pseudocolor plots.

☒ A numerical value for number of cells or percentage (with statistics) is provided.

Methodology

Sample preparation	HEK293T cells were trypsinized and resuspended in PBS for flow cytometry.
Instrument	BD LSR Fortessa, BD LSR Fortessa X20
Software	FACSDiva (Version 9.0), FlowJo (Version 10.8.1)
Cell population abundance	For all assays, cells populations were determined using fluorescent markers. Non-fluorescent populations were clearly separated from those with fluorescent markers. Sufficient events were ensured for all populations.
Gating strategy	Initial gating steps include identification of live cells (SSC-A/FSC-A) followed by identification of single cells (FSC-H/FSC-A). The further gating strategy depends on the experimental setup:<br>- For cell competition assays mCherry+ and GFP+ populations were determined and ratios were calculated.<br>- For protein stability assays a derived parameter (GFP/mCherry) was plotted as a histogram to the mode within the GFP+/mCherry+ population to represent stability of GFP-tagged proteins controlled by mCherry expression ensured an IRES.<br>- For mitochondrial protein import assays in the next gating step, mScarlett+ cells (stably expressing MTS-GFP1-10) were selected. Then, a derived parameter (GFP/BFP) was plotted as a histogram to the mode within the GFP+/BFP+ population to represent mitochondrial import measured by reconstitution of GFP controlled to BFP expression ensured by an IRES.

☒ Tick this box to confirm that a figure exemplifying the gating strategy is provided in the Supplementary Information.
